# Synthesis of *N*-Bromo and *N*-Iodo Imides: A Rapid Redox-Neutral and Bench Stable Process

**DOI:** 10.1021/acs.oprd.4c00194

**Published:** 2024-10-11

**Authors:** Ankush Chakraborty, Bardia Soltanzadeh, Nicholas R. Wills, Arvind Jaganathan, Babak Borhan

**Affiliations:** Department of Chemistry, Michigan State University, East Lansing, Michigan 48824, United States

## Abstract

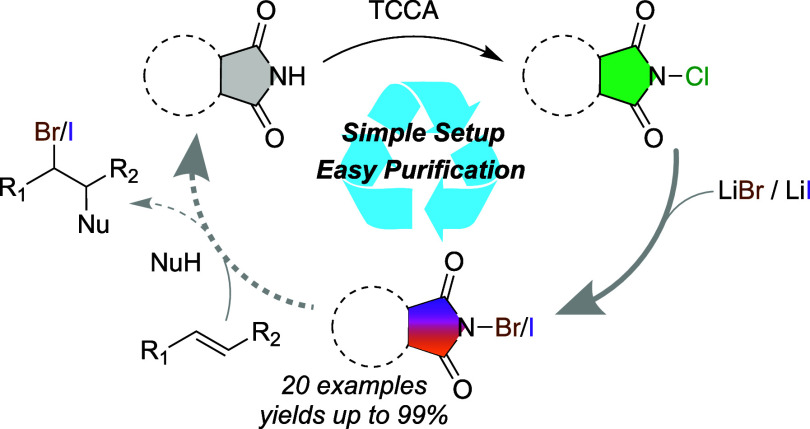

This report presents a rapid, ecofriendly technique for the formation of commonly used *N*-bromo and *N*-iodinating reagents by reacting readily available *N*-chloro derivatives with inorganic bromide and iodide salts. All reagents were easily handled, commercially available, and bench stable. This strategy illustrates the expeditious formation of these halogenating reagents in multigram scale in high-yields and purity with an operationally straightforward recrystallization. The mechanistic details suggest an in situ generation of an interhalogen species.

*N-*Halogenating agents are widely employed in academia and industry.^1−4^ They serve as stable, easily handled sources of halenium ions and obviate the need to use more corrosive reagents such as molecular chlorine, bromine, or iodine. Recent developments in organocatalysis have introduced stereocontrolled halogenation—further expanding the utility of haleniums in synthesis.^1−4^ Among these, *N*-bromo and *N*-iodo imides are particularly useful owing to the higher reactivity of the resulting bromide or iodide products. Halenium sources are also used as disinfectants, and antimicrobial and antibacterial agents because of their potent oxidative activity with high utility in the processing and packaging industry.^5−7^ For example, 1,3-dibromodimethylhydantoin (DBDMH), marketed as AviBrom, BoviBrom and PorciBrom (for birds, cows, and pigs processing, respectively) is utilized by the food industry.^5−7^ In academia, these reagents have found tremendous application in organic synthesis specially in the fields of radical halogenation,^8^ electrophilic aromatic halogenations,^9,10^ oxidation of thiol to disulfides,^11^ and most recently in enantioselective halofunctionalization of alkenes.^12,13^

Two methods are prominently used for the synthesis of *N*-haloimides (Figure 1a): (a) treatment of the parent imide with molecular bromine or iodine in the presence of a strong base;^14^ and (b) *in situ* generation of Br_2_ or I_2_ using readily available bromide or iodide salts and a strong oxidant.^15,16^ These processes have precautionary measures for small scale reactions but require special handling on industrial scale because of the generation of corrosive Br_2_ or I_2_, and the hazardous nature of the oxidants (H_2_SO_4_, H_2_O_2_, persulfate salts, oxone, hypervalent iodine, etc.).^17,18^ Also, most often the extruded product from iodinating agents generate iodide salts that require wastewater treatment before being released. The latter discussion illustrates the potential benefits for the *in situ* generation of bromo- and iodinating agents from their corresponding chloro-substituted analogues under mild reaction conditions, thus avoiding the need for the transport and handling of highly corrosive reagents.

**Figure 1:**
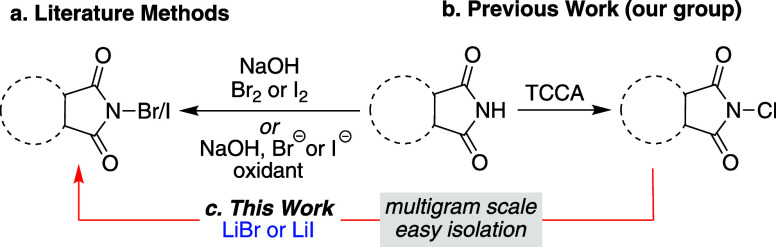
(a) Commonly used method for producing *N*-haloimides. (b) An expeditious procedure to obtain *N*-chloroimides. (c) Simple conversion of *N*-chloroimides to the corresponding bromo and iodo analogues.

Our group has previously demonstrated the conversion of amides and imides to their corresponding *N*-chlorinated analogues (Figure 1b).^19^ The present work provides a rapid, and cost-efficient procedure to generate the *N*-bromo and *N*-iodo amides and imides by reacting the readily available *N*-chloro analogues with inorganic bromide or iodide salts (LiBr, LiI), producing LiCl as the side product. All reagents were easily handled, bench stable and the final halogenating agents were purified by a simple recrystallization.

We have previously reported the enantioselective vicinal dihalogenation of allyl amides in the presence of (DHQD)_2_PHAL as the chiral organocatalyst. An electrophilic halenium (X^+^) donor along with lithium halide (X^–^) was required for the completion of this transformation.^20^ In addition to vicinal dichlorination and dibromination, heterodihalogenation i.e., bromo-chlorination of the allyl amide was demonstrated (Figure 2a). Specifically, when **1** was treated with LiCl as the nucleophilic chloride source and DBDMH as the source of electrophilic bromenium, chloro-brominated product **2** was formed in 97% yield. In an effort to reverse the regiochemistry, LiBr was used as the nucleophilic halogen source along with the chlorenium donating reagent DCDMH for accessing the bromo-chlorinated product **3** (regioisomer of **2**). To our surprise, instead of the anticipated mixed halogenated product, the dibrominated product **4** was isolated in 96% yield (Figure 2a). It was surmised that DCDMH reacted with LiBr *in situ* to afford DBDMH, thus generating the bromenium that apparently leads to the observed product. ^13^C NMR analysis confirmed that the treatment of DCDMH with a slight excess of LiBr resulted in the quantitative formation of DBDMH (Figure 2b). A thorough search of the literature for similar transhalogenations revealed prior art for the conversion of NCS to NBS^21^ and NCS to NIS^22^ using tetraethylammonium bromide and sodium iodide, respectively. Considering the varying potency of *N*-bromo and *N*-iodohaloimides, and the results we had obtained with chlorohydantoins, we sought to examine transhalogenation of various chlorinating reagents to their bromo and iodo analogues.

**Figure 2:**
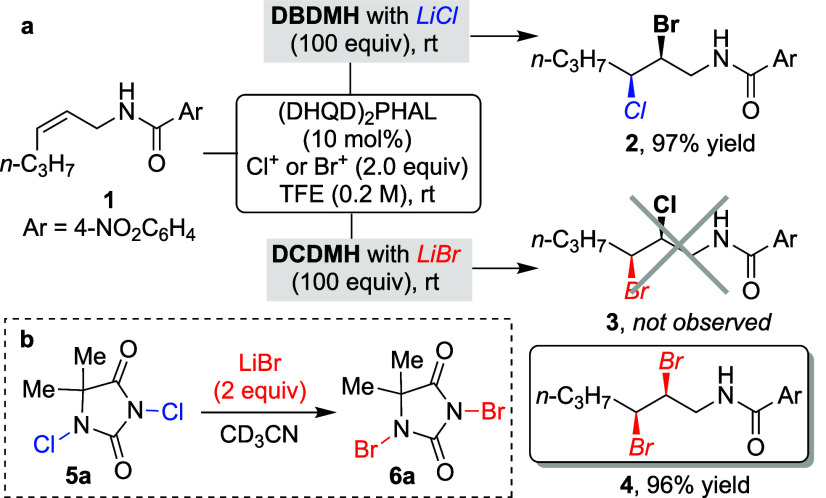
(a) Regioselective chloro-bromination of allyl amides with bromenium and chloride yielding the expected product **2**, while with chlorenium and bromide producing the dibrominated product **4** instead of the expected product **3**; (b) LiBr-mediated transformation of DCDMH to DBDMH.

It occurred to us that this might present a general route to access a variety of *N*-bromoimides from the corresponding *N*-chloroimides. A short optimization of different reaction parameters (Table 1) demonstrated that LiBr in slight excess relative to DCDMH with acetonitrile as the optimal solvent yields DBDMH in near quantitative conversion (Table 1, entry 3), reproducible on a 10 g scale. Recrystallization from CHCl_3_/hexane or EtOAc/hexane yields the product as snow-white needles.

**Table 1:**
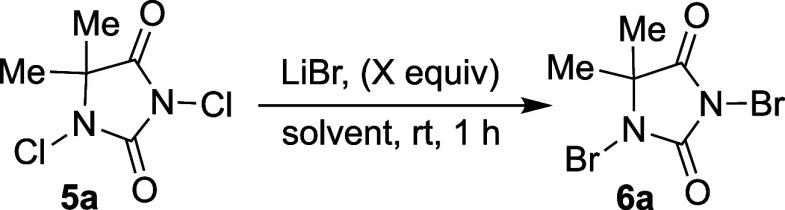
Optimization of Reaction Parameters for *N*-Bromoimides

Entry^a^	Equiv. LiBr	Solvent	Yield (%)^b^
1	2.0	CH_3_CN	92
2^c^	2.0	CH_3_CN	76
3	2.2	CH_3_CN	99(95)^d^
4	2.5	CH_3_CN	98
5	3.0	CH_3_CN	96
6	2.2	DCM	67
7	2.2	PhMe	56
8^e^	2.2	CH_3_CN	78

a All reactions were performed with 5.0 mmol of **5a** at 0.1 M.

b Isolated yields.

c NaBr was used as the halogenating agent.

d 10 g scale.

e Reaction was performed at 50 °C.

With this result in hand, we sought to understand the generality of this reaction for the synthesis of other commonly used *N*-bromo and *N*-iodoimides (Figure 3). Initially, different chloro-hydantoins were reacted under conditions that resulted in the formation of their corresponding bromo-analogues in near quantitative yields. Exposing commercially available chloro-hydantoins (**6a**, **6b**), chloro-imides (**6d**, **6e**), chloro-amide (**6f**), and chloro-sulfonamides (**6i**, **6j**) afforded their respective bromo-analogues in excellent yields (>95%). Select *N*-chloro halogenating agents, dichlorohydantoin (DCH), *N*-chloropyrollidinone (NCPyl), and trichloroisocyanuric acid (TCCA) had a slightly reduced efficiency in delivering product (**6c**, **6g**, and **6h**, respectively), with yields ranging from 82 to 88%.

**Figure 3:**
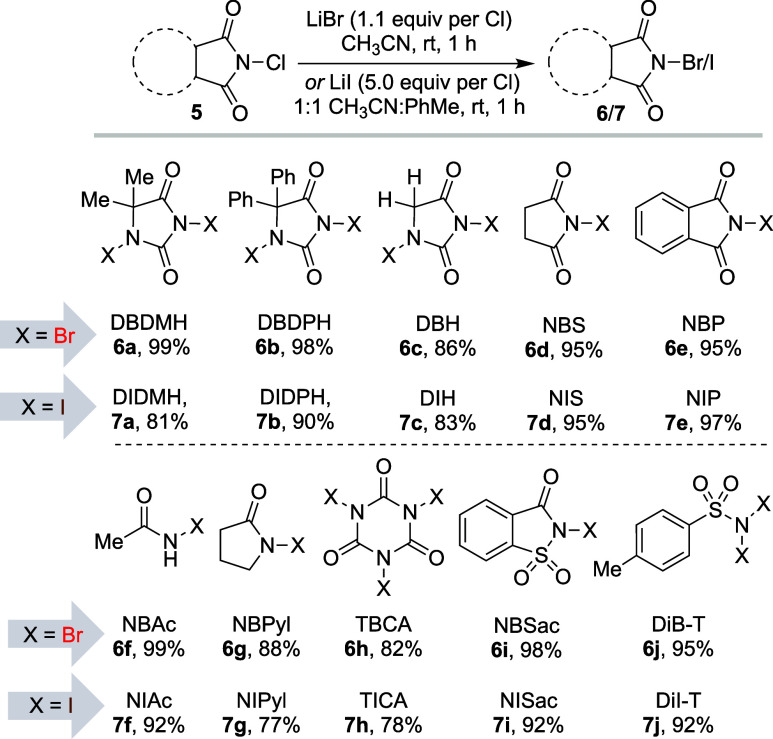
Substrate scope for the formation of *N*-bromo- and *N*-iodoimides. All reactions (isolated yields provided in the figure) were performed with 5.0 mmol of substrate (0.1 M).

Recognizing the potential of this approach, the same protocol was examined for *N*-iodo analogues; utilizing LiI *in lieu* of LiBr. Arguably, these *N*-iodo halogenating agents are in greater demand for subsequent synthetic transformations considering the plethora of reports that exploit the reactivity of C–I bonds. Excluding *N*-iodosuccinimide, other iodinating agents are costly and decompose readily inside a newly sealed bottle. An efficient protocol to generate these would offer great potential for synthetic applications.

Table 2 details the results of optimization studies to generate NIS from NCS. The standard bromination conditions were not high yielding when translated to iodination. Unlike the protocol for *N*-bromo analogues, high yields of the *N*-iodo variants were obtained when an excess amount (5.0 equiv) of LiI was used in a solvent mixture of toluene and acetonitrile (Table 2, entry 8). Notably, the classical Finkelstein conditions^23^ failed to yield optimal results for both bromo and iodo analogues (Table 1, entry 2; Table 2, entry 11). With the optimized conditions, NIS was successfully synthesized on a 10 g scale with little erosion in yield. For a complete assessment, the aforementioned conditions were applied to the remaining *N*-chloro derivatives (Figure 3). Iodination for *N*-chlorohydantoins (**7a**, **7b**, **7c**), *N*-chloroimides (**7d**, **7e**), *N*-chloroamide (**7f**), and *N*-chlorosulfinyls (**7i**, **7j**) underwent smooth transformation to the corresponding iodo analogues in high yields (81–97%). Likewise, *N*-chloropyrollidinone and TCCA afforded moderate to good yields of the *N*-iodo products **7g** and **7h**. These products were recrystallized from hot dioxane/hexane to generate the purified iodinating agents.

**Table 2:**
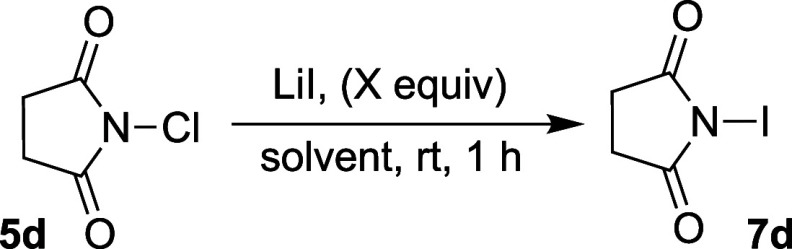
Optimization of Reaction Parameters for *N*-Iodoimides

Entry^a^	Equiv. LiI	Solvent	Yield (%)^b^
1	1.1	CH_3_CN	57
2	2.0	CH_3_CN	62
3	2.5	CH_3_CN	70
4	5.0	CH_3_CN	83
5^c^	10.0	CH_3_CN	80
6	5.0	DCM	40
7	5.0	PhMe	80
8	5.0	CH_3_CN:PhMe (1:1)	95(92)^d^
9	5.0	CH_3_CN:PhMe (1:2)	95
10^e^	5.0	CH_3_CN:PhMe (1:1)	66
11^f^	5.0	CH_3_CN:PhMe (1:1)	60

a All reactions were performed with 5.0 mmol of **5d** at 0.1 M.

b Isolated yields.

c Concentration was 0.01 M.

d 10 g scale.

e Reaction was performed at 50 °C.

f NaI in place of LiI.

It is noted the enthalpy of formation for lithium salts increases from LiCl > LiBr > LiI. Similar to the Finkelstein reaction, we propose the generation and precipitation of LiCl is the major driving force toward product formation. To test this, the following observations were made. First, exposure of NBS or NIS to a great excess of LiCl (100 equiv) does not yield any discernible levels of NCS (Figure 4a). Nonetheless, *N*-iodoimides could be synthesized by the addition of LiI to their respective *N*-bromo derivatives under the same conditions (for example NIS from NBS, see Figure 4b), but not *vice versa*. Second, reaction of NCS with LiBr or LiI seems to proceed via the *in situ* generation of BrCl or ICl, respectively, as evidence by the following observation. NCS and LiBr were mixed for 5 min, followed by the addition of either styrene **8a** or 4-methoxystyrene **8b**, which led to the production of the heterodihalogenated product **9a** or **9b**, respectively (Figure 4c). The regiochemistry of the product, assuming a Markovnikov addition of the halenium followed by trapping of the ensuing carbocation by the halide suggest that the bromide from LiBr was in fact converted to the bromenium to yield the observed products. Conversely, if the chlorenium from NCS had retained its electrophilic nature, one would expect product **10a** or **10b**. The same trend was observed with the reaction of **8a** and **8b** with premixed LiI/NCS to produce **11a** and **11b**, and not **12a**/**12b** (Figure 4c). With these observations in hand, depicted in Figure 4d is a plausible mechanism that initiates with the attack of the halide ion to generate an interhalogen compound (XCl). The resulting imide ion would then capture the more electrophilic halogen (I^+^ or Br^+^) to afford the respective *N*-iodo or *N*-bromo analogue.

**Figure 4:**
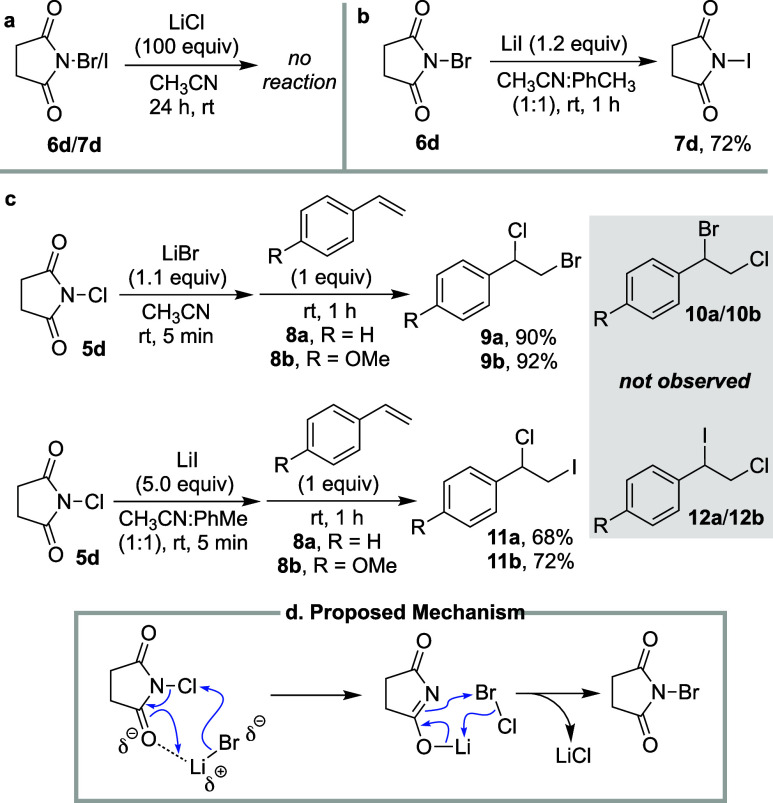
(a) NBS and NIS do not react with excess LiCl. (b) NBS can be converted to NIS under similar conditions used for conversion of NCS to NIS. (c) Regioselection in halofunctionalization of styrene and 4-methoxystyrene suggest the intermediacy of an interhalogen species. (d) The proposed mechanism for halogen exchange.

In the review of this manuscript, a one-pot conversion of imides and amides to *N*-bromo and *N*-iodo counterparts was suggested. Telescoping reactions of succinimide to generate NCS with TCCA, followed by its conversion to NBS and NIS are shown in Figure 5. The crude mixtures indicate incomplete conversion of the second step, with a large excess of NCS (7:1 NCS:NBS, and 10:1 NCS:NIS) remaining after addition of the lithium salts. We appreciate the benefits of a one-pot method, although in this case, depreciation in yield, and the presence of unreacted NCS present challenges that needs to be addressed for it to be a feasible approach.

**Figure 5:**
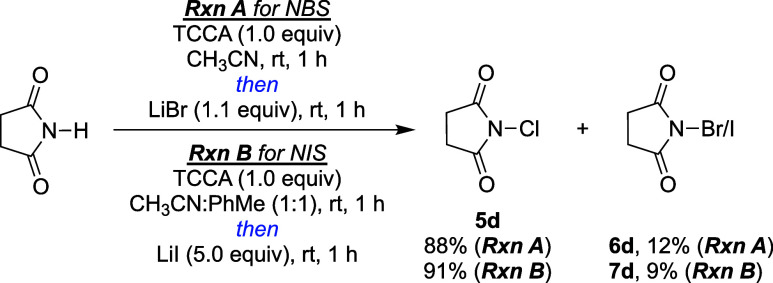
One-pot halogenation of succinimide to NBS or NIS using optimal conditions from Tables 1 and 2. Ratio of final products were obtained from crude ^1^H NMR.

In conclusion, we have presented an operationally simple method for the preparation of several *N*-bromo and *N*-iodo- reagents using their readily available chloro analogues, which can be obtained either commercially or via a simple chlorination from the corresponding amides or imides. The products are isolated in the crystalline form in high yields without recourse to chromatographic purification. We also catalog physical data for a number of these halogenating agents not previously undisclosed in the literature. Presumably, the reaction proceeds via the *in situ* generation of an interhalogen species.

## Supporting Information Available

Experimental details, spectroscopic data, and crystal structure factors (PDF)
