# Supplementary material for: Synthesis of N-Bromo and N-Iodo Imides: A Rapid Redox-Neutral and Bench Stable Process
Source: Org Process Res Dev. 2024 Oct 11;28(11):3959–62. doi: 10.1021/acs.oprd.4c00194 (PMC11574840; doi:10.1021/acs.oprd.4c00194)

SUPPORTING INFORMATION

Synthesis of *N*-Bromo and *N*-Iodo imides: A Rapid Redox-Neutral and Bench Stable Process

Ankush Chakraborty, Bardia Soltanzadeh, Nicholas R. Wills, Arvind Jaganathan, Babak Borhan*

Department of Chemistry, Michigan State University, East Lansing, Michigan 48824.

Corresponding author's address:
Michigan State University
Department of Chemistry
578 S. Shaw Ln
East Lansing, MI 48824
babak@chemistry.msu.edu

General Experimental 3
General Procedure for the Synthesis of *N*-Chlorinating Agents (GP I): 4
General Procedure for the Synthesis of *N*-Brominating Agents (GP II): 12
Analytical Data for the *N*-Bromo halogenating agents: 13
General Procedure for the Synthesis of *N*-iodinating Agents (GP III): 20
Analytical Data for the N-Iodo halogenating agents: 20
Mechanistic Experiments: 28
Crystal Structures: 32
References 38
NMR Spectrum for *N*-halogenating agents: 39

General Experimental

All commercially available hydantoins and trichloroisocyanuric acid (TCCA) were purchased from Aldrich and used without purification. Solvents were purchased from either Fisher Scientific or Oakwood Chemicals and used without purification. Melting points were recorded on a Thomas Hoover capillary melting point apparatus. Agilent Technologies 500/54 Premium Shielded 500 MHz spectrometer was used to record the ^{1}H NMR and ^{13}C NMR spectra using $CDCl_3$ as solvent. The residual peak of $CDCl_3$ or TMS was used as the internal standard for both ^{1}H NMR (δ = 7.26 ppm for $CDCl_3$ or δ = 0 ppm for TMS) and ^{13}C NMR (δ = 77.16 ppm). Chemical shifts were reported in parts per million (ppm). Analytical thin-layer chromatography (TLC) was performed on Silicycle silica gel plates with F-254 indicator. Visualization was by short wave (254 nm) and long wave (365 nm) ultraviolet light, or by staining with phosphomolybdic acid in ethanol. Column chromatography was performed with silica gel 60 (230 – 450 mesh). Infrared spectra were recorded on NaCl disc (for liquids) on a Nicolet IR/42 spectrometer or on a JASCO FT/IR-6600. Samples were prepared as KBr pellets. High Resolution Mass Spectrometry was performed in the Department of Chemistry at Michigan State University Mass Spec Facility.

General Procedure for the Synthesis of *N*-Chlorinating Agents (GP I):

N-Chloroimides were synthesized following previously developed method from our lab.[1] In a clean dry 100 mL round bottom substrate **S1** (5.0 mmol) was suspended in acetonitrile (20 mL) solvent. TCCA (5.0 mmol, 1.16 g, 1.0 equiv. per H) was then quickly added in one portion and the resulting mixture was stirred at room temperature for an hour. When the heterogenous mixture became clear it was concentrated under reduced pressure to generate a white precipitate. The solids were mounted on top of a 50 mL fretted funnel containing a silica plug (5 cm). The solids were then filtered with suction in $CHCl_3$ (50 mL). The organics were concentrated under reduced pressure and the solids were recrystallized by dissolving in a minimum amount of $CHCl_3$ and hexane (three times the volume of $CHCl_3$) was layered on top of chloroform and cooled at –20° C. The crystals were collected by suction filtration and dried under high vacuum for an hour. These solids were used for recording analytical data and subsequently for the synthesis of the corresponding N-bromo/N-iodo derivatives.

Analytical Data for the *N*-Chloro halogenating agents:

Scheme S1: Preparation of DCDMH (**5a**)

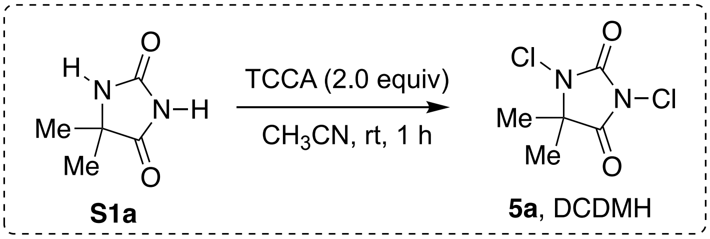

N-chlorohydantoin **5a** was prepared from dimethylhydantoin **S1a** (640.7 mg, 5.0 mmol) and TCCA (2.32 g, 10.0 mmol) using GP I. The crude product was recrystallized from $CHCl_3$: Hexane (1:3) to obtain pure product **5a** (788 mg, 4.0 mmol) as white needles in 80% yield.

Spectral Data for 1,3-dichloro-5,5-dimethylimidazolidine-2,4-dione (DCDMH): ^{1}H NMR (500 MHz, $CDCl_3$) δ 1.55 (s, 6H). ^{13}C NMR (126 MHz, $CDCl_3$) δ 169.95, 150.43, 68.06, 22.62. IR (cm $^{-1}$): 1799, 1750, 1734, 1455, 1340, 1203, 1128, 955, 869, 782, 712. mp: 135 - 138 °C.[1] HRMS: TOF MS ES$^+$ ($C_5H_6Cl_2N_2O_2$): Calc. [M + H]$^+$: 193.9884, Found [M + H]$^+$: 193.9887.

Scheme S2: Preparation of DCDPH (**5b**)

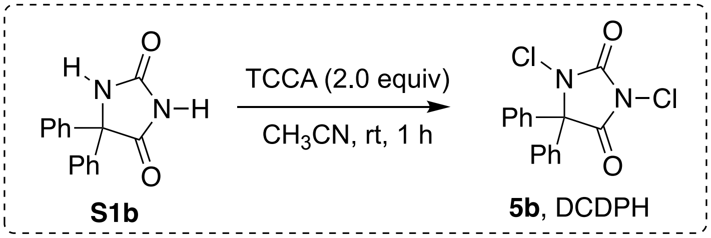

N-chlorohydantoin **5b** was prepared from diphenylhydantoin **S1b** (1.26 g, 5.0 mmol) and TCCA (2.32 g, 10.0 mmol) using GP I. The crude product was recrystallized from $CHCl_3$: Hexane (1:3) to obtain pure product **5a** (1.45 g, 4.5 mmol) as white needles in 90% yield.

Spectral Data for 1,3-dichloro-5,5-diphenylimidazolidine-2,4-dione (DCDPH): ^{1}H NMR (500 MHz, $CDCl_3$) δ 7.51 – 7.40 (m, 6H), 7.33 (dd, *J* = 8.1, 1.7 Hz, 4H). ^{13}C NMR (126 MHz, $CDCl_3$) δ 167.22, 150.58, 134.58, 130.05, 129.03, 128.47, 79.35. IR (cm $^{-1}$): 1795, 1745, 1213, 701. mp: 156 - 160 °C.[1] HRMS: TOF MS ES^+ ($C_{15}H_{10}Cl_2N_2O_2$): Calc. $[M + H]^+$: 321.0194, Found $[M + H]^+$: 321.0198.

Scheme S3: Preparation of DCH (**5c**)

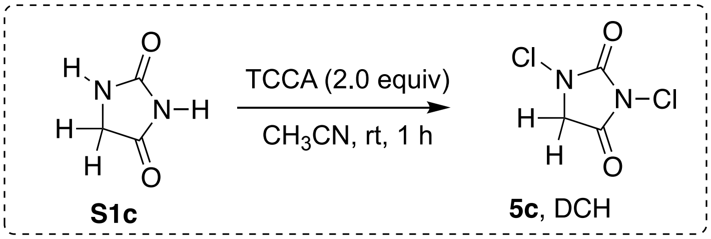

N-chlorohydantoin **5c** was prepared from hydantoin **S1c** (500.4 mg, 5.0 mmol) and TCCA (2.32 g, 10.0 mmol) using GP I. The crude product was recrystallized from $CHCl_3$: Hexane (1:3) to obtain pure product **5a** (659.1 mg, 3.9 mmol) as white solid in 78% yield.

Spectral Data for 1,3-dichloroimidazolidine-2,4-dione (DCH): ^{1}H NMR (500 MHz, $CDCl_3$) δ 4.32 (s, 2H). ^{13}C NMR (126 MHz, $CDCl_3$) δ 162.78, 152.67, 56.70. IR (cm $^{-1}$): 1738, 1356, 1252, 1188, 1032, 754. mp: 115 - 117 °C.[1] HRMS: TOF MS ES^+ ($C_3H_2Cl_2N_2O_2$): Calc. $[M + H]^+$: 168.9571, Found $[M + H]^+$: 168.9570.

Scheme S4: Preparation of NCS (**5d**)

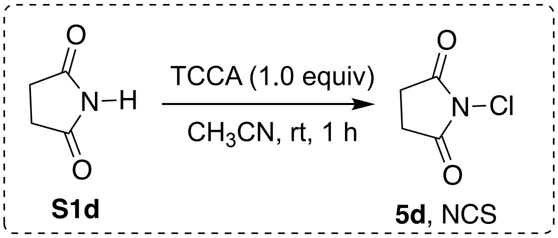

N-chlorosuccinimide **5d** was prepared from imide **S1d** (495.4 mg, 5.0 mmol) and TCCA (1.16 g, 5.0 mmol) using GP I. The crude product was recrystallized from $CHCl_3$: Hexane (1:3) to obtain pure product **5a** (614.2 mg, 4.6 mmol) as white crystalline in 92% yield.

Spectral Data for 1-chloropyrrolidine-2,5-dione (NCS): ^1H NMR (500 MHz, $CDCl_3$) δ 2.91 (s, 4H). ^{13}C NMR (126 MHz, $CDCl_3$) δ 171.28, 28.01. IR (cm $^{-1}$): 1707, 1424, 1323, 1153, 961, 813, 646. mp: 148-150 °C.[1] HRMS: TOF MS ES^+ ($C_4H_4ClNO_2$): Calc. $[M + H]^+$: 134.0009, Found $[M + H]^+$: 134.0001.

Scheme S5: Preparation of NCP (**5e**)

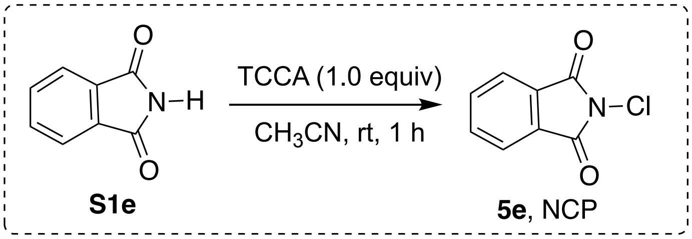

N-chloropthalimide **5e** was prepared from imide **S1e** (735.7 mg, 5.0 mmol) and TCCA (1.16 g, 5.0 mmol) using GP I. The crude product was recrystallized from $CHCl_3$: Hexane (1:3) to obtain pure product **5e** (835.2 mg, 4.6 mmol) as white crystalline in 92% yield.

Spectral Data for 2-chloroisoindoline-1,3-dione (NCP): ^{1}H NMR (500 MHz, $CDCl_3$) δ 7.89 (dd, *J* = 5.5, 3.1 Hz, 2H), 7.78 (dd, *J* = 5.5, 3.1 Hz, 2H). ^{13}C NMR (126 MHz, $CDCl_3$) δ 163.39, 134.79, 131.11, 124.03. IR (cm $^{-1}$): 3091, 1738, 1704, 1607, 1466, 1349, 1296, 1058, 855, 696. mp: 187-188 °C. HRMS: TOF MS ES^+ ($C_8H_4ClNO_2$): Calc. $[M + H]^+$: 182.0009, Found $[M + H]^+$: 182.0011.

Scheme S6: Preparation of NCPyr (**5f**)

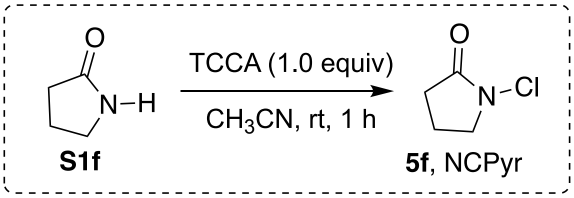

CCDC no: 2225525

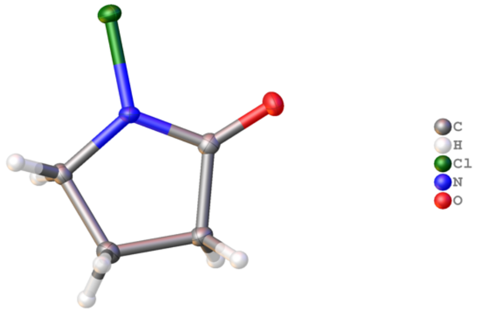

N-chloroamide **5f** was prepared from amide **S1f** (425.5 mg, 5.0 mmol) and TTCA (1.16 g, 5.0 mmol) using GP I. The crude product was recrystallized from $CHCl_3$: Hexane (1:3) to obtain pure product **5f** (406.5 mg, 3.4 mmol) as white crystalline in 68% yield.

Spectral Data for 1-chloropyrrolidin-2-one (NCPyr): ^{1}H NMR (500 MHz, $CDCl_3$) δ 3.59 (td, *J* = 7.0, 1.6 Hz, 2H), 2.40 (ddd, *J* = 8.9, 7.6, 1.9 Hz, 2H), 2.24 – 2.12 (m, 2H). ^{13}C NMR (126 MHz, $CDCl_3$) δ 173.00, 52.22, 27.78, 18.20. IR (cm $^{-1}$): 1685, 1419. mp: 155 °C (decomp). HRMS: TOF MS ES^+ (C_4H_6ClNO): Calc. $[M + H]^+$: 120.0216, Found $[M + H]^+$: 120.0218.

Scheme S7: Preparation of NCAc (**5g**)

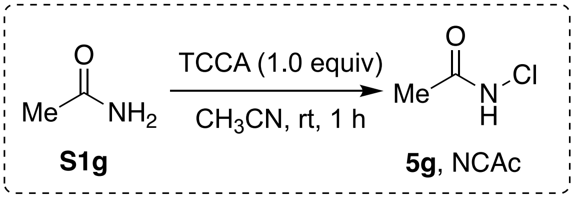

N-chloroacetamide **5g** was prepared from amide **S1g** (295.3 mg, 5.0 mmol) and TCCA (1.16 g, 5.0 mmol) using GP I. The crude product was recrystallized from $CHCl_3$: Hexane (1:3) to obtain pure product **5g** (411.4 mg, 4.4 mmol) as white crystalline in 88% yield.

Spectral Data for *N*-chloroacetamide (NCAc): ^{1}H NMR (500 MHz, $CDCl_3$) δ 7.51 (s, 1H), 2.17 (s, 3H). ^{13}C NMR (126 MHz, $CDCl_3$) δ 169.44, 21.79. IR (cm $^{-1}$): 2993, 1691, 784. mp: 110-111 °C.[1] HRMS: TOF MS ES^+ (C_2H_4ClNO): Calc. $[M + H]^+$: 94.0059, Found $[M + H]^+$: 94.0062.

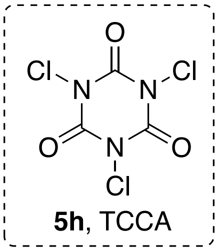

Spectral Data for 1,3,5-trichloro-1,3,5-triazinane-2,4,6-trione (TCCA): ^{13}C NMR (126 MHz, CD_3CN) δ 145.31. IR (cm $^{-1}$): 1730. mp: 244-246 °C.

Scheme S8: Preparation of DIC-T (**5j**)

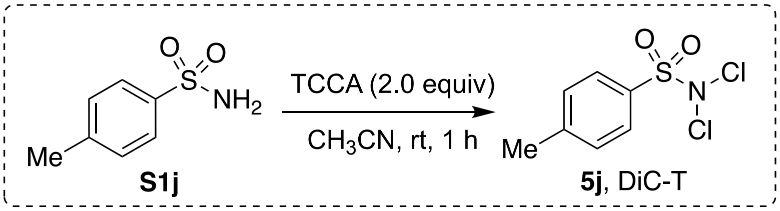

N-chlorosaccharin **5i** was prepared from amide **S1i** (915.9 mg, 5.0 mmol) and TTCA (1.16 g, 5.0 mmol) using GP I. The crude product was recrystallized from $CHCl_3$: Hexane (1:3) to obtain pure product **5i** (870.5 mg, 4.0 mmol) as white solid in 80% yield.

Spectral Data for 2-chlorobenzo[*d*]isothiazol-3(2*H*)-one 1,1-dioxide (NCSac): ^{1}H NMR (500 MHz, $CDCl_3$) δ 8.13 (dt, *J* = 7.5, 0.9 Hz, 1H), 8.01 – 7.97 (d, *J* = 7.8, 1.6 Hz 1H), 7.96 (td, *J* = 7.5, 1.3 Hz, 1H), 7.91 (td, *J* = 7.4, 1.5 Hz, 1H). ^{13}C NMR (126 MHz, $CDCl_3$) δ 156.75, 137.80, 135.66, 135.08, 126.59, 125.91, 121.88, 121.87. IR (cm $^{-1}$): 3090, 1735, 1589, 1455, 1358, 1287, 1230, 1135, 952, 747. mp: 143-144 °C. HRMS: TOF MS ES$^+$ ($C_7H_4ClNO_3S$): Calc. [M + H]$^+$: 217.9678, Found [M + H]$^+$: 217.9679.

Scheme S9: Preparation of NCSac (**5i**)

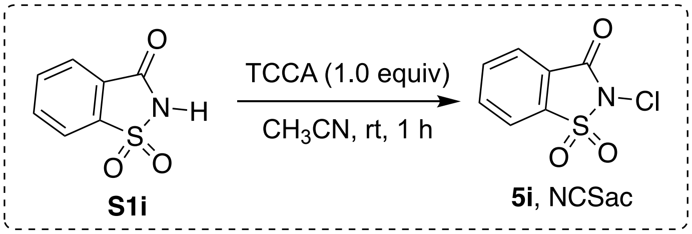

N-chlorosulfonylamide **5j** was prepared from amide **S1j** (856.1 mg, 5.0 mmol) and TTCA (2.32 g, 10.0 mmol) using GP I. The crude product was recrystallized from $CHCl_3$: Hexane (1:3) to obtain pure product **5j** (870.6 mg, 3.65 mmol) as white solid in 73% yield.

Spectral Data for Tosylchlorimidous chloride (DiC-T): ^{1}H NMR (500 MHz, $CDCl_3$) δ 7.98 (d, *J* = 8.4 Hz, 2H), 7.47 (d, *J* = 7.8 Hz, 2H), 2.53 (s, 3H). ^{13}C NMR (126 MHz, $CDCl_3$) δ 147.88, 131.71, 130.07, 125.80, 22.10. IR (cm $^{-1}$): 1322. mp: 84-85 °C. HRMS: TOF MS ES$^+$ ($C_7H_7Cl_2NO_2S$): Calc. $[M + H]^+$: 239.9653, Found $[M + H]^+$: 239.9655.

General Procedure for the Synthesis of *N*-Brominating Agents (GP II):

In a clean dry 100 mL round bottom was introduced LiBr (5.5 mmol, 0.48 g) in acetonitrile (30 mL) as solvent. The round bottom was covered with Al-foil and the solids were stirred for 5 minutes till it became completely soluble in the acetonitrile. General substrate 5 (5.0 mmol) was then slowly added over a couple of minutes and stirred vigorously at room temperature for an hour (once the addition is complete, within 5-10 minutes, there is a formation of an orange-yellow precipitate). The reaction was quenched by the addition of water (5 mL) and further diluted by adding ethyl acetate (5 mL). The organic layer was separated, and the aqueous layer was extracted with ethyl acetate (3 X 5 mL). The combined organics were dried over anhyd. Na_2SO_4 and concentrated under reduced pressure. The solids were dissolved in a minimum amount of $CHCl_3$ and hexane (five times the volume of $CHCl_3$) was layered on top of chloroform and cooled at –20° C (5:1 hexane:EtOAc can be used for crystallization as well, although it yields smaller crystals). The crystals were collected by suction filtration and dried under high vacuum for an hour. These solids were used for recording analytical data.

Analytical Data for the *N*-Bromo halogenating agents:

Scheme S10: Preparation of DBDMH (**6a**)

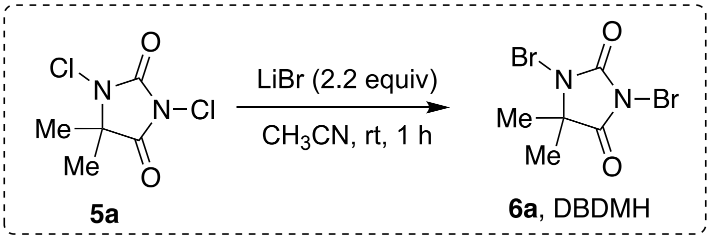

N-bromohydantoin **6a** was prepared from dichlorodimethylhydantoin **5a** (0.98 g, 5.0 mmol) and LiBr (0.96 g, 11.0 mmol) using GP II. The crude product was recrystallized from $CHCl_3$: Hexane (1:5) to obtain pure product **6a** (1.415 g, 4.95 mmol) as white needles in 99% yield [recrystallization via EtOAc : Hex gave 90% yield].

Spectral Data for 1,3-bromo-5,5-dimethylimidazolidine-2,4-dione (DBDMH): ^{1}H NMR (500 MHz, $CDCl_3$) δ 1.46 (s, 6H). ^{13}C NMR (126 MHz, $CDCl_3$) δ 172.18, 151.46, 68.89, 23.91. IR (cm $^{-1}$): 2995, 1777, 1718, 1456, 1434, 1334, 1200, 1117, 848, 714, 618. mp: 190-191 °C.[2] HRMS: TOF MS ES$^+$ ($C_5H_6Br_2N_2O_2$): Calc. [M + H]$^+$: 286.8854, Found [M + H]$^+$: 286.8859.

Scheme S11: Preparation of DBDPH (**6b**)

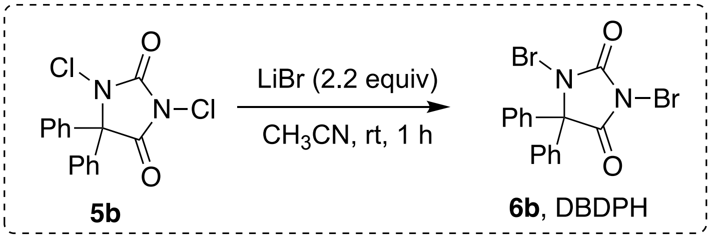

N-bromohydantoin **6b** was prepared from dichlorodiphenylhydantoin **5b** (1.61 g, 5.0 mmol) and LiBr (0.96 g, 11.0 mmol) using GP II. The crude product was recrystallized from $CHCl_3$: Hexane (1:5) to obtain pure product **6b** (2.0 g, 4.95 mmol) as white powdered solid in 99% yield [recrystallization via EtOAc : Hex gave 92% yield].

Spectral Data for 1,3-bromo-5,5-diphenylimidazolidine-2,4-dione (DBDPH): ^{1}H NMR (500 MHz, $CDCl_3$) δ 7.40 – 7.32 (m, 10H). ^{13}C NMR (126 MHz, $CDCl_3$) δ 173.23, 154.94, 138.79, 129.08, 128.98, 127.00. IR (cm $^{-1}$): 3197, 3065, 1772, 1707, 1448, 1399, 1193, 1016, 743, 694, 519. mp: 202-203 °C. HRMS: TOF MS ES$^+$ ($C_{15}H_{10}Br_2N_2O_2$): Calc. [M + H]$^+$: 410.9167, Found [M + H]$^+$: 410.9167.

Scheme S12: Preparation of DBH (**6c**)

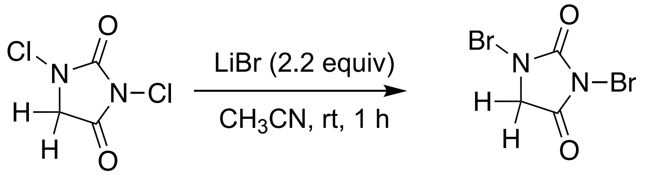

5c **6c**, DBH

N-bromohydantoin **6c** was prepared from dichlorohydantoin **5c** (844.8 mg, 5.0 mmol) and LiBr (0.96 g, 11.0 mmol) using GP II. The crude product was recrystallized from $CHCl_3$: Hexane (1:5) to obtain pure product **6c** (1.11 g, 4.3 mmol) as white solid in 86% yield [recrystallization via EtOAc : Hex gave 66% yield].

Spectral Data for 1,3-dibromoimidazolidine-2,4-dione (DBH): ^{1}H NMR (500 MHz, CD_3OD) δ 3.99 (s, 2H). ^{13}C NMR (126 MHz, CD_3OD) δ 175.87, 160.86, 48.75. IR (cm $^{-1}$): 3060, 2949, 1774, 1752, 1694, 1428, 1284, 1195, 1083, 989, 897, 781, 716, 631. mp:

134-135 °C. HRMS: TOF MS ES+ ($C_3H_2Br_2N_2O_2$): Calc. $[M + H]^+$: 258.8541, Found $[M + H]^+$: 258.8542.

Scheme S13: Preparation of NBS (**6d**)

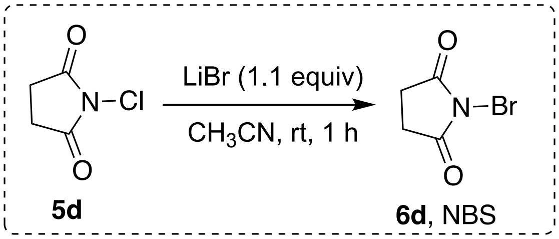

N-bromosuccinimide **6d** was prepared from N-chlorosuccinimide (NCS) **5d** (667.7 mg, 5.0 mmol) and LiBr (0.48 g, 5.5 mmol) using GP II. The crude product was recrystallized from $CHCl_3$: Hexane (1:5) to obtain pure product **6d** (845.4 mg, 4.75 mmol) as white crystalline solid in 95% yield [recrystallization via EtOAc : Hex gave 95% yield].

Spectral Data for 1-bromopyrrolidine-2,5-dione (NBS): ^1H NMR (500 MHz, $CDCl_3$) δ 2.96 (s, 4H). ^{13}C NMR (126 MHz, $CDCl_3$) δ 173.29, 28.82. IR (cm^{-1}): 2951, 1767, 1691, 1421, 1316, 1159, 812. mp: 177-178 °C.[3] HRMS: TOF MS ES+ ($C_4H_4BrNO_2$): Calc. $[M + H]^+$: 177.9503, Found $[M + H]^+$: 177.9504.

Scheme S14: Preparation of NBP (**6e**)

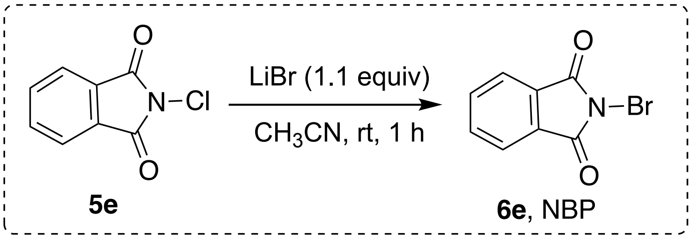

N-bromopthalimide **6e** was prepared from N-chloropthalimide (NCP) **5e** (907.9 mg, 5.0 mmol) and LiBr (0.48 g, 5.5 mmol) using GP II. The crude product was recrystallized from

$CHCl_3$: Hexane (1:5) to obtain pure product **6e** (1.08 g, 4.75 mmol) as white crystalline fluffy solid in 95% yield [recrystallization via EtOAc : Hex gave 95% yield].

Spectral Data for 2-bromoisoindoline-1,3-dione (NBP): ^1H NMR (500 MHz, $CDCl_3$) δ 7.88 (dd, *J* = 5.4, 3.1 Hz, 2H), 7.77 (dd, *J* = 5.4, 3.1 Hz, 2H). ^{13}C NMR (126 MHz, $CDCl_3$) δ 168.02, 134.50, 132.75, 123.78. IR (cm^{-1}): 3195, 3058, 1739, 1710, 1602, 1463, 1349, 1289, 1050, 859, 694. mp: 233-234 °C.[4] HRMS: TOF MS ES^+ ($C_8H_4BrNO_2$): Calc. $[M + H]^+$: 225.9503, Found $[M + H]^+$: 225.9505.

Scheme S15: Preparation of NBPyr (**6f**)

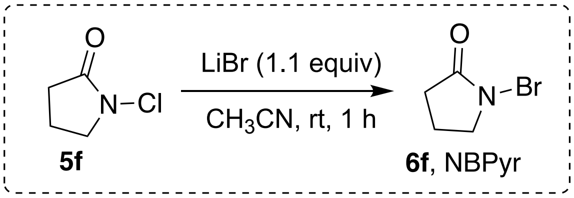

N-bromoamide **6f** was prepared from N-chloroamide **5f** (597.7 mg, 5.0 mmol) and LiBr (0.48 g, 5.5 mmol) using GP II. The crude product was recrystallized from $CHCl_3$: Hexane (1:5) to obtain pure product **6f** (721.6 mg, 4.4 mmol) as pale yellow solid in 88% yield [recrystallization via EtOAc : Hex gave 80% yield].

Spectral Data for 1-bromopyrrolidin-2-one (NBPyr): ^1H NMR (500 MHz, CD_3OD) δ 3.48 (t, *J* = 7.1 Hz, 2H), 2.42 (t, *J* = 8.1 Hz, 2H), 2.21 – 2.14 (m, 2H). ^{13}C NMR (126 MHz, CD_3OD) δ 182.04, 44.33, 31.19, 21.48. IR (cm^{-1}): 1691,1375, 784. mp: 87-98 °C. HRMS: TOF MS ES^+ (C_4H_6BrNO): Calc. $[M + H]^+$: 163.9711, Found $[M + H]^+$: 163.9713.

Scheme S16: Preparation of NBAc (**6g**)

CCDC No: 2225524

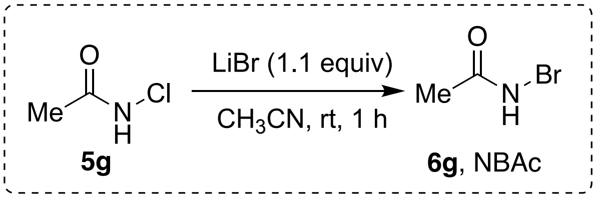

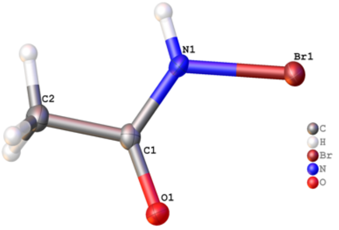

N-bromoamide **6g** was prepared from *N*-chloroacetamide **5g** (467.6 mg, 5.0 mmol) and LiBr (0.48 g, 5.5 mmol) using GP II. The crude product was recrystallized from $CHCl_3$: Hexane (1:5) to obtain pure product **6g** (683.1 mg, 4.95 mmol) as white crystalline fluffy solid in 99% yield [recrystallization via EtOAc : Hex gave 95% yield].

Spectral Data for *N*-bromoacetamide (NBAc): ^{1}H NMR (500 MHz, $CDCl_3$) δ 6.20 (s, 1H), 2.22 (s, 3H). ^{13}C NMR (126 MHz, $CDCl_3$) δ 170.20, 20.45. IR (cm $^{-1}$): 3385, 3143, 1609, 1423, 1365, 1257, 958, 768. mp: 105-106 °C. HRMS: TOF MS ES$^+$ (C_2H_4BrNO): Calc. [M + H]$^+$: 137.9554, Found [M + H]$^+$: 137.9559. Crystal structure: CCDC No: 2225524.

Scheme S17: Preparation of TBCA (**6h**)

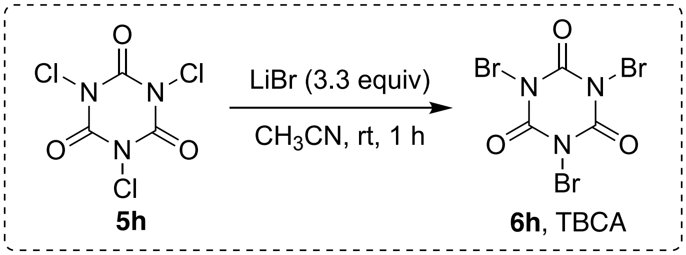

TBCA **6h** was prepared from TCCA **5h** (1.16 g, 5.0 mmol) and LiBr (1.44 g, 16.5 mmol) using GP II. The crude product was recrystallized from $CHCl_3$: Hexane (1:5) to obtain

pure product **6h** (1.5 g, 4.1 mmol) as white solid in 82% yield [recrystallization via EtOAc : Hex gave 67% yield].

Spectral Data for 1,3,5-tribromo-1,3,5-triazinane-2,4,6-trione (TBCA): ^{13}C NMR (126 MHz, CD_3OD) δ 151.58. IR (cm $^{-1}$): 1740, 1420, 1339, 717. mp: > 300 °C. HRMS: TOF MS ES$^+$ ($C_3Br_3N_3O_3$): Calc. [M + H]$^+$: 365.7547, Found [M + H]$^+$: 365.7549.

Scheme S18: Preparation of NBSac (**6i**)

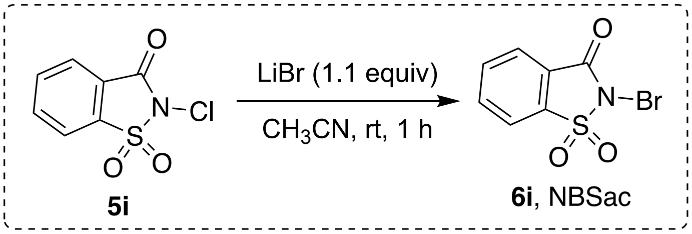

CCDC no: 2225526

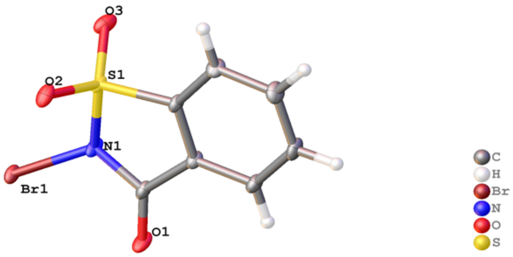

N-bromosaccharin **6i** was prepared from *N*-chloroderivative **5i** (1.09 g, 5.0 mmol) and LiBr (0.48 g, 5.5 mmol) using GP II. The crude product was recrystallized from $CHCl_3$: Hexane (1:5) to obtain pure product **6i** (1.28 g, 4.9 mmol) as white crystalline plate like solid in 98% yield [recrystallization via EtOAc : Hex gave 98% yield].

Spectral Data for 2-bromobenzo[*d*]isothiazol-3(2*H*)-one 1,1-dioxide (NBSac): ^{1}H NMR (500 MHz, $CDCl_3$) δ 8.10 (d, *J* = 7.5 Hz, 1H), 7.97 (d, *J* = 7.6 Hz, 1H), 7.91 (td, *J* = 7.6, 1.2 Hz, 1H), 7.86 (td, *J* = 7.5, 1.1 Hz, 1H). ^{13}C NMR (126 MHz, $CDCl_3$) δ 157.93, 138.49, 135.24, 134.79, 127.16, 125.88, 121.84, 121.87. IR (cm $^{-1}$): 3096, 1703, 1461, 1349, 1231, 1184, 942, 745, 571. mp: 179-180 °C. HRMS: TOF MS ES$^+$ ($C_7H_4BrNO_3S$): Calc. [M + H]$^+$: 261.9173, Found [M + H]$^+$: 261.9178. Crystal Structure Data: CCDC No: 2225526.

Scheme S19: Preparation of DiB-T (**6j**)

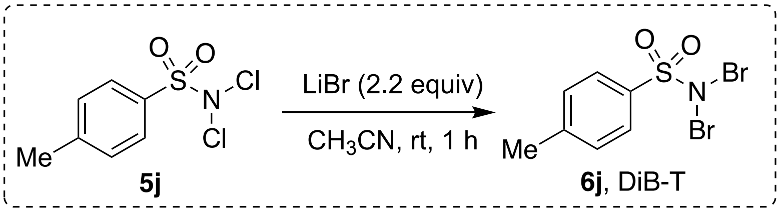

Dibromoamine-T **6j** was prepared from Dichloroamine-T **5j** (1.2 g, 5.0 mmol) and LiBr (0.48 g, 5.5 mmol) using GP II. The crude product was recrystallized from $CHCl_3$: Hexane (1:5) to obtain pure product **6j** (1.56 g, 4.75 mmol) as white thin crystalline solid in 95% yield [recrystallization via EtOAc : Hex gave 90% yield].

Spectral Data for Tosylbromoimidous bromide (DiB-T): ^{1}H NMR (500 MHz, $CDCl_3$) δ 7.81 (d, J = 7.8 Hz, 2H), 7.31 (d, J = 7.8 Hz, 2H), 2.44 (s, 3H). ^{13}C NMR (126 MHz, $CDCl_3$) δ 143.79, 139.14, 129.88, 126.59, 21.68. IR (cm $^{-1}$): 3351, 3257, 3063, 3047, 1595, 1530, 1499, 1307, 1155, 1096, 909, 812, 535. mp: 116-118 °C. HRMS: TOF MS ES^+ ($C_7H_7Br_2NO_2S$): Calc. $[M + H]^+$: 329.8622, Found $[M + H]^+$: 329.8627.

General Procedure for the Synthesis of *N*-iodinating Agents (GP III):

In a clean dry 100 mL round bottom LiI (3.35 g, 25.0 mmol) was introduced in a mixture of toluene (15 mL) and acetonitrile (15 mL) as solvent and stirred for 10 minutes. General substrate 5 (5.0 mmol) was then added slowly over a couple of minutes and stirred vigorously at room temperature for an hour (once the addition is complete, within 5-10 minutes, there is a formation of a precipitate). The reaction was quenched by the addition of water (5 mL) and further diluted by adding ethyl acetate (5 mL). The organic layer was separated, and the aqueous layer was extracted with ethyl acetate (6 X 5 mL) till the aqueous layer became colorless. The combined organics were dried over anhyd. Na_2SO_4 and concentrated under reduced pressure. The solids were dissolved in a minimum amount of hot ethyl acetate (or dioxane) and hexane (five times the volume of dissolving solvent) was layered on top and cooled at –20° C. The crystals were collected by suction filtration and dried under high vacuum for an hour. These solids were used for recording analytical data.

Analytical Data for the N-Iodo halogenating agents:

Scheme S20: Preparation of DIDMH (**7a**)

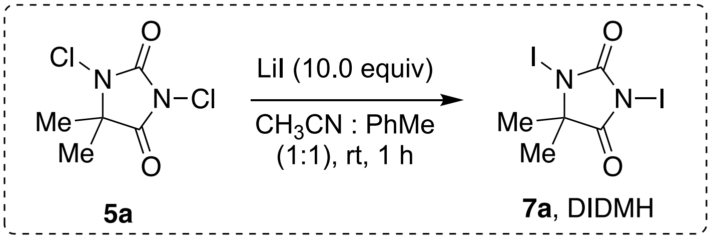

N-iodohydantoin **7a** was prepared from dichlorodimethylhydantoin **5a** (0.98 g, 5.0 mmol) and LiI (6.7 g, 50.0 mmol) using GP II. The crude product was recrystallized from hot

EtOAc : Hexane (1 : 5) to obtain pure product **7a** (1.54 g, 4.05 mmol) as brownish white solid in 81% yield. [Note: hot dioxane can also be used for recrystallisation but the yield is not good, and the mother liquor had to be recrystallized thrice to get comparable yields. Also, the solids were dark brown in color.]

Spectral Data for 1,3-iodo-5,5-dimethylimidazolidine-2,4-dione (DIDMH): ^{1}H NMR (500 MHz, CD_3OD) δ 1.41 (s, 6H). ^{13}C NMR (126 MHz, CD_3OD) δ 181.50, 158.38, 61.09, 24.89. IR: (cm $^{-1}$): 2976, 1665, 1340, 1267, 1211, 1038, 759, 607. Mp: 200-201 °C. HRMS: TOF MS ES^{+} ($C_5H_6I_2N_2O_2$): Calc. [M + H]$^{+}$: 380.8597, Found [M + H]$^{+}$: 380.8599.

Scheme S21: Preparation of DIDPH (**7b**)

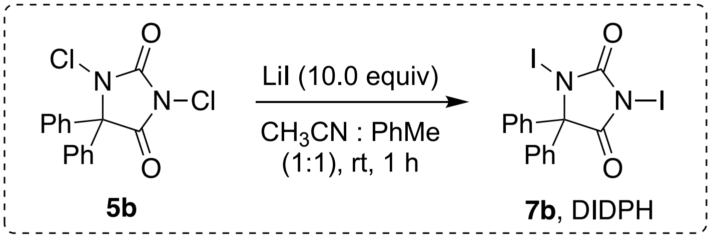

N-iodohydantoin **7b** was prepared from dichlorodiphenylhydantoin **5b** (1.61 g, 5.0 mmol) and LiI (6.7 g, 50.0 mmol) using GP II. The crude product was recrystallized from hot EtOAc : Hexane (1 : 5) to obtain pure product **7b** (2.27 g, 4.5 mmol) as brownish yellow solid in 90% yield. [Note: hot dioxane can also be used for recrystallisation but the yield is not good, and the mother liquor had to be recrystallized thrice to get comparable yields. Also, the solids were dark brown in color.]

Spectral Data for 1,3-iodo-5,5-diphenylimidazolidine-2,4-dione (DIDPH): ^{1}H NMR (500 MHz, CD_3OD) δ 7.40 – 7.36 (m, 4H), 7.36 – 7.32 (m, 4H), 7.32 – 7.28 (m, 2H). ^{13}C

NMR (126 MHz, CD_3OD) δ 183.72, 166.16, 142.17, 129.35, 128.86, 128.24. IR (cm^{-1}): 3075, 1682, 1572, 1444, 1380, 1281, 694. mp: 308-309 °C. HRMS: TOF MS ES^+ ($C_{15}H_{10}I_2N_2O_2$): Calc. $[M + H]^+$: 504.8910, Found $[M + H]^+$: 504.8915.

Scheme S22: Preparation of DIH (**7c**)

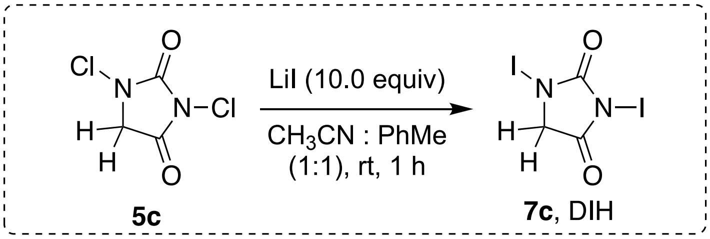

N-iodohydantoin **7c** was prepared from dichlorohydantoin **5c** (844.8 mg, 5.0 mmol) and LiI (6.7 g, 50.0 mmol) using GP II. The crude product was recrystallized from hot EtOAc : Hexane (1 : 5) to obtain pure product **7c** (1.46 g, 4.15 mmol) as yellowish solid in 83% yield. [Note: Care must be taken as the recrystallized products are very fine solids and makes it very difficult to handle].

Spectral Data for 1,3-diiodoimidazolidine-2,4-dione (DIH): ^{1}H NMR (500 MHz, $CDCl_3$) δ 4.32 (s, 2H). ^{13}C NMR (126 MHz, $CDCl_3$) δ 162.78, 152.67, 56.70. IR (cm^{-1}): 1747, 1694, 1595. mp: 74-76 °C. HRMS: TOF MS ES^+ ($C_3H_2I_2N_2O_2$): Calc. $[M + H]^+$: 352.8284, Found $[M + H]^+$: 352.8288.

Scheme S23: Preparation of NIS (**7d**)

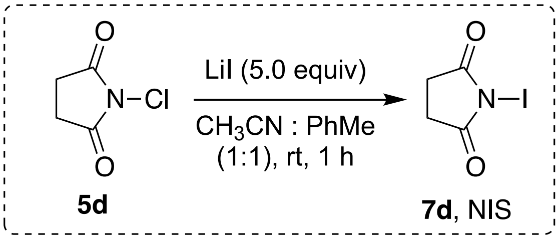

N-iodosuccinimide **7d** was prepared from N-chlorosuccinimide (NCS) **5d** (667.7 mg, 5.0 mmol) and LiI (3.35 g, 25.0 mmol) using GP II. The crude product was recrystallized from hot dioxane : Hexane (1:5) to obtain pure product **7d** (1.07 g, 4.75 mmol) as white crystalline solid in 95% yield [recrystallization via EtOAc : Hex gave 80% yield].

Spectral Data for 1-iodopyrrolidine-2,5-dione (NIS): ^{1}H NMR (500 MHz, $CDCl_3$) δ 2.91 (s, 4H). ^{13}C NMR (126 MHz, $CDCl_3$) δ 171.28, 28.01. IR (cm $^{-1}$): 1743, 1624, 1325, 1177, 812, 642, 566. mp: 198-199 °C.[5] HRMS: TOF MS ES$^+$ ($C_4H_4INO_2$): Calc. [M + H]$^+$: 225.9365, Found [M + H]$^+$: 225.9366.

Scheme S24: Preparation of NIP (**7e**)

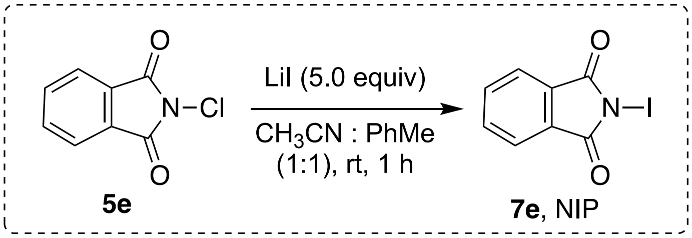

N-bromopthalimide **7e** was prepared from N-chloropthalimide (NCP) **5e** (907.9 mg, 5.0 mmol) and LiI (3.35 g, 25.0 mmol) using GP II. The crude product was recrystallized from hot dioxane : Hexane (1 : 5) to obtain pure product **7e** (1.08 g, 4.75 mmol) as long sharp white crystalline solid in 97% yield [recrystallization via EtOAc : Hex gave 95% yield].

Spectral Data for 2-iodoisoindoline-1,3-dione (NIP): ^{1}H NMR (500 MHz, $CDCl_3$) δ 7.88 (dd, *J* = 5.5, 3.1 Hz, 2H), 7.77 (dd, *J* = 5.5, 3.1 Hz, 2H). ^{13}C NMR (126 MHz, $CDCl_3$) δ 167.99, 137.56, 134.50, 132.75, 123.78. IR (cm^{-1}): 3060, 1716, 1602, 1466, 1374, 1298, 1040, 710, 642. mp: 231-232 °C.[6] HRMS: TOF MS ES^+ ($C_8H_4INO_2$): Calc. $[M + H]^+$: 273.9365, Found $[M + H]^+$: 273.9365.

Scheme S25: Preparation of NIPyr (**7f**)

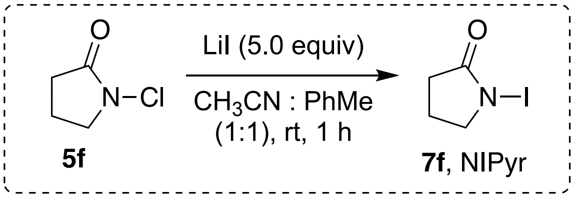

N-bromoamide **7f** was prepared from N-chloroamide **5f** (597.7 mg, 5.0 mmol) and LiI (3.35 g, 25.0 mmol) using GP II. The crude product was recrystallized from hot dioxane : Hexane (1 : 5) to obtain pure product **7f** (812.6 mg, 3.85 mmol) as dark brown solid in 77% yield.

Spectral Data for 1-iodopyrrolidin-2-one (NIPyr): ^{1}H NMR (500 MHz, CD_3OD) δ 3.44 (t, *J* = 7.0 Hz, 2H), 2.32 (t, *J* = 7.0 Hz, 2H), 2.21 – 2.10 (m, 2H). ^{13}C NMR (126 MHz, CD_3OD) δ 181.30, 43.55, 31.11, 21.48. IR (cm^{-1}): 1680, 1325, 717. mp: 115-125 °C. HRMS: TOF MS ES^+ (C_4H_6INO): Calc. $[M + H]^+$: 211.9572, Found $[M + H]^+$: 211.9575.

Scheme S26: Preparation of NIAc (**7g**)

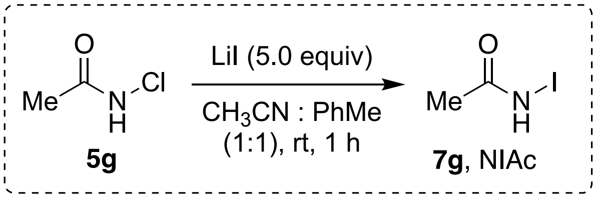

N-iodoamide **7g** was prepared from *N*-chloroacetamide **5g** (467.6 mg, 5.0 mmol) and LiI (3.35 g, 25.0 mmol) using GP II. The crude product was recrystallized from hot dioxane : Hexane (1 : 5) to obtain pure product **7g** (850.8 mg, 4.6 mmol) as white solid in 92% yield [recrystallization via EtOAc : Hex gave 88% yield].

Spectral Data for *N*-iodoacetamide (NIAc): ^{1}H NMR (500 MHz, $CDCl_3$) δ 4.60 (s, 1H), 1.95 (s, 3H). ^{13}C NMR (126 MHz, $CDCl_3$) δ 176.43, 22.14. IR (cm $^{-1}$): 3403, 2922, 2864, 1649, 1624, 1291, 1105, 866, 616. mp: 57-60 °C (decomp). HRMS: TOF MS ES$^+$ (C_2H_4INO): Calc. [M + H]$^+$: 185.9416, Found [M + H]$^+$: 185.9411.

Scheme S27: Preparation of TICA (**7h**)

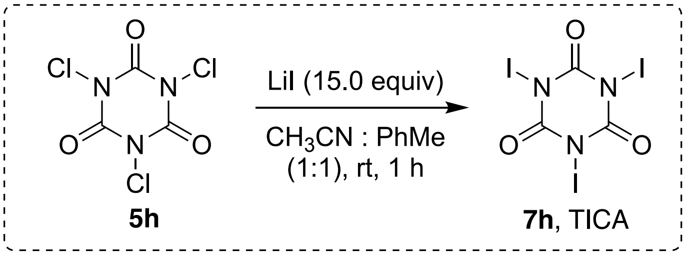

TICA **7h** was prepared from TCCA **5h** (1.16 g, 5.0 mmol) and LiI (10.05 g, 75.0 mmol) using GP II. The crude product was recrystallized from hot EtOAc : Hexane (1 : 5) to obtain pure product **7h** (1.98 g, 4.1 mmol) as yellowish brown solid in 78% yield. [Note:

Excess solvent was used Acetonitrile (50 mL) and Toluene (50 mL) during this reaction to completely dissolve the reaction mixture. Also, during recrystallisation 5 drops of DMSO was used in addition to hot EtOAc].

Spectral Data for 1,3,5-triiodo-1,3,5-triazinane-2,4,6-trione (TICA): ^{13}C NMR (126 MHz, *d*6-DMSO) δ 149.89. IR (cm $^{-1}$):1710, 1665, 1315, 732. mp: > 300 °C. HRMS: TOF MS ES$^+$ ($C_3I_3N_3O_3$): Calc. [M + H]$^+$: 507.7152, Found [M + H]$^+$: 507.7155.

Scheme S28: Preparation of NISac (**7i**)

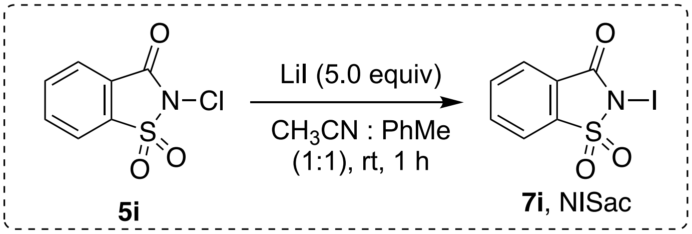

N-iodosaccharin **7i** was prepared from *N*-chloroderivative **5i** (1.09 g, 5.0 mmol) and LiI (3.35 g, 25.0 mmol) using GP II. The crude product was recrystallized from hot EtOAc : Hexane (1 : 5) to obtain pure product **7i** (1.42 g, 4.6 mmol) as white thin needle like crystalline solid in 92% yield.

Spectral Data for 2-Iodobenzo[*d*]isothiazol-3(2*H*)-one-1,1-dioxide (NISac): ^{1}H NMR (500 MHz, CD_3OD) δ 7.81 – 7.78 (m, 1H), 7.77 – 7.75 (m, 1H), 7.70 – 7.67 (m, 2H). ^{13}C NMR (126 MHz, CD_3OD) δ 170.85, 144.15, 133.61, 132.30, 131.87, 122.97, 119.50. IR (cm $^{-1}$): 3249, 1642, 1589, 1351, 1244, 1134, 971, 949, 747, 672. mp: 204-205 °C. HRMS: TOF MS ES$^+$ ($C_7H_4INO_3S$): Calc. [M + H]$^+$: 309.9035, Found [M + H]$^+$: 309.9039.

Scheme S29: Preparation of DiI-T (**7j**)

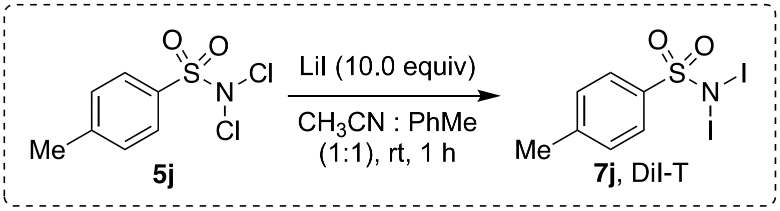

Diiodamine-T **7j** was prepared from Dichloroamine-T **5j** (1.2 g, 5.0 mmol) and LiI (6.7 g, 50.0 mmol) using GP II. The crude product was recrystallized from hot EtOAc : Hexane (1 : 5) to obtain pure product **7j** (1.95 g, 4.6 mmol) as yellowish brown solid in 92% yield [Acetonitrile (30 mL) and toluene (30 mL) was used as solvents for this reaction].

Spectral Data for Tosyliodoimidous chloride (DiI-T): ^{1}H NMR (500 MHz, CD_3OD) δ 7.77 (d, $J = 8.6$ Hz, 2H), 7.33 (d, $J = 8.4$ Hz, 2H), 2.41 (s, 3H). ^{13}C NMR (126 MHz, CD_3OD) δ 143.67, 142.32, 130.34, 127.01, 21.41. IR (cm $^{-1}$): 1588, 1524, 1295, 1152, 913, 806, 665. mp: 94-96 °C. HRMS: TOF MS ES^{+} ($C_7H_7I_2NO_2S$): Calc. $[M + H]^{+}$: 423.8365, Found $[M + H]^{+}$: 423.8369.

Mechanistic Experiments:

Scheme S30: Failed synthesis of NCS from NBS/NIS (reverse reaction)

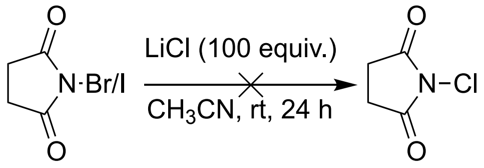

In a clean dry 100 mL round bottom was introduced LiCl (1.1 equiv, 42.4 mg) in acetonitrile (6 mL) as solvent. The round bottom was covered with Al-foil and the solids were stirred for 5 minutes till it became completely soluble in the acetonitrile. NBS/NIS (1.0 mmol) was then slowly added over a couple of minutes and stirred vigorously at room temperature for 24 h (the reaction was homogeneous, and no precipitate was observed). The reaction was quenched by the addition of water (2 mL) and further diluted by adding ethyl acetate (2 mL). The organic layer was separated, and the aqueous layer was extracted with ethyl acetate (3 X 1 mL). The combined organics were dried over anhyd. Na_2SO_4 and concentrated under reduced pressure. NBS/NIS was recovered completely. Under the exact similar reaction condition, when the amount of LiCl (100.0 equiv, 4.23 g) was increased; NBS/NIS was again recovered from this heterogeneous reaction mixture.

Scheme S31: Synthesis of NIS (**7d**) from NBS (**6d**)

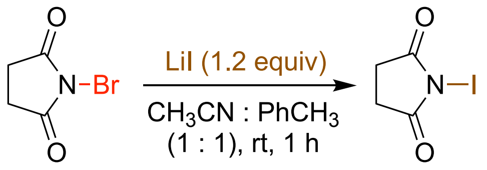

6d **7d** , 72%

In a clean dry 100 mL round bottom LiI (803 mg, 6.0 mmol) was introduced in a mixture of toluene (15 mL) and acetonitrile (15 mL) as solvent and stirred for 10 minutes. NBS,

6d (884 mg, 5.0 mmol) was then added slowly over a couple of minutes and stirred vigorously at room temperature for an hour (once the addition is complete, within 5-10 minutes, there is a formation of a precipitate). The reaction was quenched by the addition of water (10 mL) and further diluted by adding ethyl acetate (10 mL). The organic layer was separated, and the aqueous layer was extracted with ethyl acetate (6 X 5 mL) till the aqueous layer became colorless. The combined organics were dried over anhyd. Na_2SO_4 and concentrated under reduced pressure. The solids were dissolved in a minimum amount of hot or dioxane and hexane (five times the volume of dissolving solvent) was layered on top and cooled at –20° C. The crystals were collected by suction filtration and dried under high vacuum for an hour to generate pure off-white crystals of NIS (810 mg, 3.6 mmol).

Scheme S32: Synthesis of 1-(1-chloro-2-bromoethyl)-4-methoxybenzene (**8**)

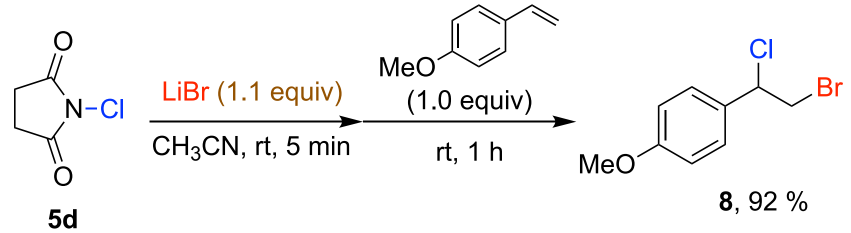

5d **8**, 92 %

In a clean dry 10 mL round bottom was introduced LiBr (95.5 mg, 1.1 mmol) in acetonitrile (2 mL) as solvent. The round bottom was covered with Al-foil and the solids were stirred for 5 minutes till it became completely soluble in the acetonitrile. NCS (133.5 mg, 1.0 mmol) was added in one portion and the mixture stirred for another five minutes. 4-Methoxystyrene (0.14 mL, 1.0 mmol) was added, and the contents were stirred for one hour. The reaction was quenched by adding water (2 mL) and the organic layer was

isolated. The aqueous layer was extracted (3 X 2 mL) with EtOAc and the combined organics were dried over Na_2SO_4 and concentrated under reduced pressure. The crude product was further purified by column chromatography (2 % EA-hex).

Spectral Data for compound 8: ^{1}H NMR (500 MHz, $CDCl_3$) δ 7.31 (d, *J* = 8.6 Hz, 2H), 6.91 (d, *J* = 8.7 Hz, 2H), 4.88 (dd, *J* = 9.1, 3.2 Hz, 1H), 3.81 (s, 3H), 3.60 (dd, *J* = 10.4, 3.4 Hz, 1H), 3.53 (dd, *J* = 10.4, 9.1 Hz, 1H). ^{13}C NMR (126 MHz, $CDCl_3$) δ 159.81, 132.50, 127.37, 114.20, 73.61, 55.45, 40.47.[7-8]

Scheme S33: Synthesis of 1-(1-chloro-2-iodoethyl)-4-methoxybenzene (**9**)

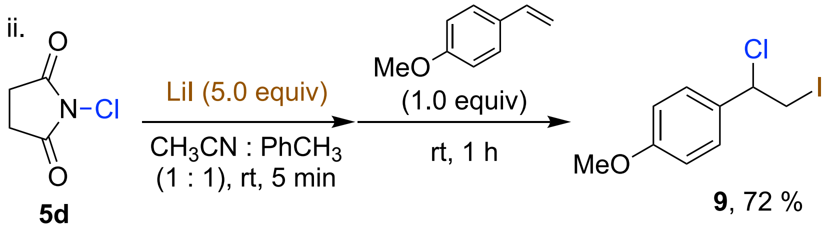

5d **9**, 72 %

Compound **9** was synthesized using GP IV, to yield a colorless oil as the final product in 72% isolated yield (214.2 mg, 0.72 mmol).

Spectral Data for compound 9: ^{1}H NMR (500 MHz, $CDCl_3$) δ 7.31 (d, *J* = 8.7 Hz, 2H), 6.91 (d, *J* = 8.7 Hz, 2H), 5.07 (dd, *J* = 9.9, 5.6 Hz, 1H), 3.84 – 3.79 (m, 1 H), 3.82 (s, 3H), 3.72 (dd, *J* = 10.0, 9.4 Hz, 1H). ^{13}C NMR (126 MHz, $CDCl_3$) δ 160.20, 131.30, 128.62, 114.24, 61.73, 55.47, 10.43.[7-8]

Scheme S34: Synthesis of (2-bromo-1-chloroethyl)benzene (**10**)

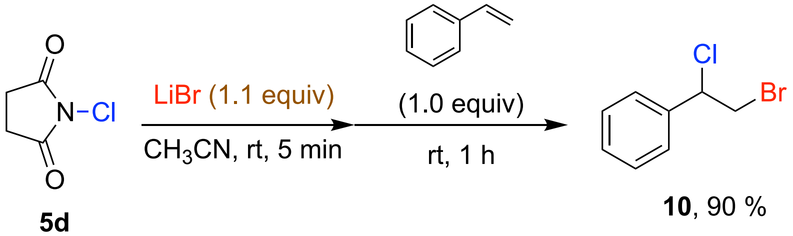

5d **10**, 90 %

Compound **10** was synthesized using GP IV, to yield a colorless oil as the final product in 90% isolated yield (197.1 mg, 0.90 mmol).

Spectral Data for compound 9: ^{1}H NMR (500 MHz, $CDCl_3$) δ 7.47 – 7.32 (m, 5H), 5.15 (dd, *J* = 10.7, 5.4 Hz, 1H), 4.08 (dd, *J* = 10.3, 5.4 Hz, 1H), 4.03 (dd, *J* = 10.5, 6.8 Hz,1H). ^{13}C NMR (126 MHz, $CDCl_3$) δ 138.74, 129.34, 129.01, 127.80, 50.99, 35.15.[7-8]

Scheme S35: Synthesis of (1-chloro-2-iodoethyl)benzene (**11**)

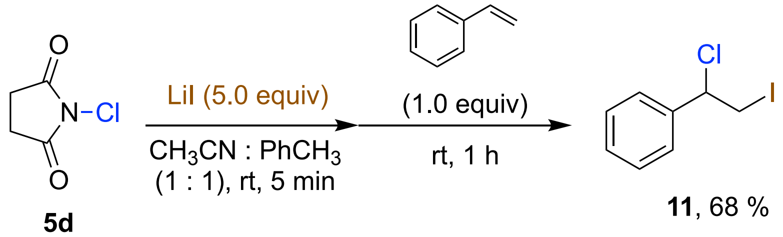

5d **11**, 68 %

Compound **11** was synthesized using GP IV, to yield a colorless oil as the final product in 68% isolated yield (181 mg, 0.679 mmol).

Spectral Data for compound 11: ^{1}H NMR (500 MHz, $CDCl_3$) ^{1}H NMR (500 MHz, Chloroform-*d*) δ 7.44 – 7.31 (m, 5H), 5.07 (dd, *J* = 9.7, 5.8 Hz, 1H), 3.81 (dd, *J* = 10.1, 5.7 Hz, 1H), 3.73 (dd, *J* = 9.9, 5.8 Hz, 1H). ^{13}C NMR (126 MHz, $CDCl_3$) δ 139.22, 129.30, 128.93, 127.33, 77.41, 77.16, 76.91, 61.78, 10.09.[7-8]

Crystal Structures:

1. Crystal structure data for **5g:**

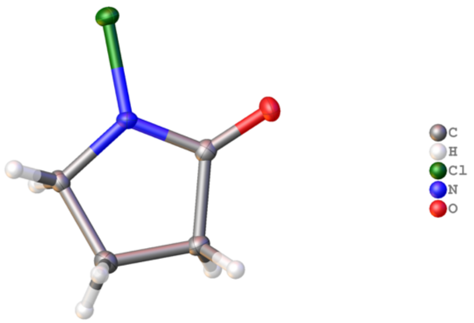

Experimental. Single colorless cube-shaped crystals of **5g** used as received. A suitable crystal with dimensions 0.16 × 0.13 × 0.10 mm^3 was selected and mounted on a nylon loop with paratone oil on a XtaLAB Synergy, Dualflex, HyPix diffractometer. The crystal was kept at a steady T = 100.00(10) K during data collection. The structure was solved with the **ShelXT** (Sheldrick, 2015) solution program using dual methods and by using **Olex2** 1.5 (Dolomanov et al., 2009) as the graphical interface. The model was refined with **ShelXL** 2018/3 (Sheldrick, 2015) using full matrix least squares minimisation on $\boldsymbol{F^2}$.

Crystal Data. C_4H_6ClNO, M_r = 119.55, orthorhombic, $P2_12_12_1$ (No. 19), a = 6.54180(10) Å, b = 6.94990(10) Å, c = 11.24660(10) Å, $\alpha = \beta = \gamma =$ 90°, V = 511.325(12) Å^3, T = 100.00(10) K, Z = 4, Z' = 1, m(Cu K_a) = 5.539, 5414 reflections measured, 1090 unique (R_{int} = 0.0294) which were used in all calculations. The final wR_2 was 0.0482 (all data) and R_1 was 0.0190 (I≥2 σ(I)).

Structure Quality Indicators

d min (Cu\a) 2Θ=159.5°	0.78	I/σ(I)	49.9	Rint	2.94%	Full 135.4° 98% to 159.5°	100		
Shift	0.000	Max Peak	0.2	Min Peak	-0.2	GooF	1.083	Hooft	-.023(8)

Formula	C_4H_6ClNO	Waveleng	1.54184
**CCDC**	**2225525**	Radiation	Cu K_α
$D_{calc.}$/ g cm^{-3}	1.553	Θ_{min}/	7.492
μ/mm^{-1}	5.539	Θ_{max}/	79.757
Formula Weight	119.55	Measured	5414
Color	colourless	Indep't F	1090
Shape	cube-shaped	Refl's I≥2	1083
Size/mm^3	0.16×0.13×0.1(	R_{int}	0.0294
T/K	100.00(10)	Parame	65
Crystal System	orthorhombic	Restrai	0
Flack Parameter	-0.018(9)	Largest I	0.222
Hooft Parameter	-0.023(8)	Deepest	-0.203
Space Group	$P2_12_12_1$	GooI	1.083
a/Å	6.54180(10)	wR_2 (all c	0.0482
b/Å	6.94990(10)	wR_2	0.0481
c/Å	11.24660(10)	R_1 (all d	0.0192
α/°	90	R_1	0.0190
β/°	90		
γ/°	90		
V/Å^3	511.325(12)		
Z	4		
Z'	1		

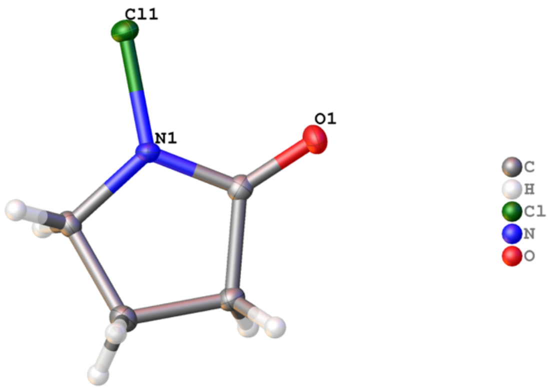

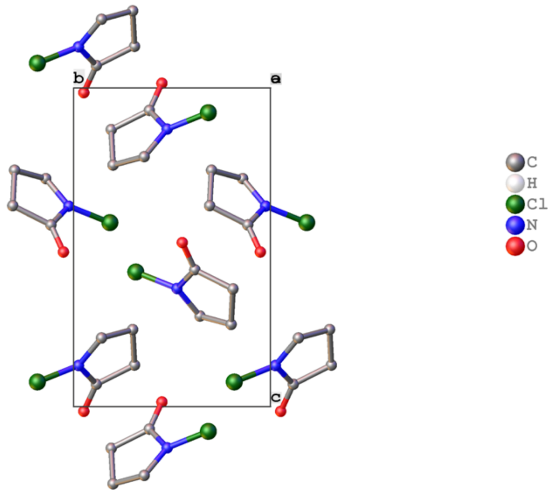

2. Crystal structure data for **6f:**

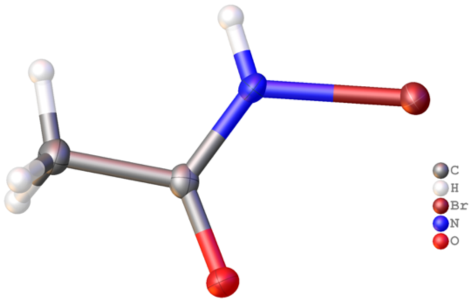

Experimental. Single colorless needle-shaped crystals of **6f** used as received. A suitable crystal with dimensions 0.28 × 0.09 × 0.05 mm^3 was selected and mounted on a nylon loop with paratone oil on a XtaLAB Synergy, Dualflex, HyPix diffractometer. The crystal was kept at a steady T = 100.01(10) K during data collection. The structure was solved with the **ShelXT** (Sheldrick, 2015) solution program using dual methods and by using **Olex2** 1.5 (Dolomanov et al., 2009) as the graphical interface. The model was refined with **ShelXL** 2018/3 (Sheldrick, 2015) using full matrix least squares minimisation on F^2.

Crystal Data. C_2H_4BrNO, M_r = 137.97, tetragonal, $P4_12_12$ (No. 92), a = 7.45037(6) Å, b = 7.45037(6) Å, c = 16.1486(2) Å, $\alpha = \beta = \gamma =$ 90°, V = 896.376(18) Å^3, T = 100.01(10) K, Z = 8, Z' = 1, m(Cu K_a) = 11.120, 6156 reflections measured, 971 unique (R_{int} = 0.0342) which were used in all calculations. The final wR_2 was 0.0549 (all data) and R_1 was 0.0193 (I≥2 σ(I)).

Structure Quality Indicators

**Reflections:**	d min (Cu\a) 2Θ=160.5° 0.78	I/σ(I) 51.5	Rint 3.42%	Full 135.4° 99% to 160.5° 100	
**Refinement:**	Shift 0.000	Max Peak 0.3	Min Peak -0.5	GooF 1.141	Hooft -.01(2)

Formula	C_2H_4BrNO	Wavelength/Å	1.54184
**CCDC**	**2225524**	Radiation type	Cu K_α
$D_{calc.}$/ g cm^{-3}	2.045	Θ_{min}/°	6.543
μ/mm^{-1}	11.120	Θ_{max}/°	80.251
Formula Weight	137.97	Measured Refl's.	6156
Color	colourless	Indep't Refl's	971
Shape	needle-shaped	Refl's I≥2 σ(I)	967
Size/mm^3	0.28×0.09×0.05	R_{int}	0.0342
T/K	100.01(10)	Parameters	51
Crystal System	tetragonal	Restraints	0
Flack Parameter	-0.02(2)	Largest Peak	0.340
Hooft Parameter	-0.01(2)	Deepest Hole	-0.486
Space Group	$P4_12_12$	GooF	1.141
a/Å	7.45037(6)	wR_2 (all data)	0.0549
b/Å	7.45037(6)	wR_2	0.0547
c/Å	16.1486(2)	R_1 (all data)	0.0195
α/°	90	R_1	0.0193
β/°	90		
γ/°	90		
V/$Å^3$	896.376(18)		
Z	8		
Z'	1		

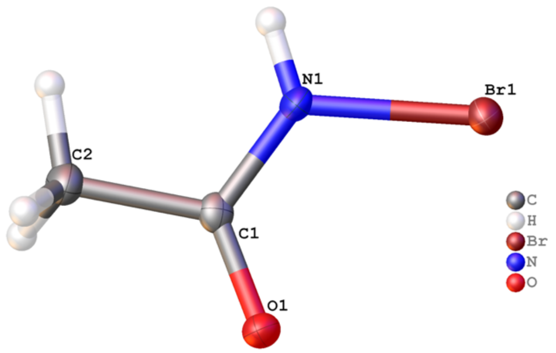

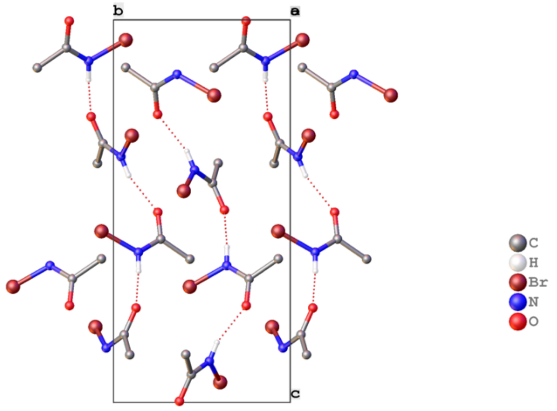

3. Crystal structure data for **6i**:

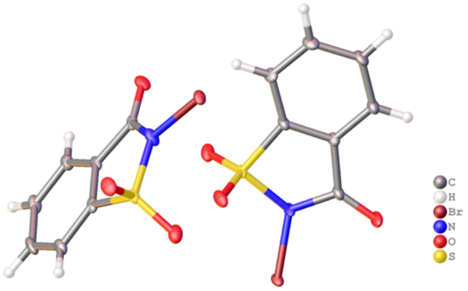

Experimental. Single colourless plate-shaped crystals of **6i** used as received. A suitable crystal with dimensions 0.24 × 0.14 × 0.09 mm^3 was selected and mounted on a nylon loop with paratone oil on a XtaLAB Synergy, Dualflex, HyPix diffractometer. The crystal was kept at a steady T = 99.98(11) K during data collection. The structure was solved with the **ShelXT** 2018/2 (Sheldrick, 2018) solution program using dual methods and by using **O.** V. Dolomanov, L. J. Bourhis, R. J. Gildea, J. A. K. Howard and H. Puschmann,Olex2: a complete structure solution, refinement and analysis program.J. Appl. Cryst. (2009). 42, 339-341. as the graphical interface. The model was refined with **ShelXL** 2018/3 (Sheldrick, 2015) using full matrix least squares minimisation on $\boldsymbol{F}^2$.

Crystal Data. $C_7H_4BrNO_3S$, M_r = 262.08, monoclinic, $P2_1/c$ (No. 14), a = 7.69833(8) Å, b = 28.22391(17) Å, c = 8.62702(9) Å, β = 116.4821(13)°, $\alpha = \gamma$ = 90°, V = 1677.77(3) $Å^3$, T = 99.98(11) K, Z = 8, Z' = 2, m(Cu K_a) = 8.815, 20927 reflections measured, 3397 unique (R_{int} = 0.0342) which were used in all calculations. The final wR_2 was 0.0659 (all data) and R_1 was 0.0249 (I≥2 σ(I)).

Structure Quality Indicators

**Reflections:**	d min (Cu\a) 2Θ=160.4° 0.78	I/σ(I) 46.4	Rint 3.42%	Full 135.4° 99% to 160.4° 100
**Refinement:**	Shift -0.004	Max Peak 0.5	Min Peak -0.3	GooF 1.086

Formula	$C_7H_4BrNO_3S$	Wavelength/Å	1.54184
**CCDC**	**2225526**	Radiation type	Cu K_α
$D_{calc.}$/ g cm^{-3}	2.075	Θ_{min}/°	3.132
μ/mm^{-1}	8.815	Θ_{max}/°	87.759
Formula Weight	262.08	Measured Refl's.	20927
Color	colourless	Indep't Refl's	3397
Shape	plate-shaped	Refl's I≥2 σ(I)	3320
Size/mm^3	0.24×0.14×0.09	R_{int}	0.0342
T/K	99.98(11)	Parameters	236
Crystal System	monoclinic	Restraints	0
Space Group	$P2_1/c$	Largest Peak	0.469
a/Å	7.69833(8)	Deepest Hole	-0.343
b/Å	28.22391(17)	GooF	1.086
c/Å	8.62702(9)	wR_2 (all data)	0.0659
α/°	90	wR_2	0.0654
β/°	116.4821(13)	R_1 (all data)	0.0254
γ/°	90	R_1	0.0249
V/Å^3	1677.77(3)		
Z	8		
Z'	2		

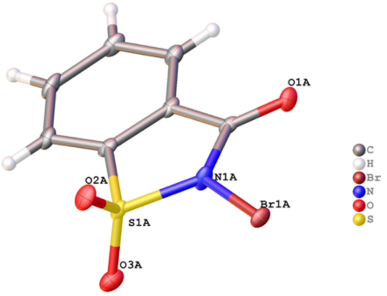

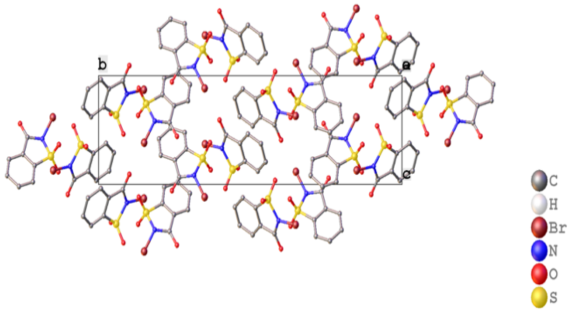

References

1. Whitehead, D. C.; Staples, R. J.; Borhan, B., A simple and expedient method for the preparation of N-chlorohydantoins. *Tetrahedron Lett.* **2009**, *50*, 656-658.
2. Huang, H.; Pan, H.; Cai, Y.; Liu, M.; Tian, H.; Shi, Y., Enantioselective 6-endo bromoaminocyclization of 2,4-dienyl N-tosylcarbamates catalyzed by a chiral phosphine oxide-Sc(OTf) ₃ complex. A dramatic additive effect. *Org. Biomol. Chem.* **2015**, *13*, 3566-3570.
3. Bhattacharya, A.; mani Shukla, P.; Maji, B., “Haliranium Ion”-Induced Intermolecular Friedel-Crafts Alkylation in HFIP: Synthesis of β,β-Diaryl α-Halo carbonyl Compounds. *Eur. J. Org. Chem.* **2021**, *2021*, 4737-4749.
4. Kim, J. J.; Kweon, D. H.; Cho, S.; Kim, H. K.; Lee, S. G.; Yoon, Y.-J., Conversion of Nucleophilic Halides to Electrophilic Halides: Efficient and Selective Halogenation of Azinones, Amides, and Carbonyl Compounds Using Metal Halide/Lead Tetraacetate. *Synlett* **2006**, *2006*, 194-200.
5. Beebe, T. R.; Wolfe, J. W., N-bromination of amides, imides, and sulfonamides with acetyl hypobromite. *J. Org. Chem.* **1970**, *35*, 2056-2057.
6. Kim, H.; Kim, T.; Lee, D. G.; Roh, S. W.; Lee, C., Nitrogen-centered radical-mediated C–H imidation of arenes and heteroarenes <i>via</i> visible light induced photocatalysis. *Chem. Commun.* **2014**, *50*, 9273-9276.
7. Kong, Y.; Cao, T.; Zhu, S., TEMPO-Regulated Regio- and Stereoselective Cross-Dihalogenation with Dual Electrophilic X+ Reagents. *Chin. J. Chem .* **2021**, *39*, 3004-3010.
8. Rubio-Presa, R.; García-Pedrero, O.; López-Matanza, P.; Barrio, P.; Rodríguez, F., Dihalogenation of Alkenes Using Combinations of N-Halosuccinimides and Alkali Metal Halides. *Eur. J. Org. Chem.* **2021**, *2021*, 4762-4766.

NMR Spectrum for *N*-halogenating agents:

Figure S1: ^{1}H NMR of Compound **5a**: 1,3-dichloro-5,5-dimethylimidazolidine-2,4-dione-^{1}H NMR (500 MHz, $CDCl_3$)

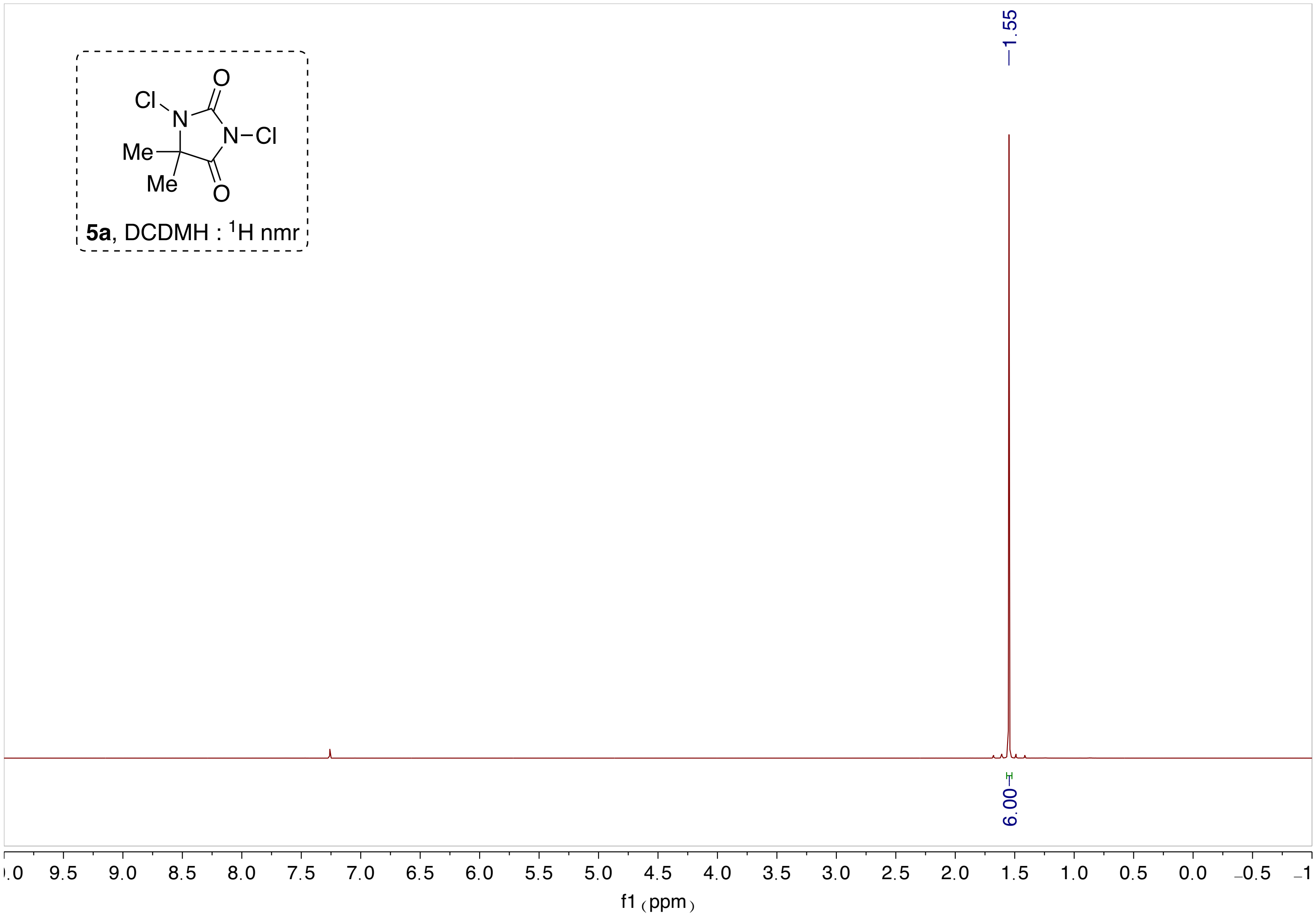

Figure S2: ^{13}C NMR of Compound **5a**: 1,3-dichloro-5,5-dimethylimidazolidine-2,4-dione- $^{13}C\{^1H\}$ (126 MHz, $CDCl_3$)

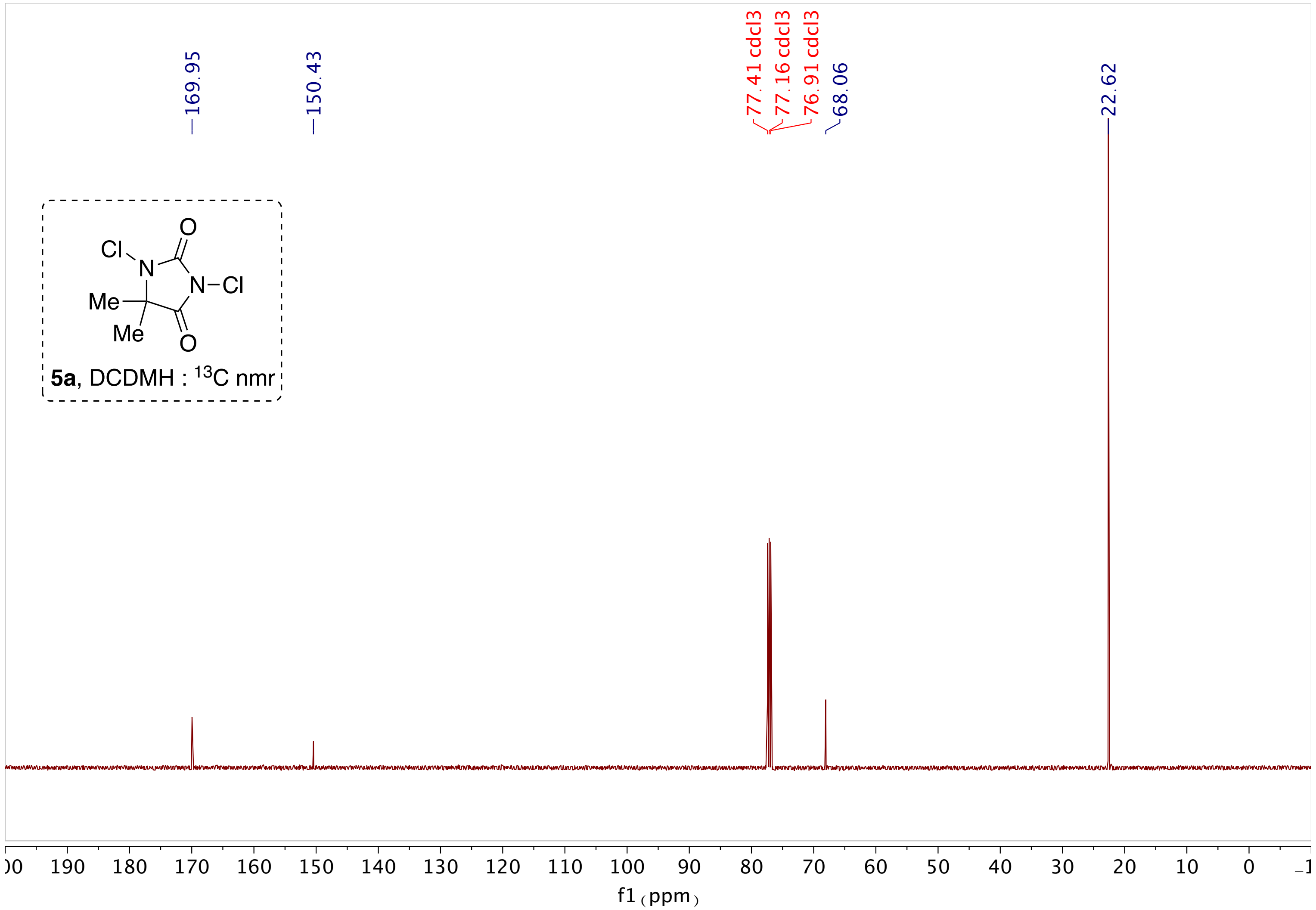

Figure S3: ^{1}H NMR of Compound **5b**: 1,3-dichloro-5,5-diphenylimidazolidine-2,4-dione-^{1}H NMR (500 MHz, $CDCl_3$)

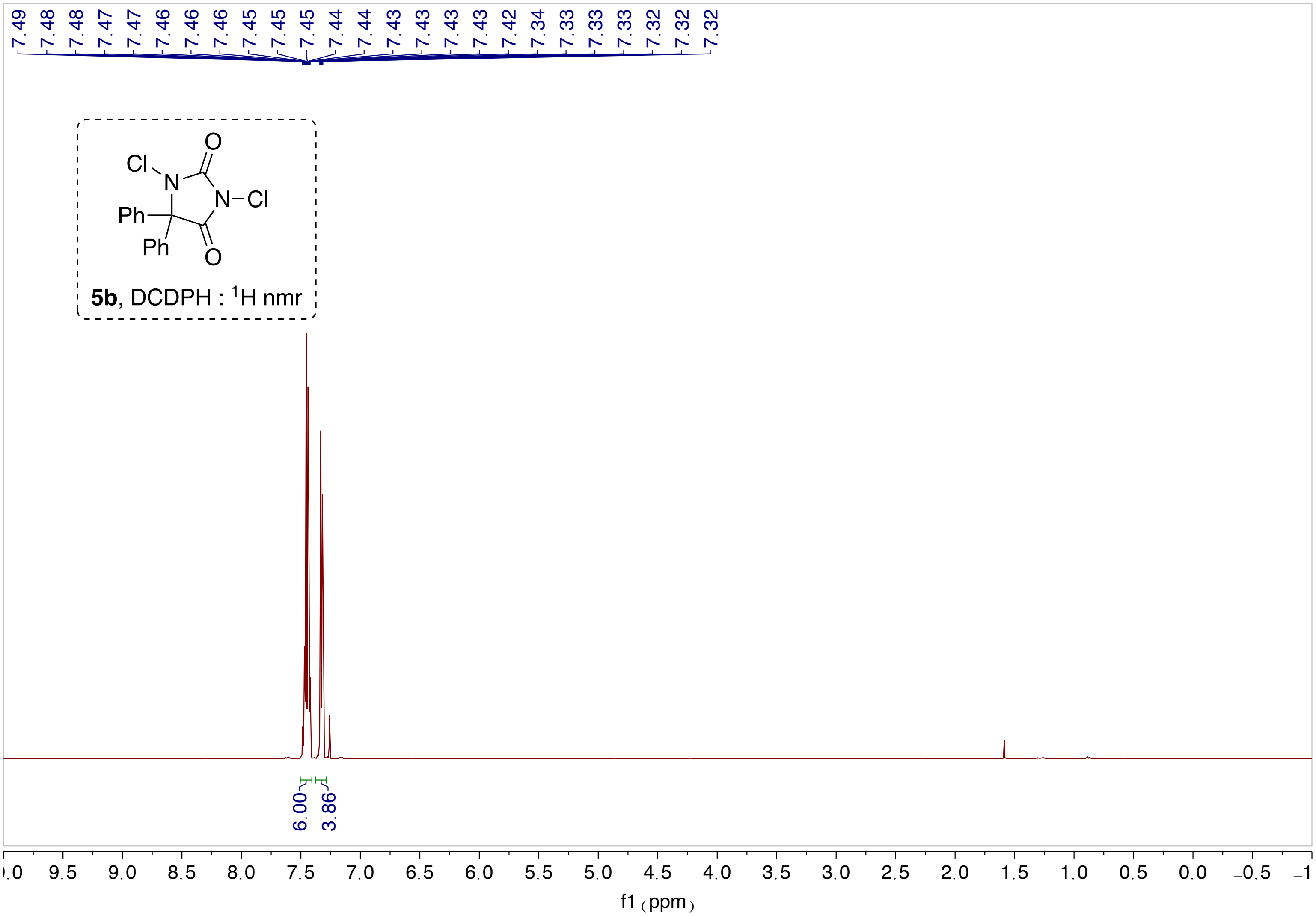

Figure S4: ^{13}C NMR of Compound **5b**: 1,3-dichloro-5,5-diphenylimidazolidine-2,4-dione- $^{13}C\{^1H\}$ (126 MHz, $CDCl_3$)

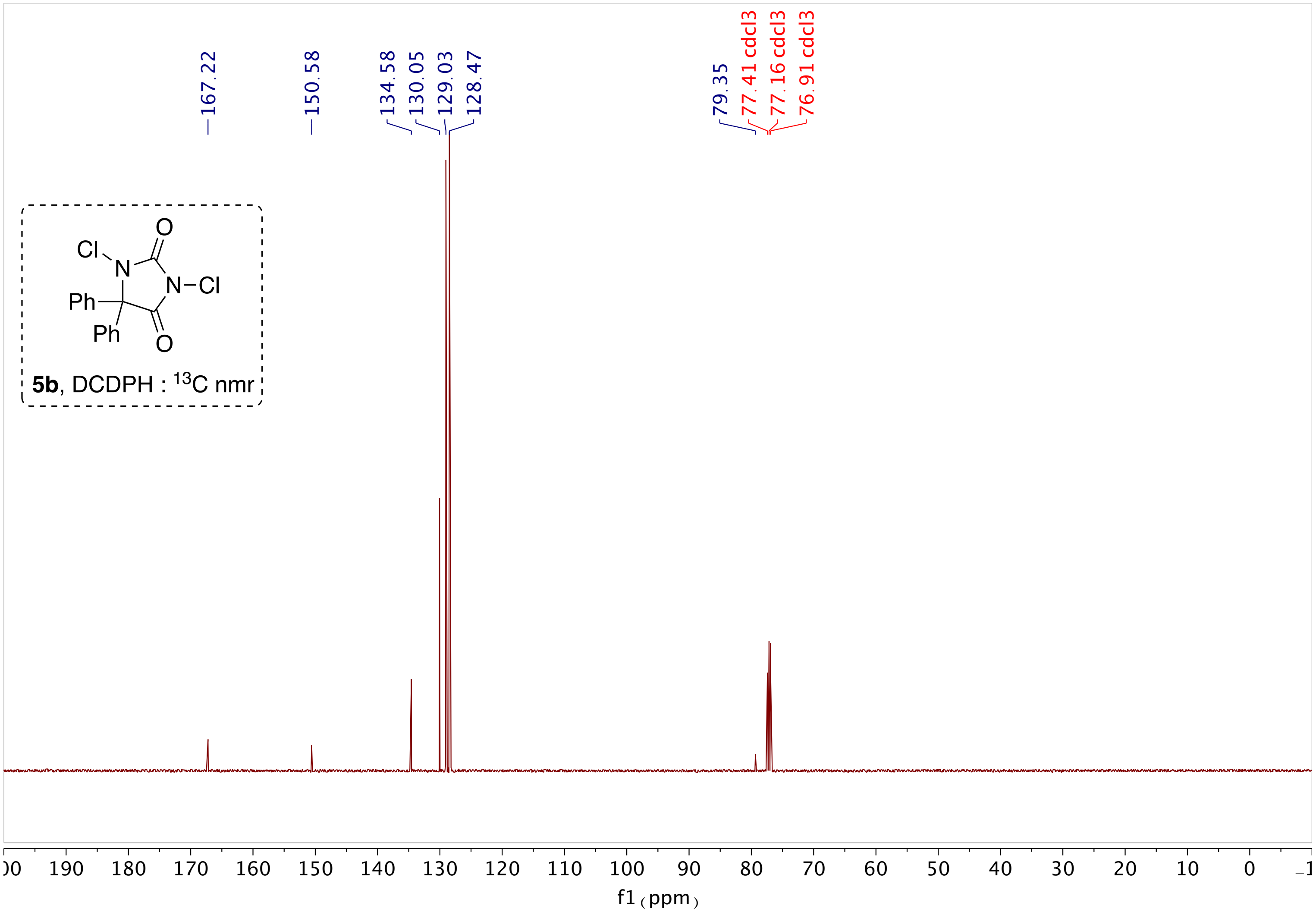

Figure S5: ^{1}H NMR of Compound **5c**: 1,3-dichloro-imidazolidine-2,4-dione-^{1}H NMR (500 MHz, $CDCl_3$)

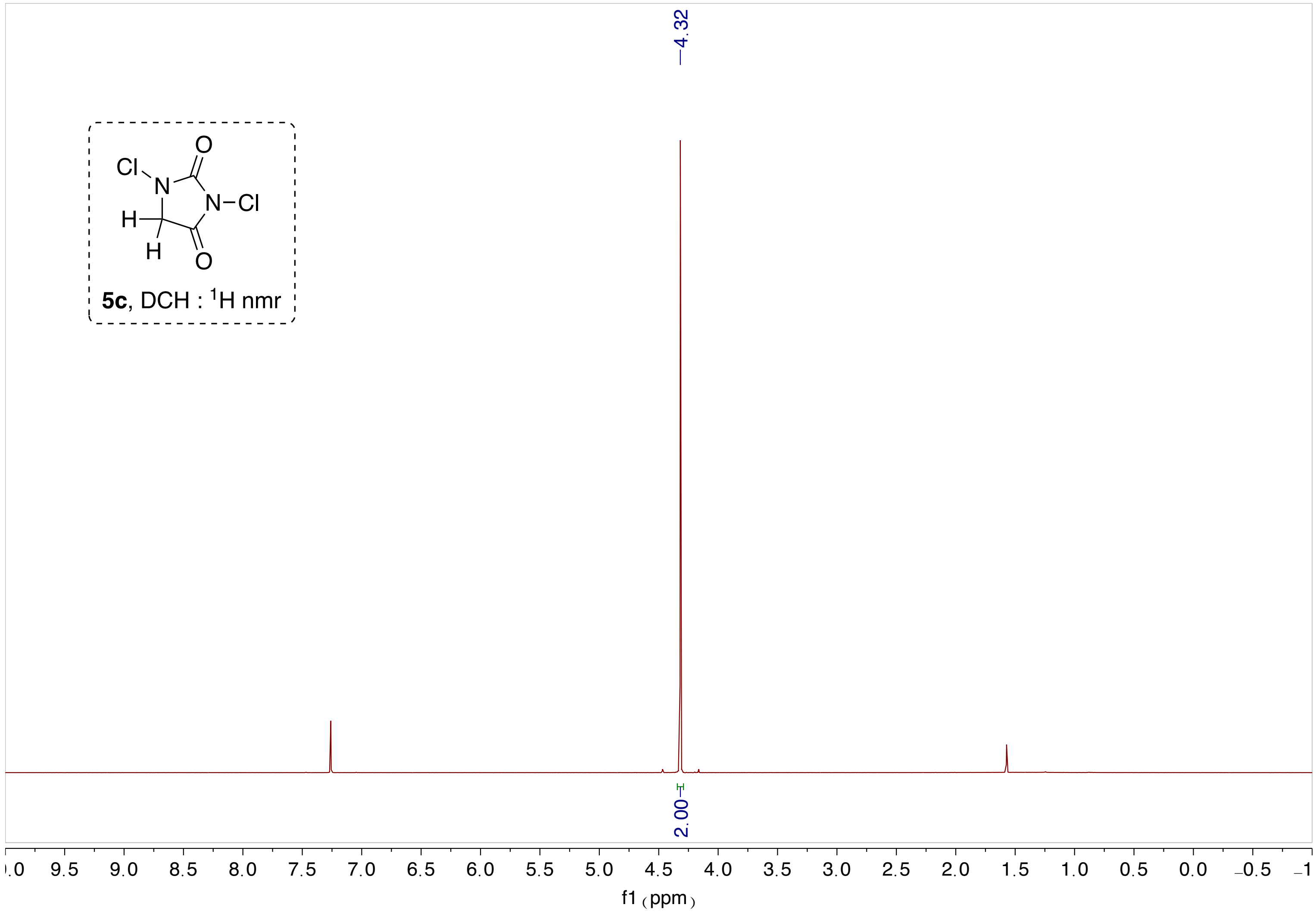

Figure S6: ^{13}C NMR of Compound **5c**: 1,3-dichloro-imidazolidine-2,4-dione-^{13}C{^{1}H} (126 MHz, $CDCl_3$)

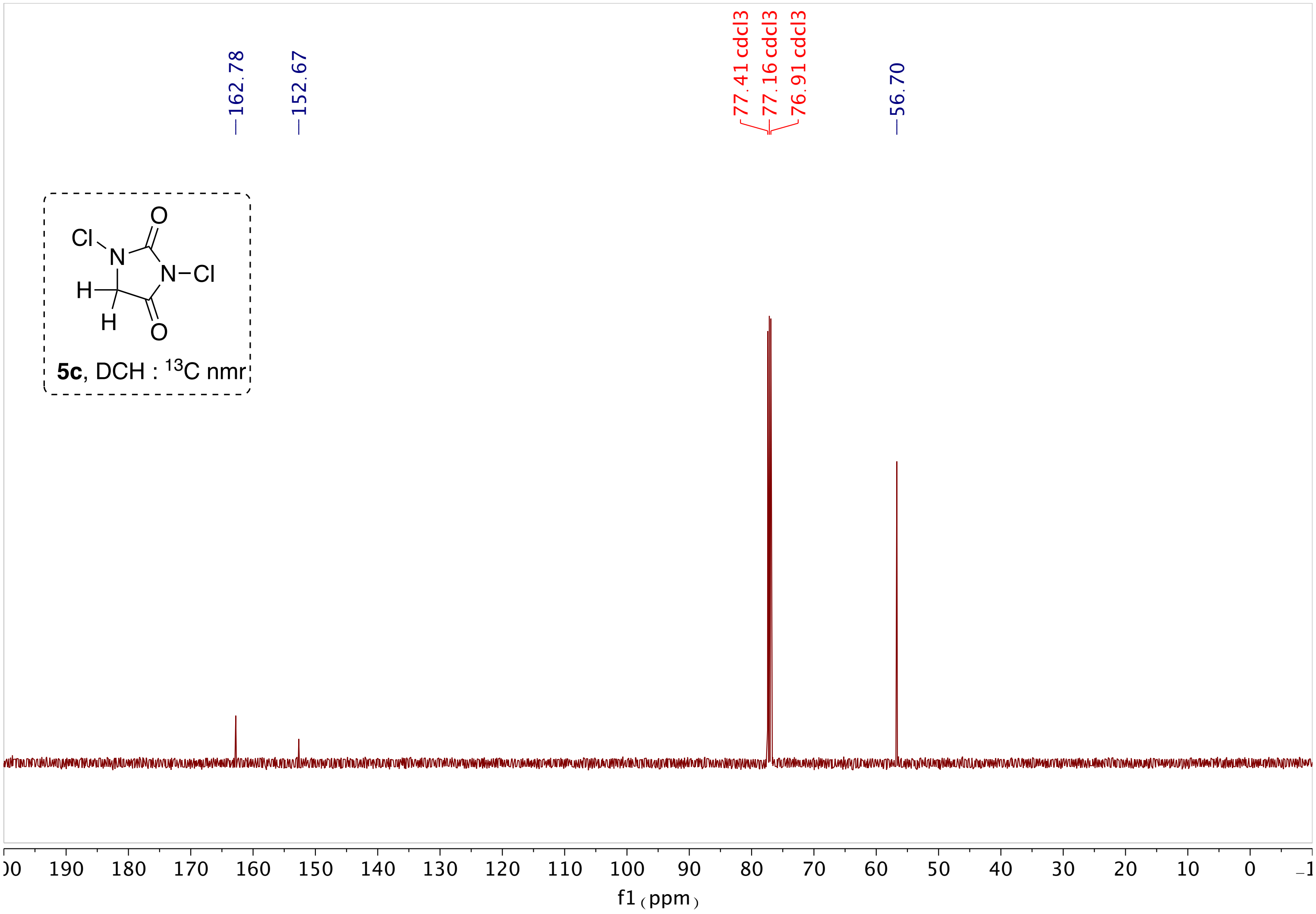

Figure S7: ^{1}H NMR of Compound **5d**: 1-chloropyrrolidine-2,5-dione -^{1}H NMR (500 MHz, $CDCl_3$)

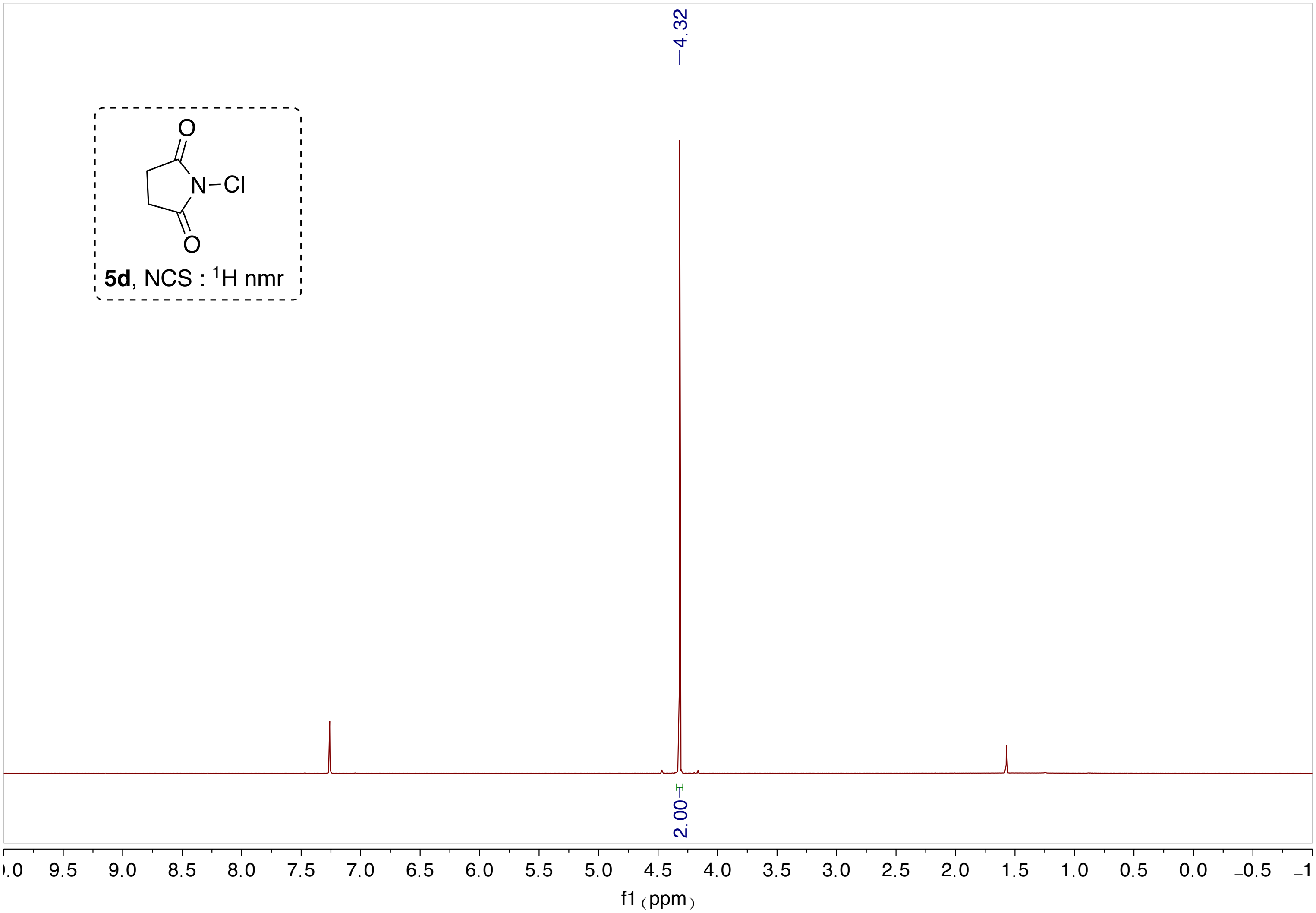

Figure S8: ^{13}C NMR of Compound **5d**: 1-chloropyrrolidine-2,5-dione -$^{13}C\{^1H\}$ (126 MHz, $CDCl_3$)

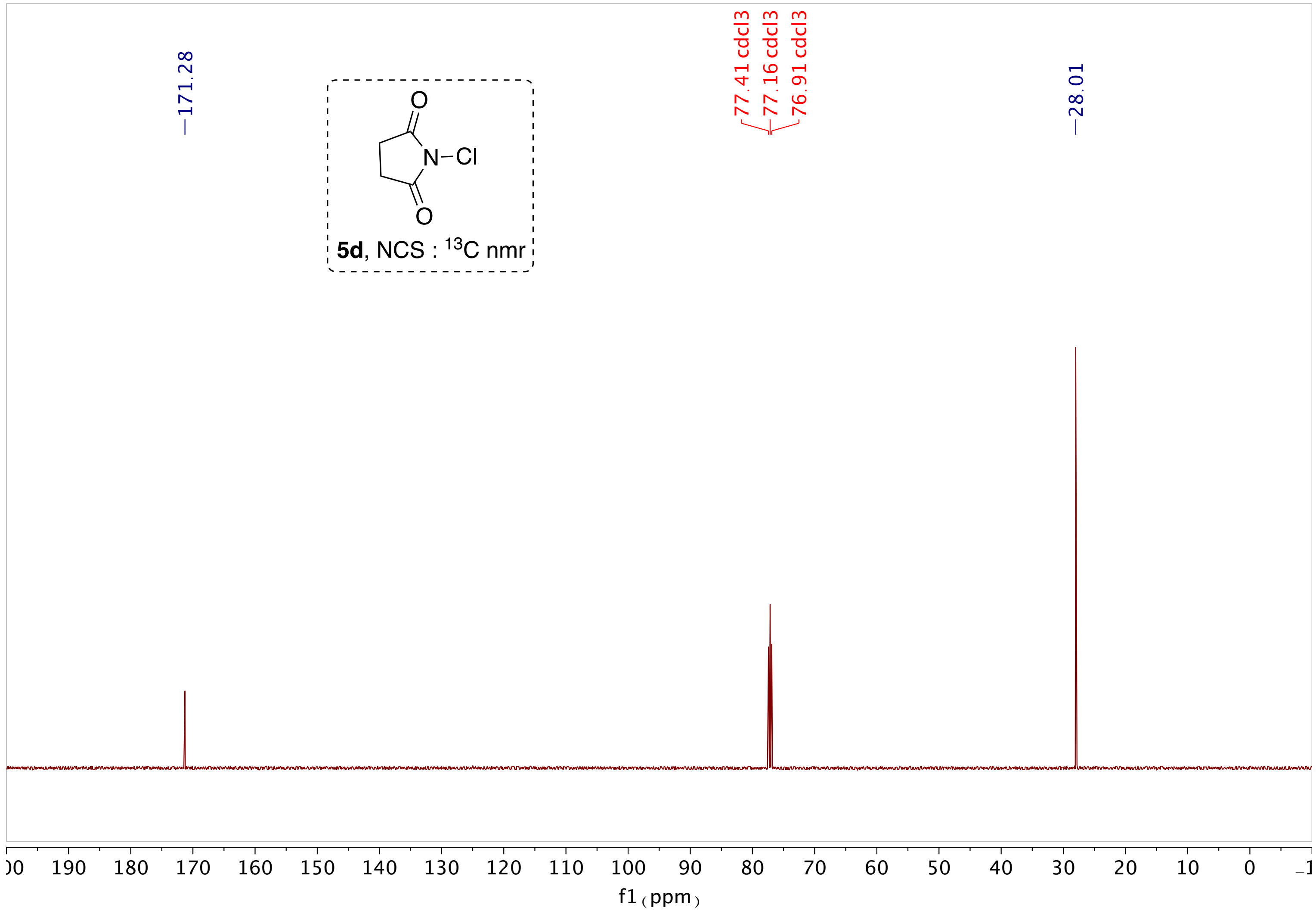

Figure S9: ^{1}H NMR of Compound **5e**: 2-chloroisoindoline-1,3-dione -^{1}H NMR (500 MHz, $CDCl_3$)

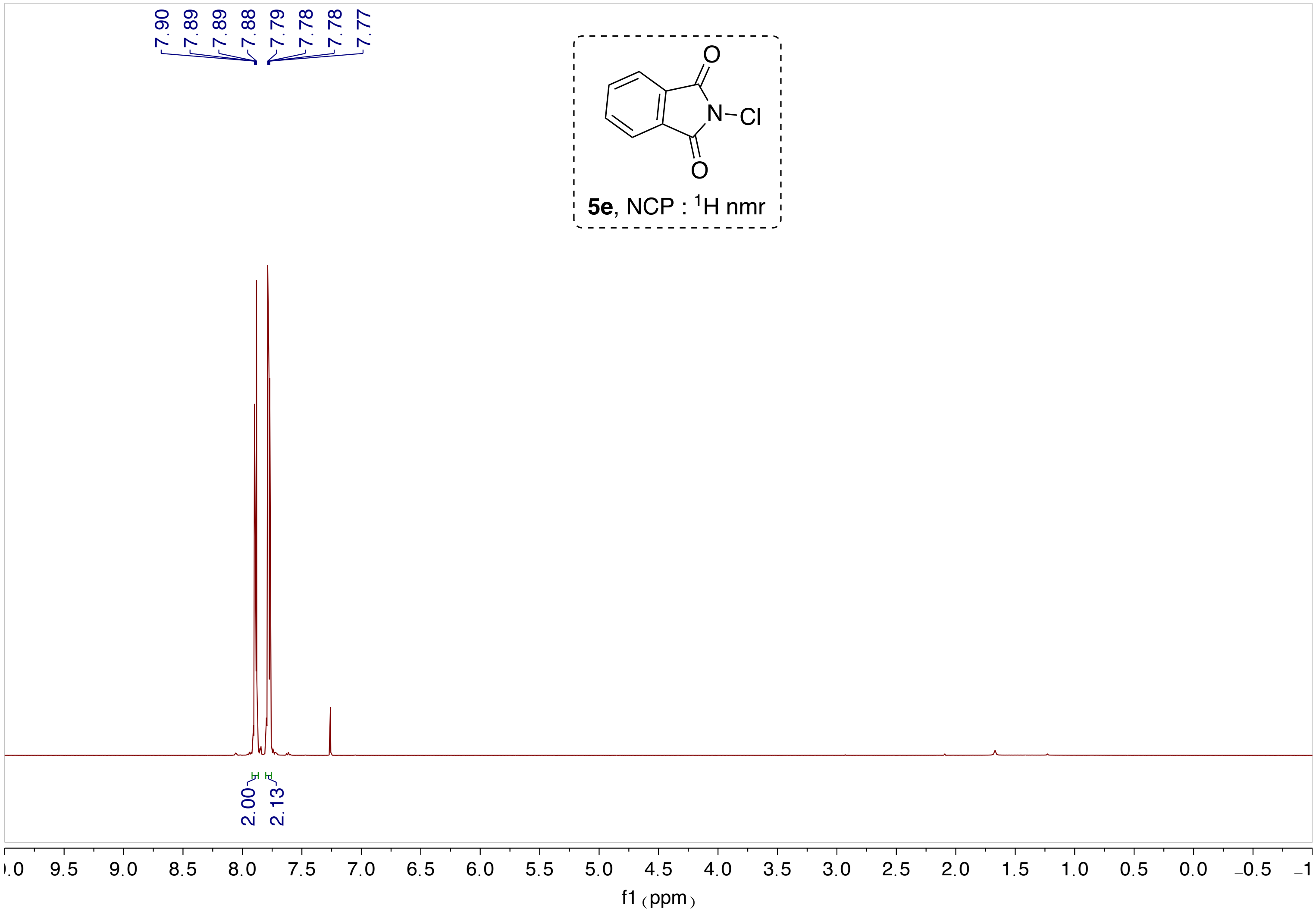

Figure S10: ^{13}C NMR of Compound **5e**: 2-chloroisoindoline-1,3-dione -^{13}C{^{1}H} (126 MHz, $CDCl_3$)

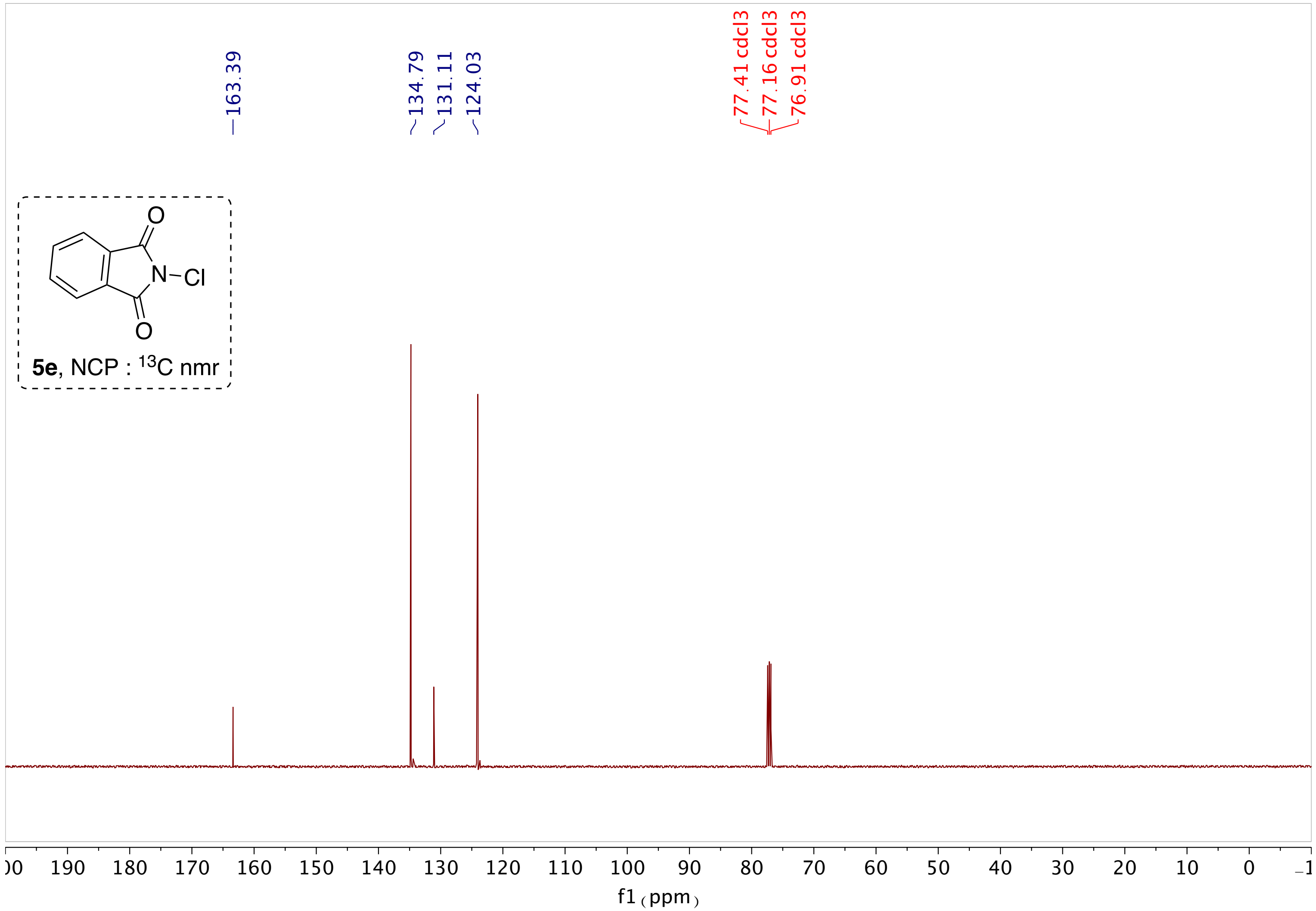

Figure S11: ^{1}H NMR of Compound **5f**: 1-chloropyrrolidin-2-one -^{1}H NMR (500 MHz, $CDCl_3$)

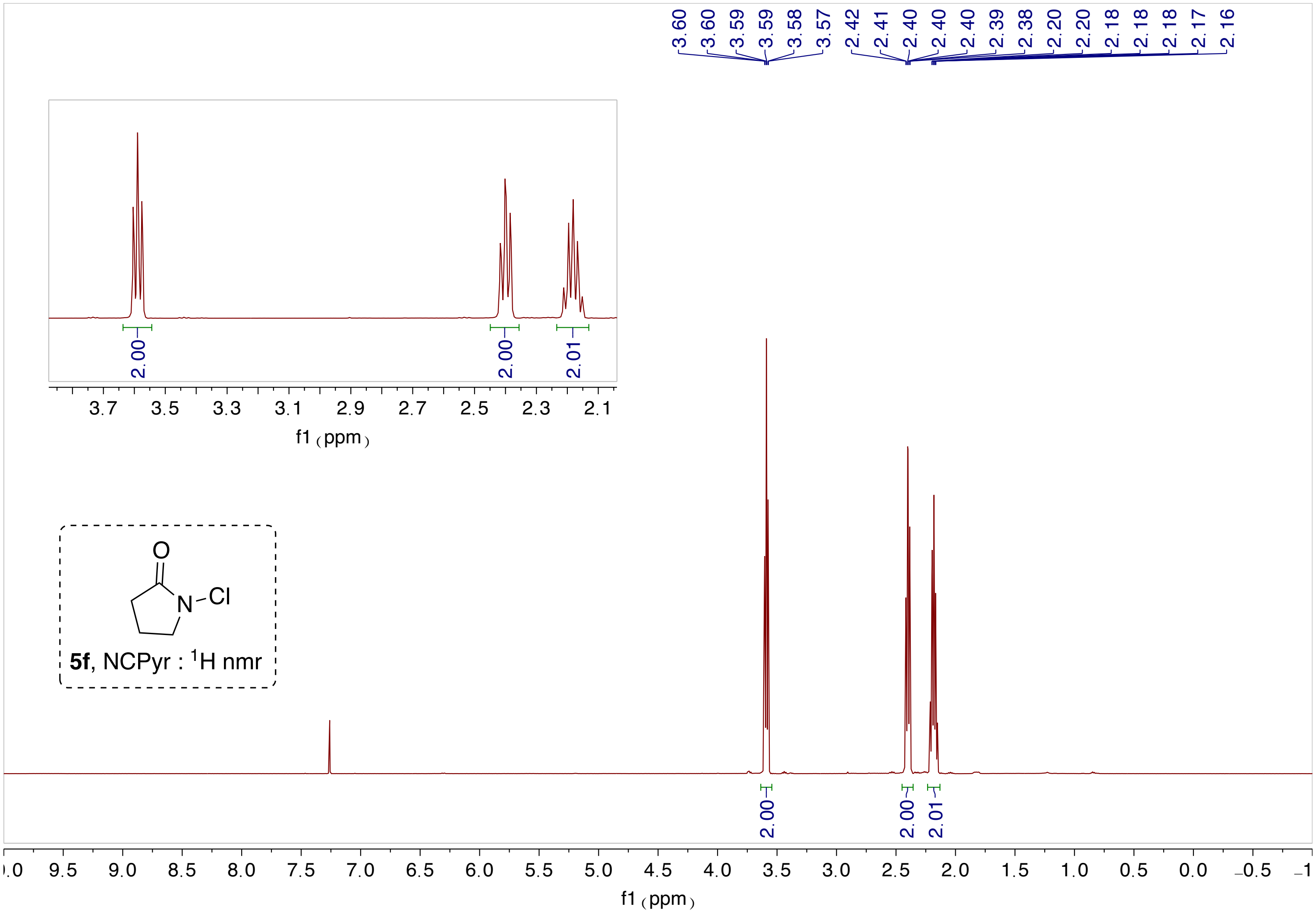

Figure S12: ^{13}C NMR of Compound **5f**: 1-chloropyrrolidin-2-one- $^{13}C\{^1H\}$ (126 MHz, $CDCl_3$)

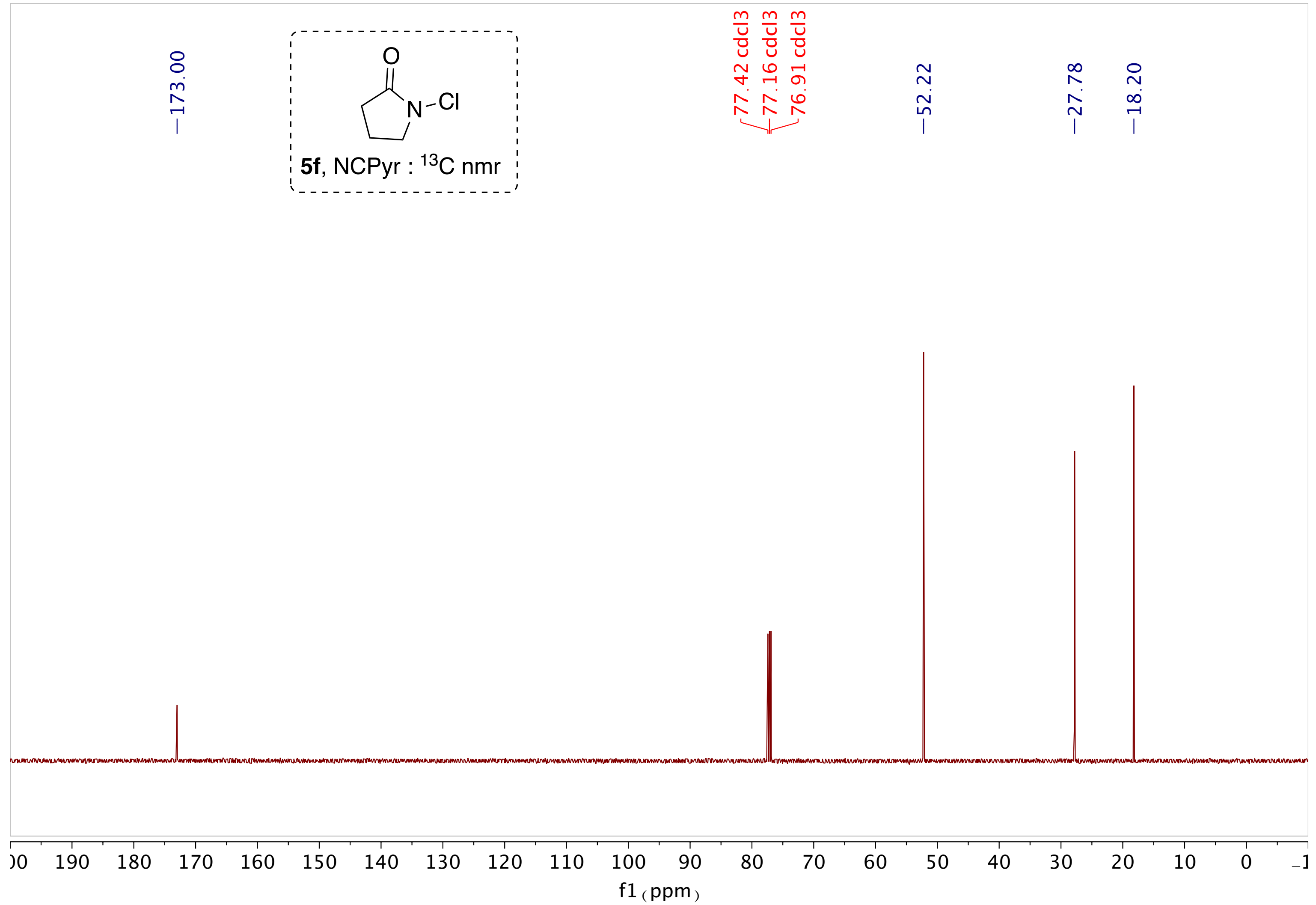

Figure S13: ^{1}H NMR of Compound **5g**: *N*-chloroacetamide -^{1}H NMR (500 MHz, $CDCl_3$)

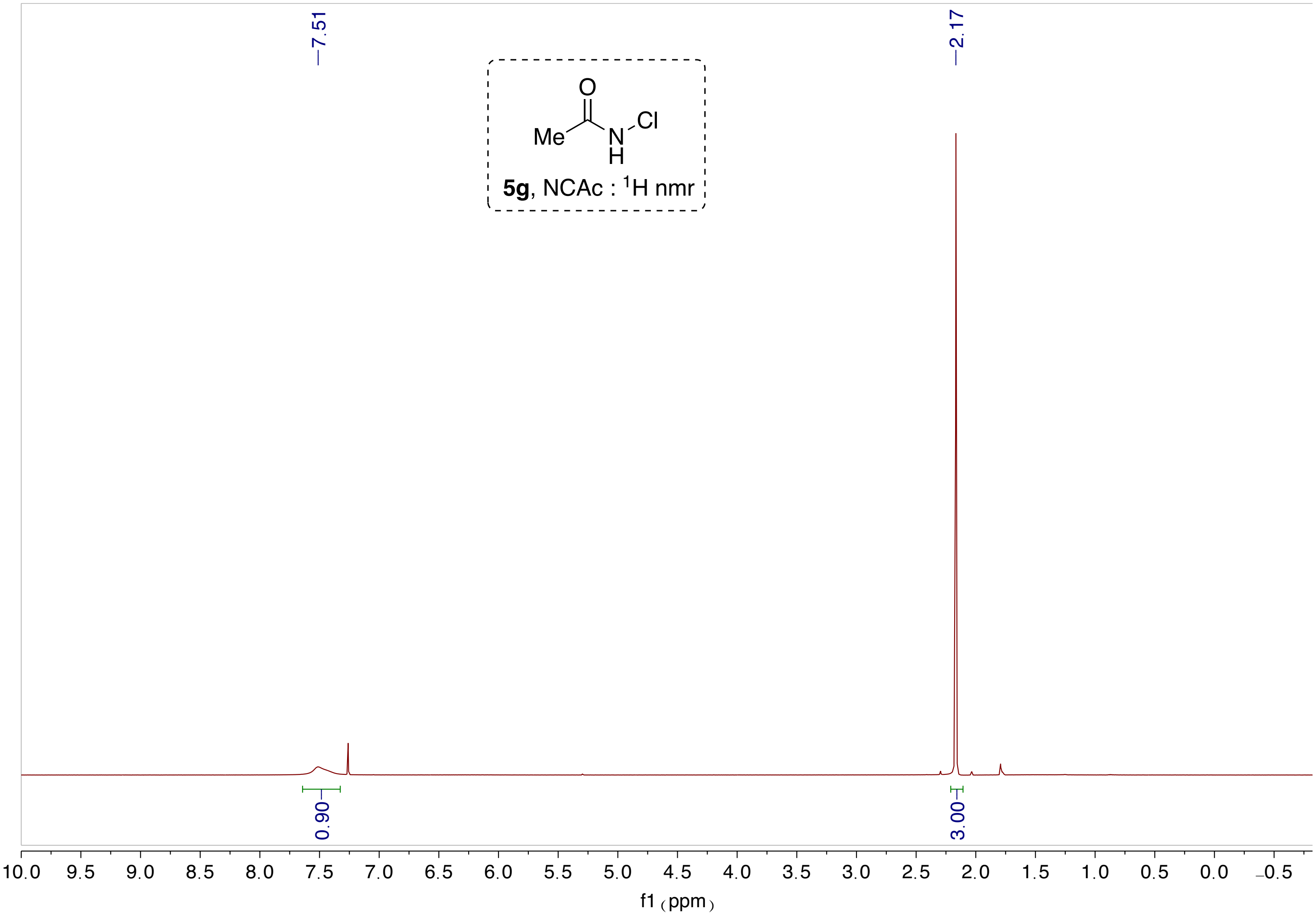

Figure S14: ^{13}C NMR of Compound **5g**: *N*-chloroacetamide -^{13}C{^{1}H} (126 MHz, $CDCl_3$)

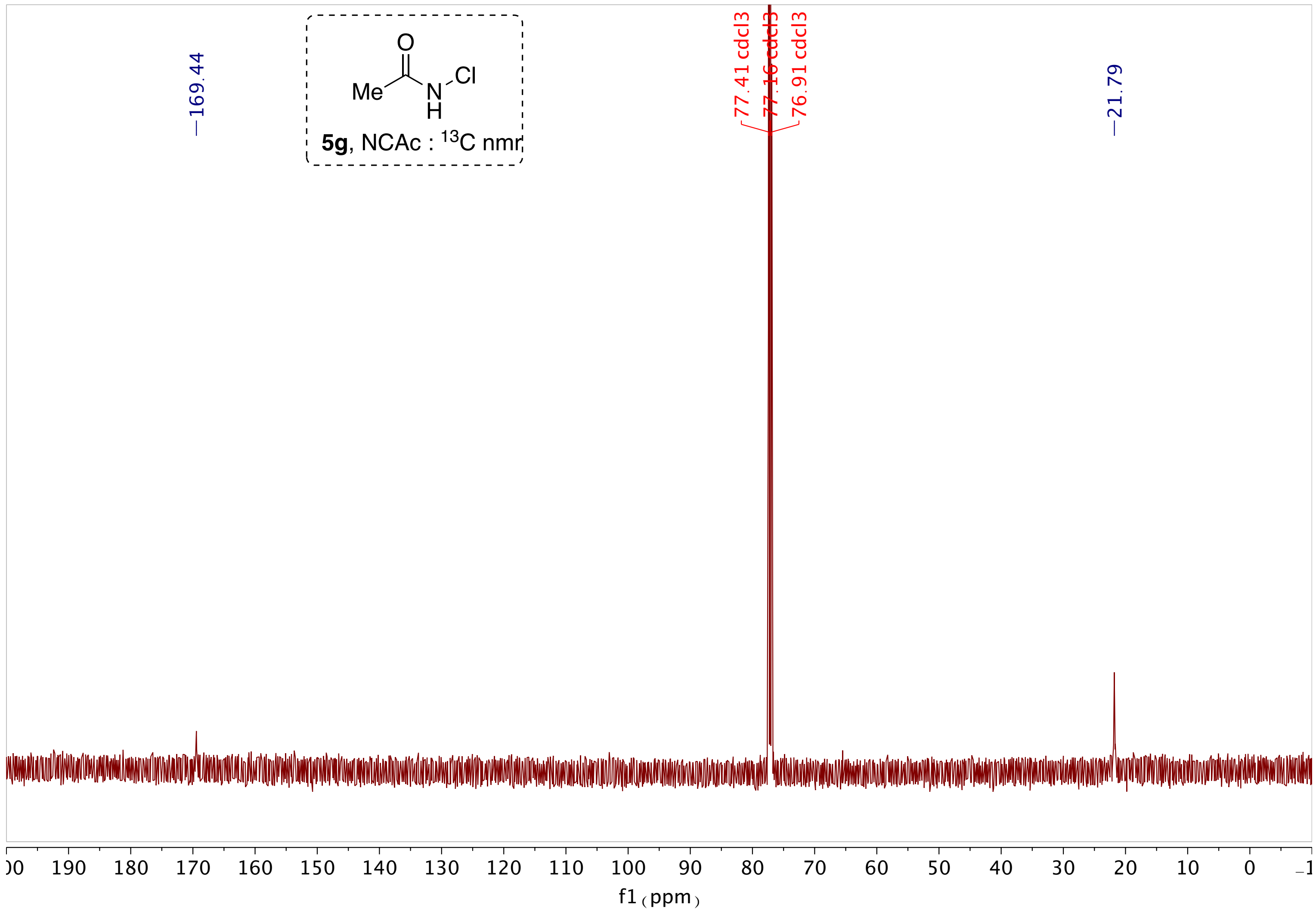

Figure S15: ^{1}H NMR of Compound **5h**: 1,3,5-trichloro-1,3,5-triazinane-2,4,6-trione -$^{13}C\{^{1}H\}$ (126 MHz, CD_3CN)

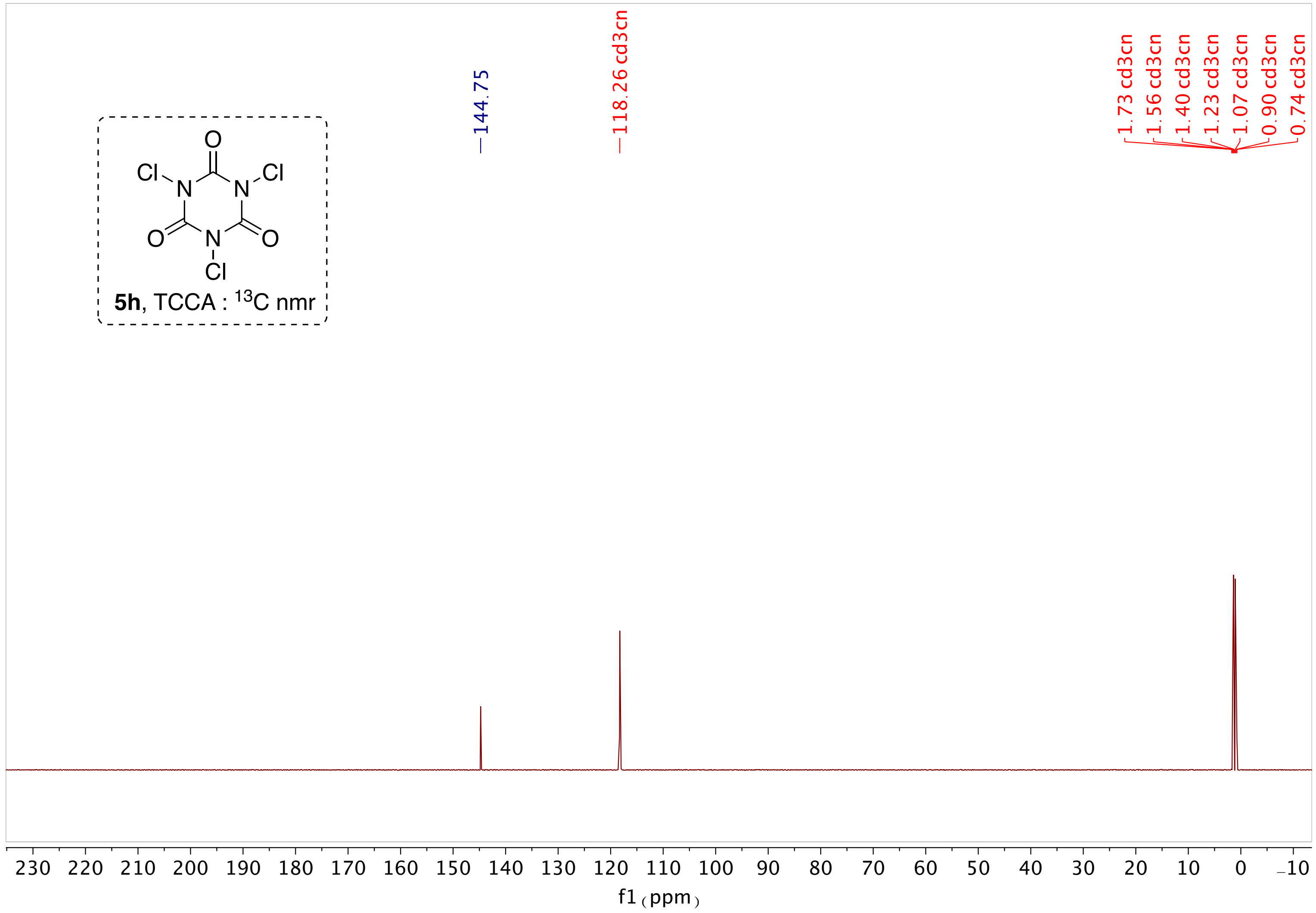

Figure S16: ^{1}H NMR of Compound **5i**: 2-chlorobenzo[*d*]isothiazol-3(2*H*)-one 1,1-dioxide -^{1}H NMR (500 MHz, $CDCl_3$)

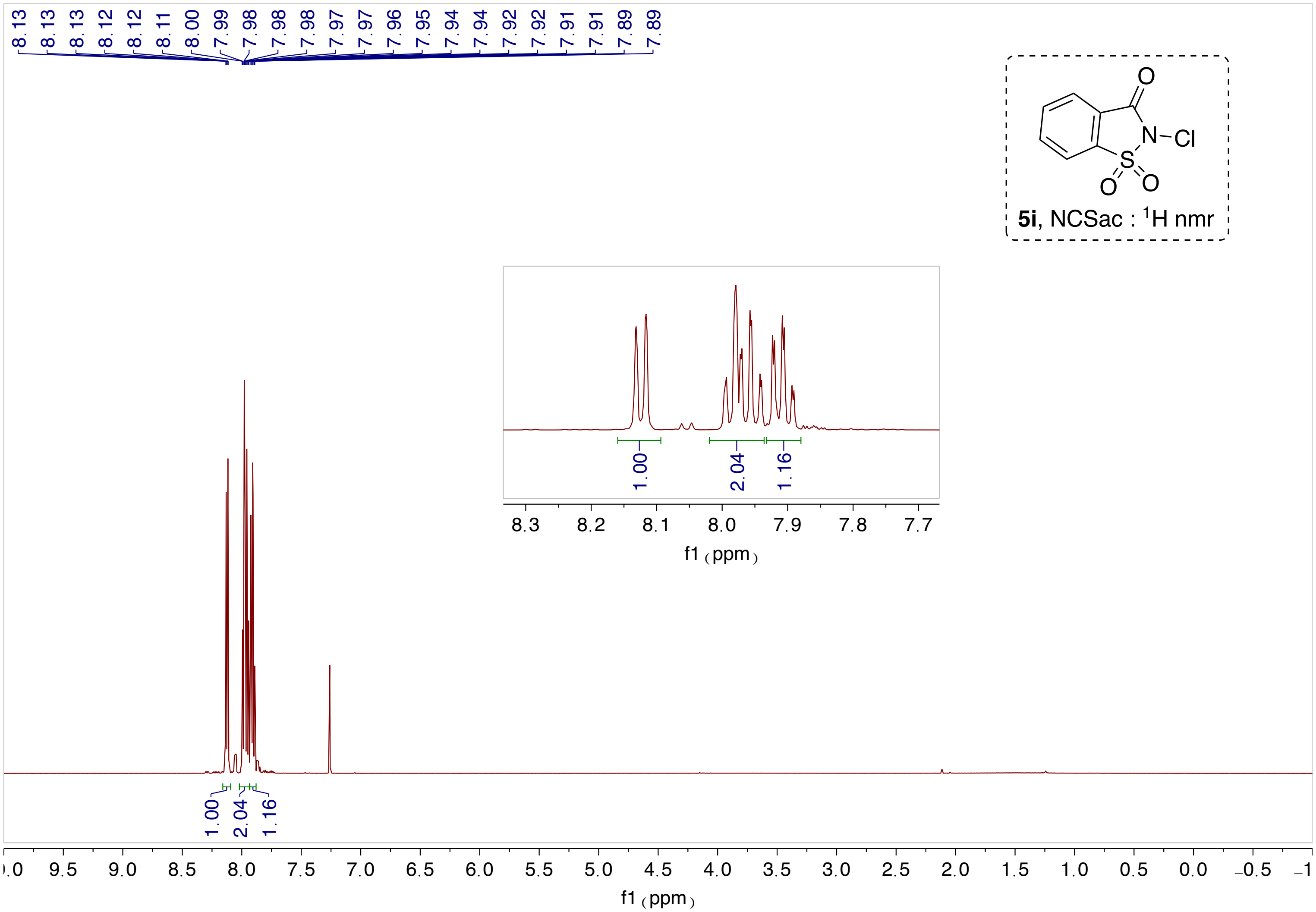

Figure S17: ^{13}C NMR of Compound **5i**: 2-chlorobenzo[*d*]isothiazol-3(2*H*)-one 1,1-dioxide- $^{13}C\{^{1}H\}$ (126 MHz, $CDCl_3$)

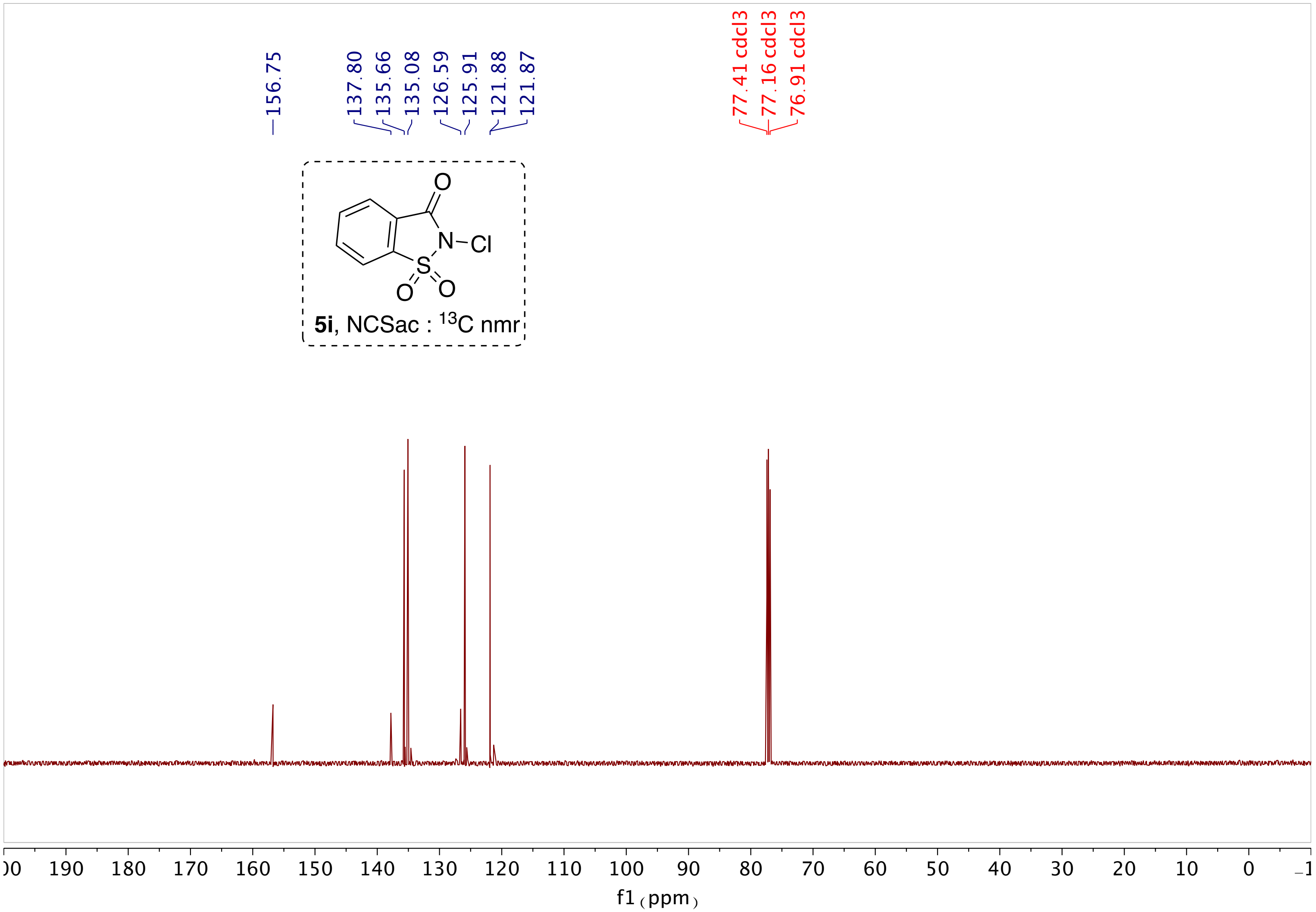

Figure S18: ^{1}H NMR of Compound **5j**: Tosylchlorimidous chloride -^{1}H NMR (500 MHz, $CDCl_3$)

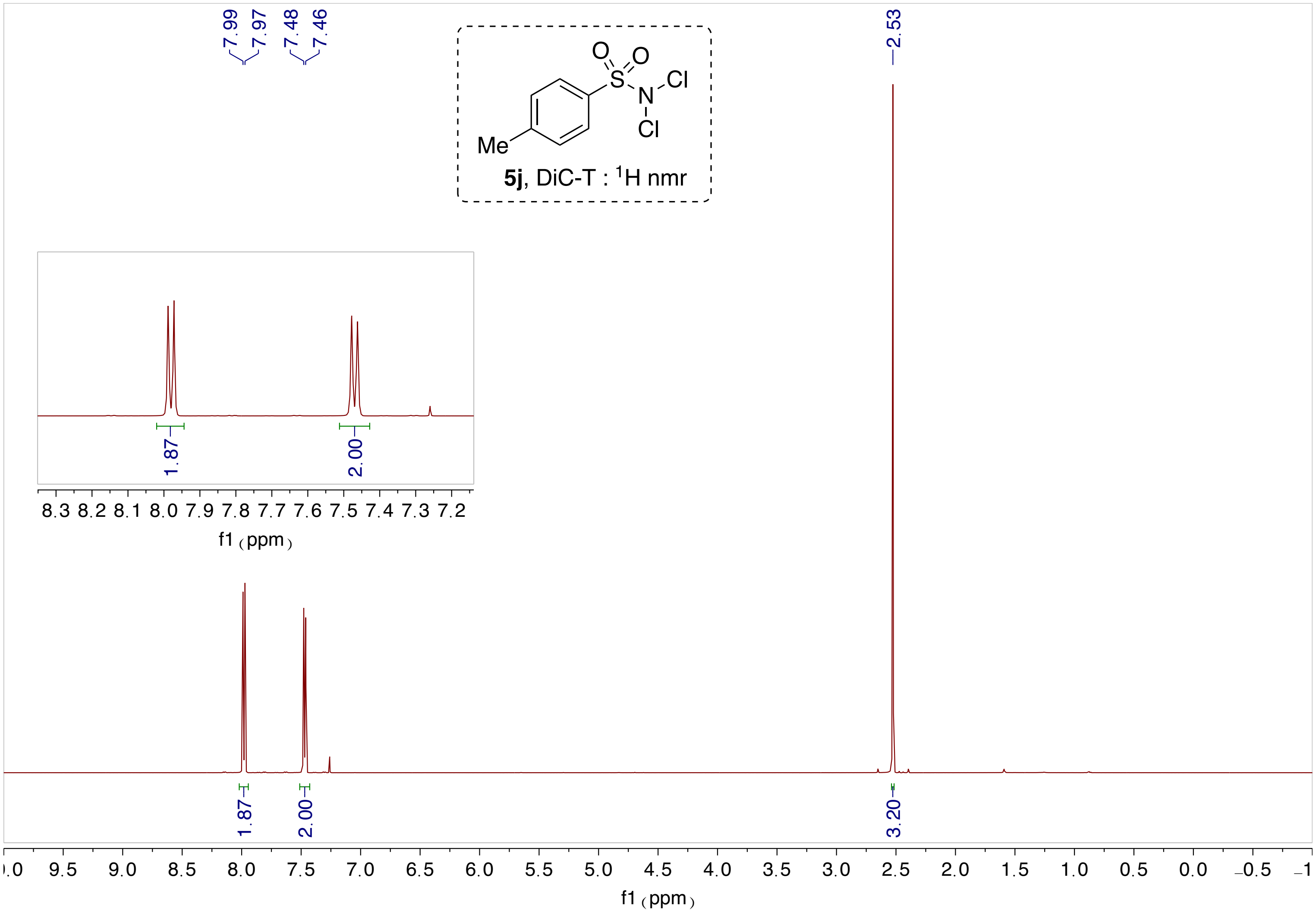

Figure S19: ^{13}C NMR of Compound **5j**: Tosylchlorimidous chloride -$^{13}C\{^1H\}$ (126 MHz, $CDCl_3$)

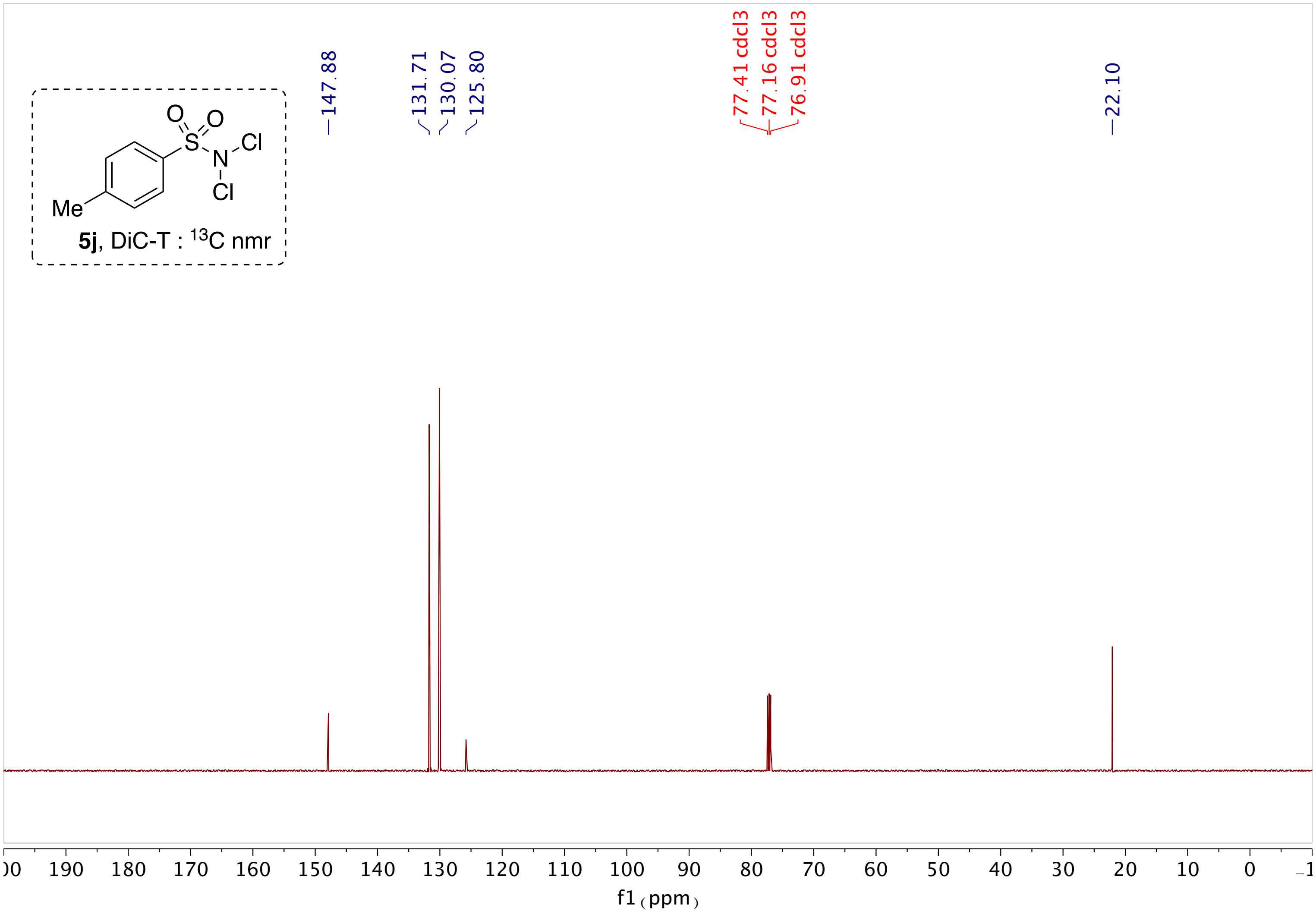

Figure S20: ^{1}H NMR of Compound **6a**: 1,3-dibromo-5,5-dimethylimidazolidine-2,4-dione-^{1}H NMR (500 MHz, $CDCl_3$)

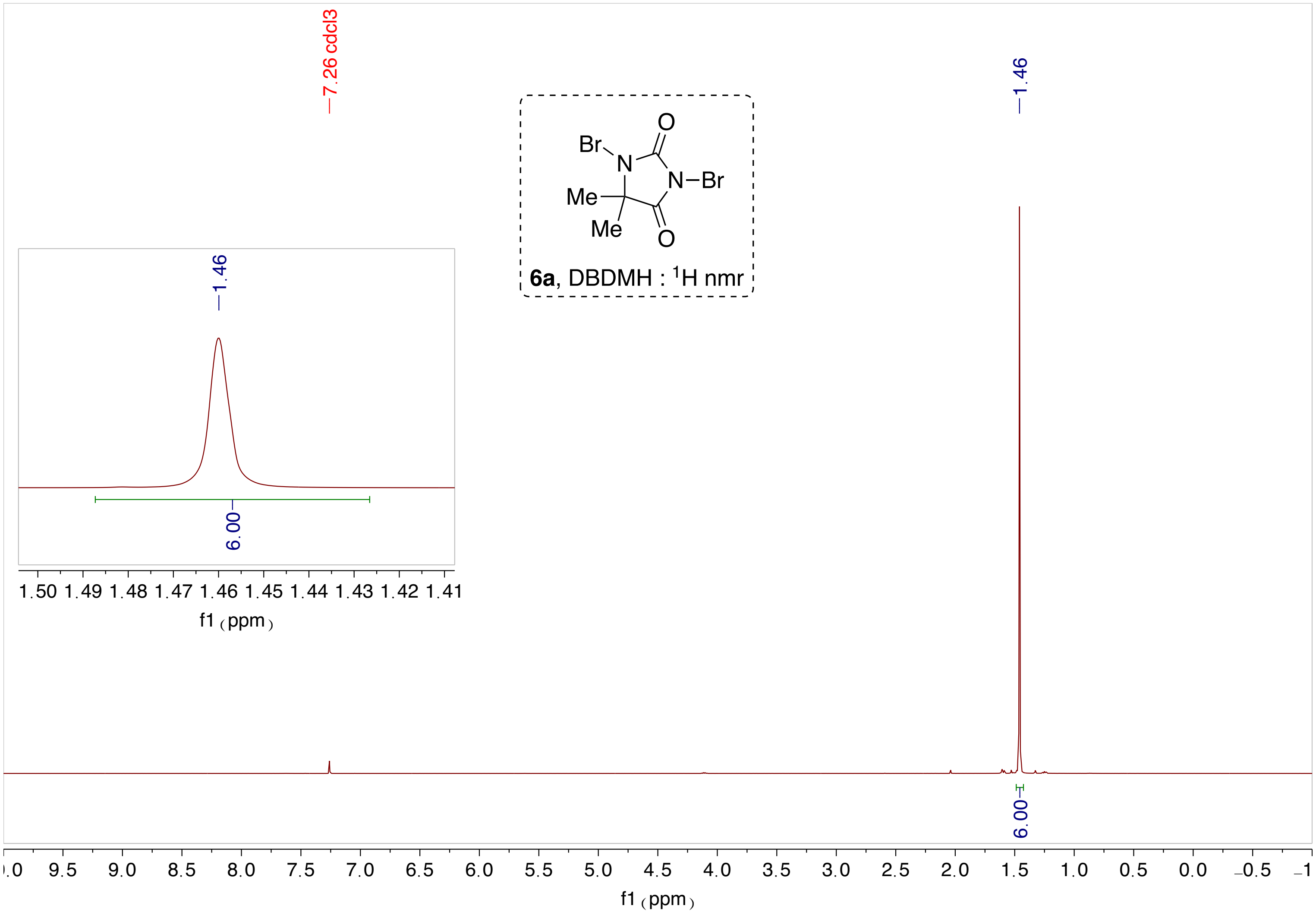

Figure S21: ^{13}C NMR of Compound **6a**: 1,3-dibromo-5,5-dimethylimidazolidine-2,4-dione-^{13}C{^{1}H} (126 MHz, CDCl$_3$)

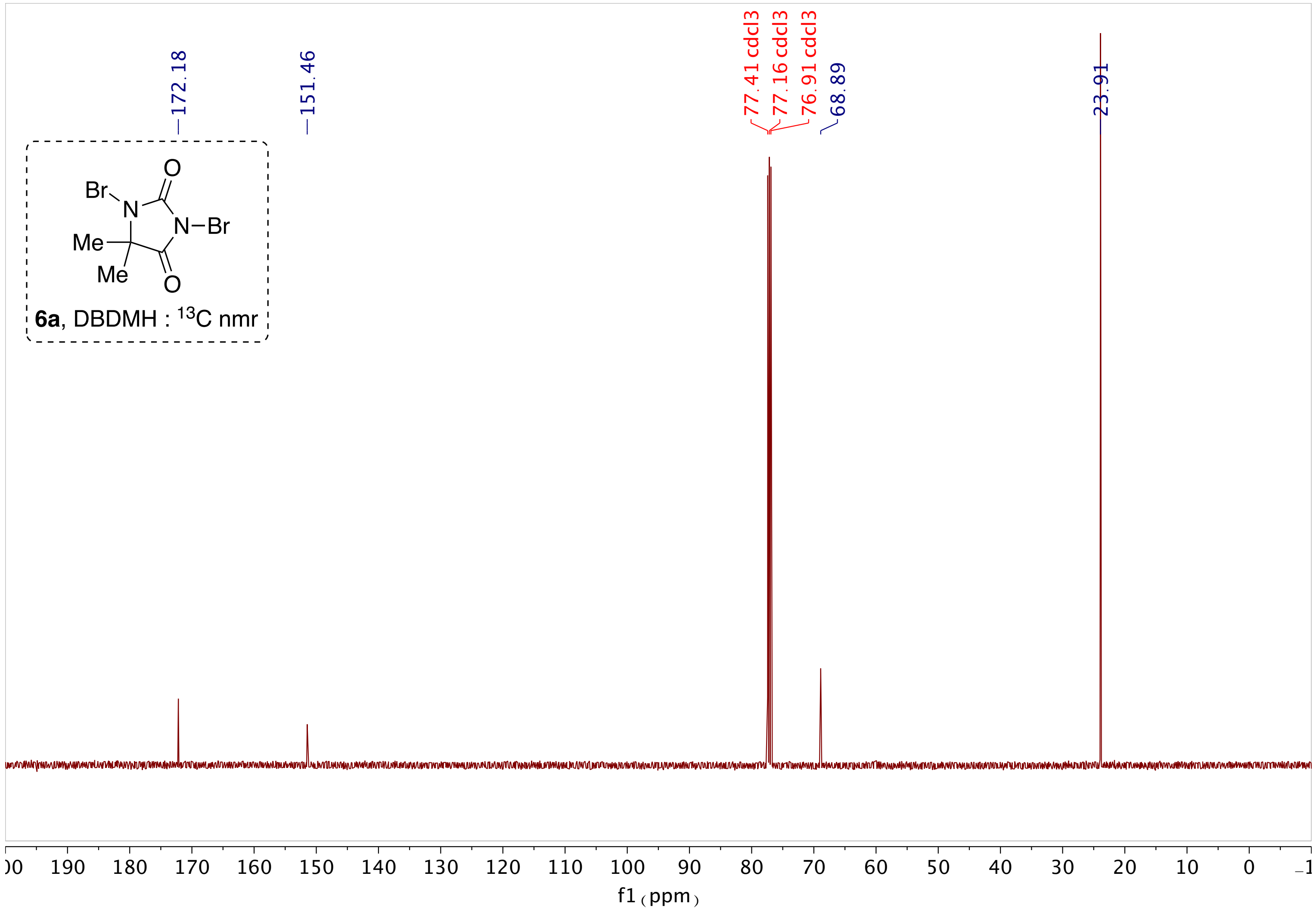

Figure S22: ¹H NMR of Compound **6b**: 1,3-dibromo-5,5-diphenylimidazolidine-2,4-dione-¹H NMR (500 MHz, CD_3OD)

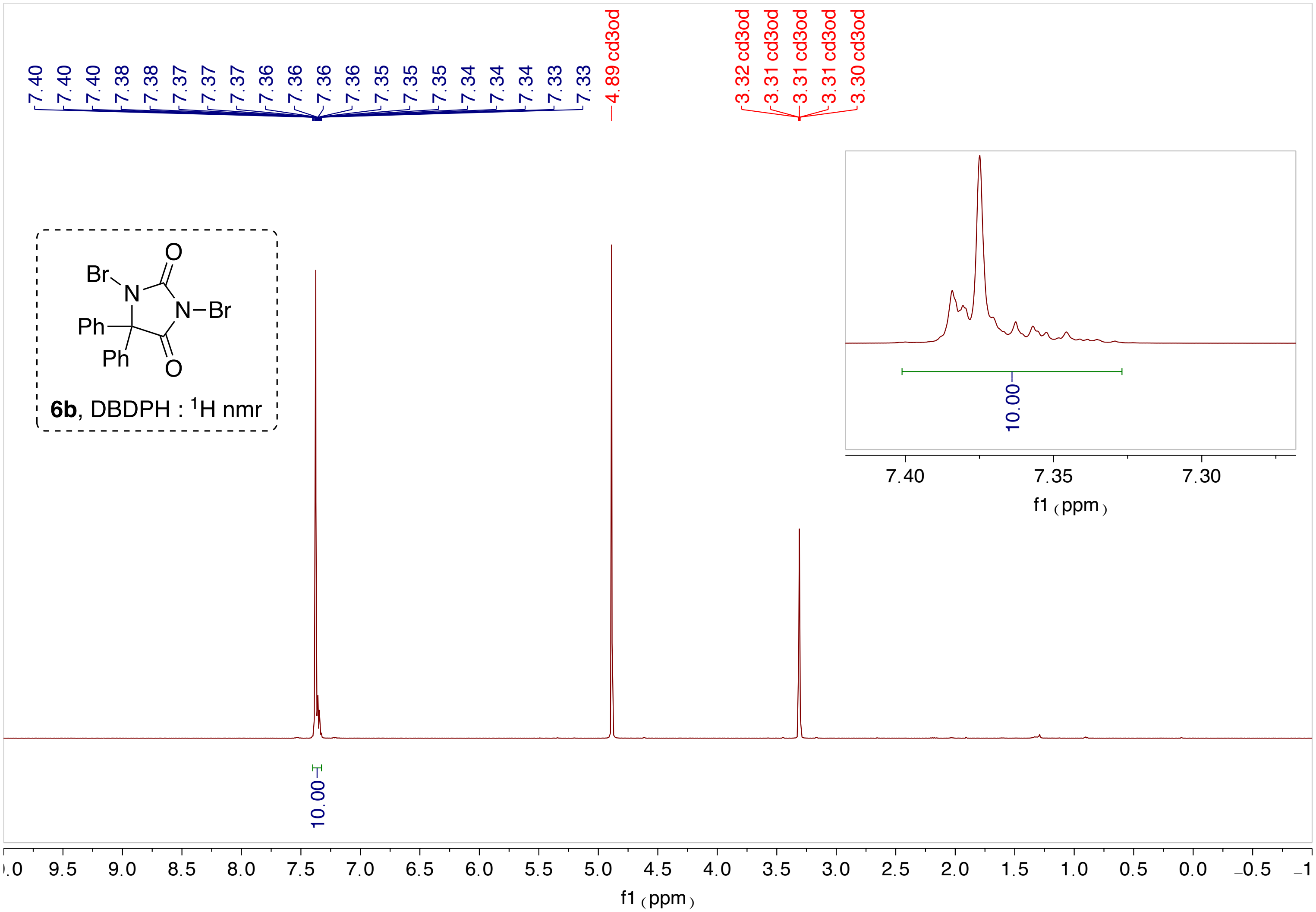

Figure S23: ^{13}C NMR of Compound **6b**: 1,3-dibromo-5,5-diphenylimidazolidine-2,4-dione-^{13}C NMR (126 MHz, CD_3OD)

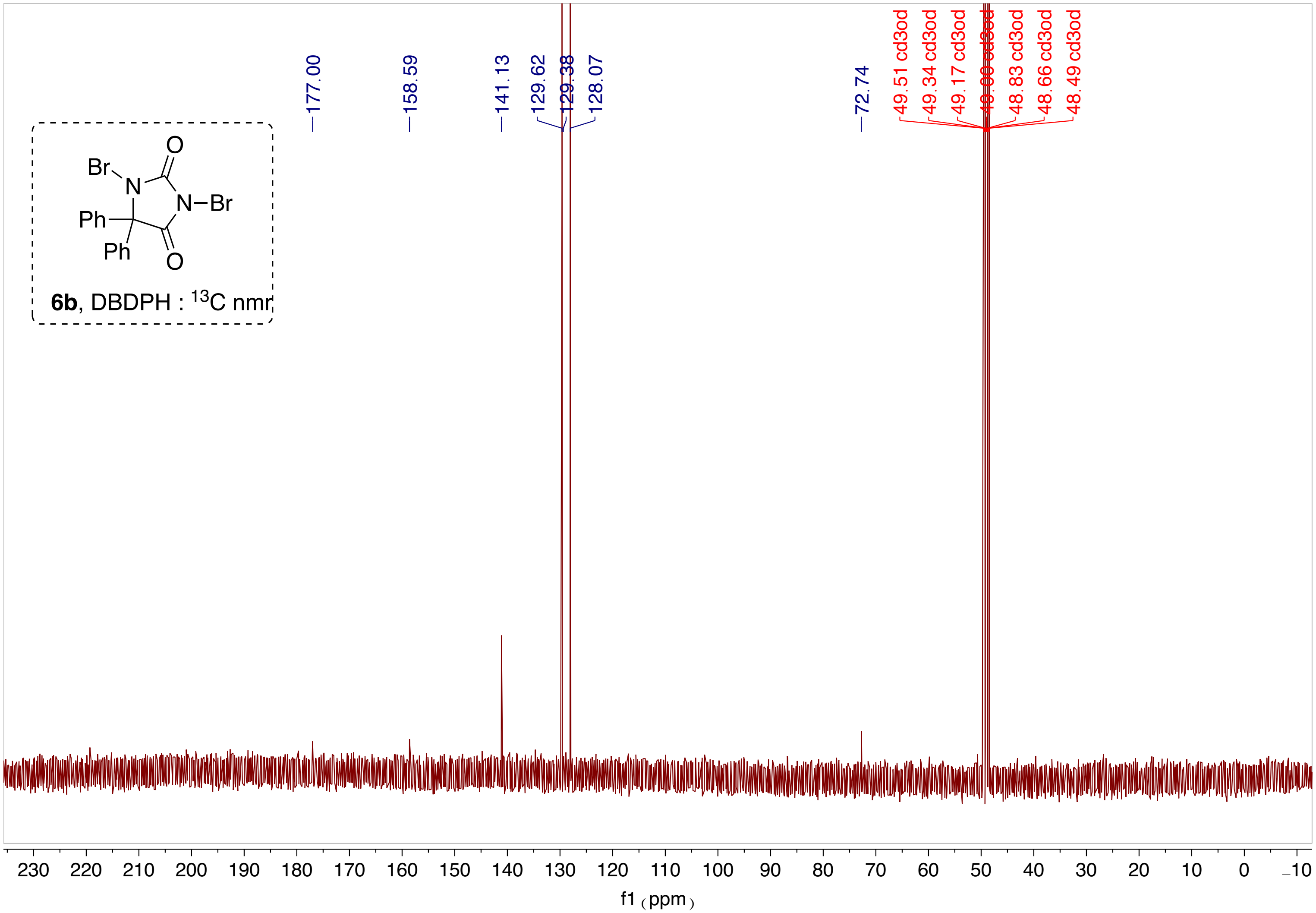

Figure S24: ^{1}H NMR of Compound **6c**: 1,3-dibromoimidazolidine-2,4-dione -^{1}H NMR (500 MHz, CD_3OD)

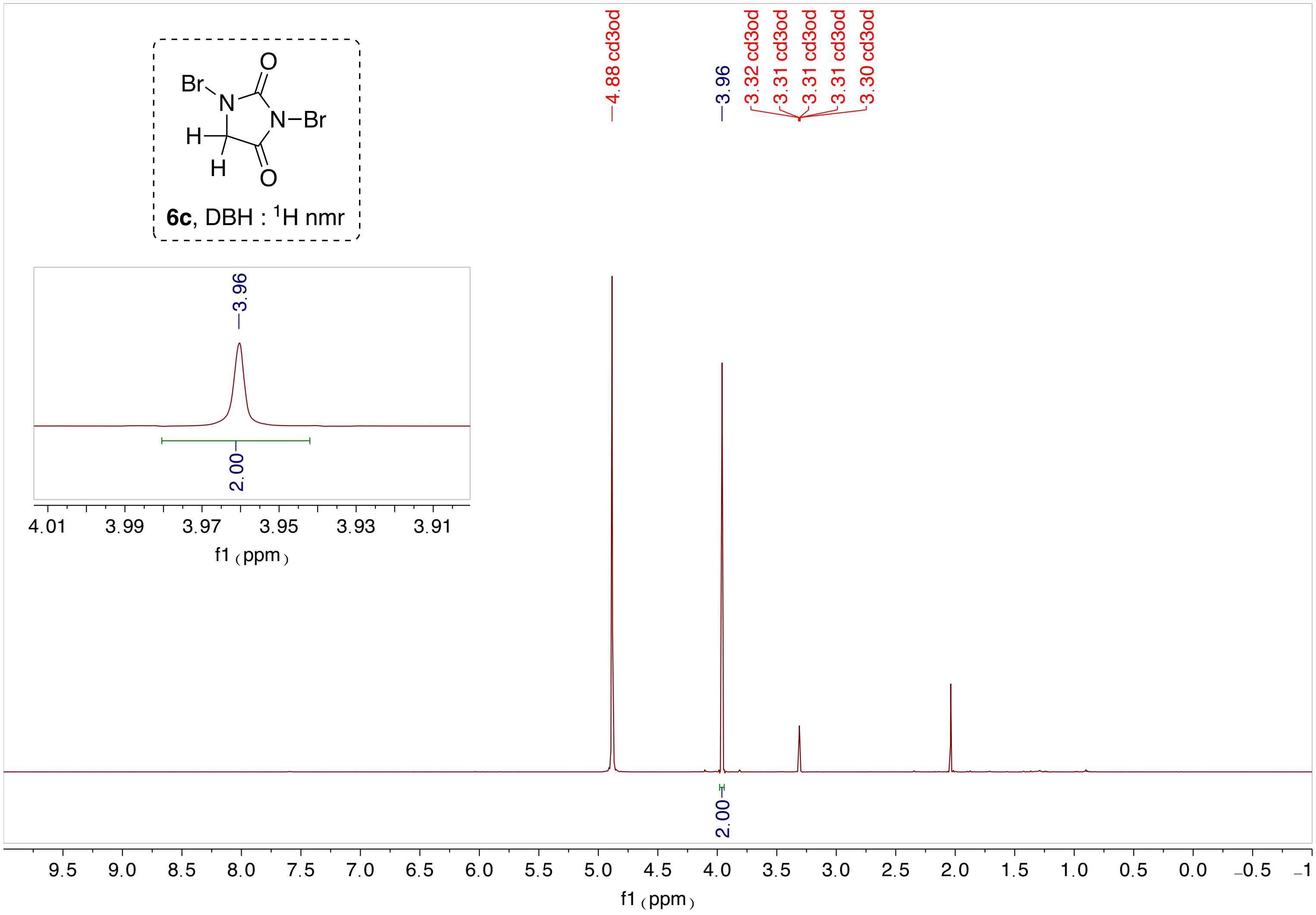

Figure S25: ^{13}C NMR of Compound **6c**: 1,3-dibromoimidazolidine-2,4-dione -^{13}C NMR (126 MHz, CD_3OD)

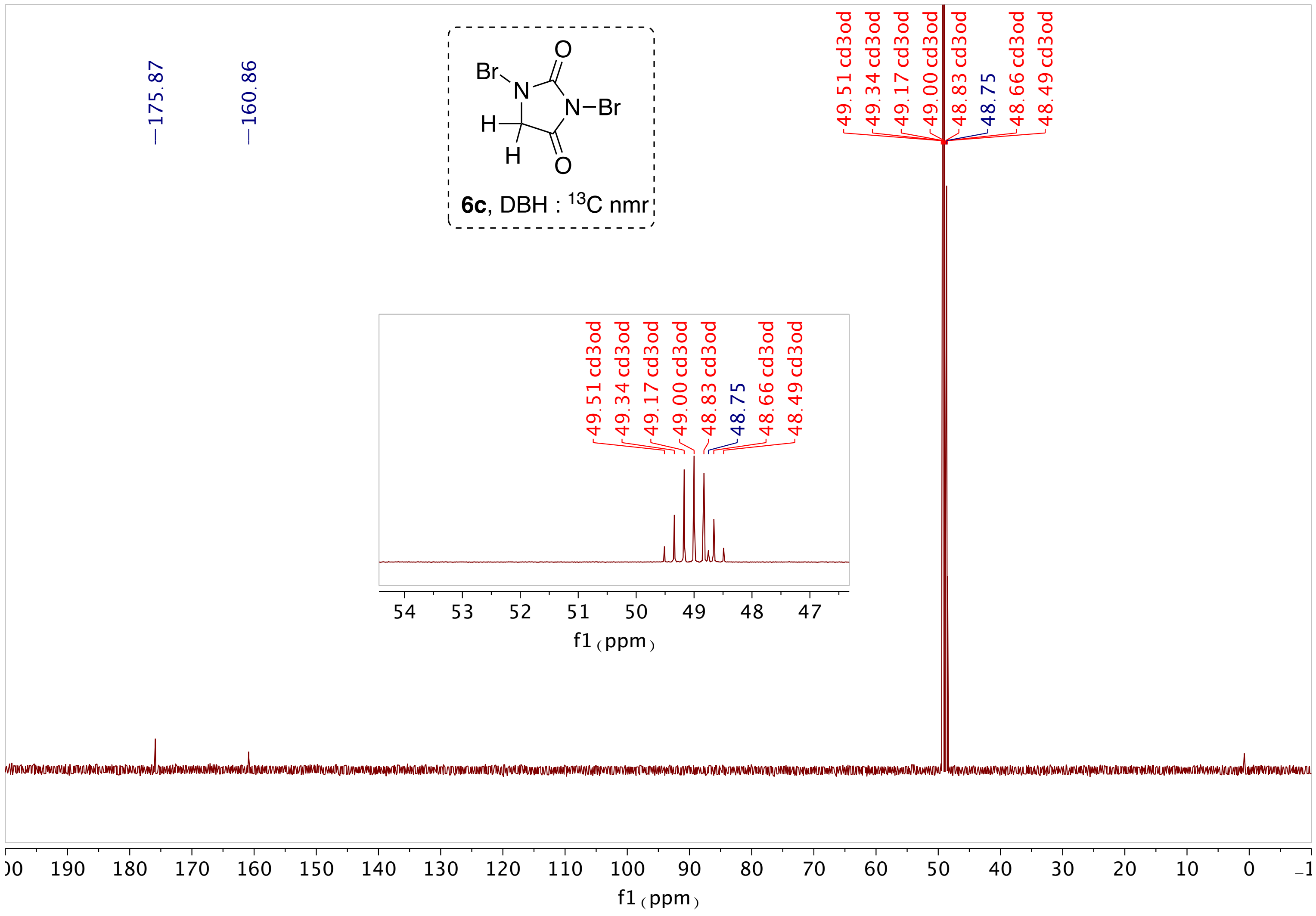

Figure S26: ^{1}H NMR of Compound **6d**: 1-bromopyrrolidine-2,5-dione -^{1}H NMR (500 MHz, $CDCl_3$)

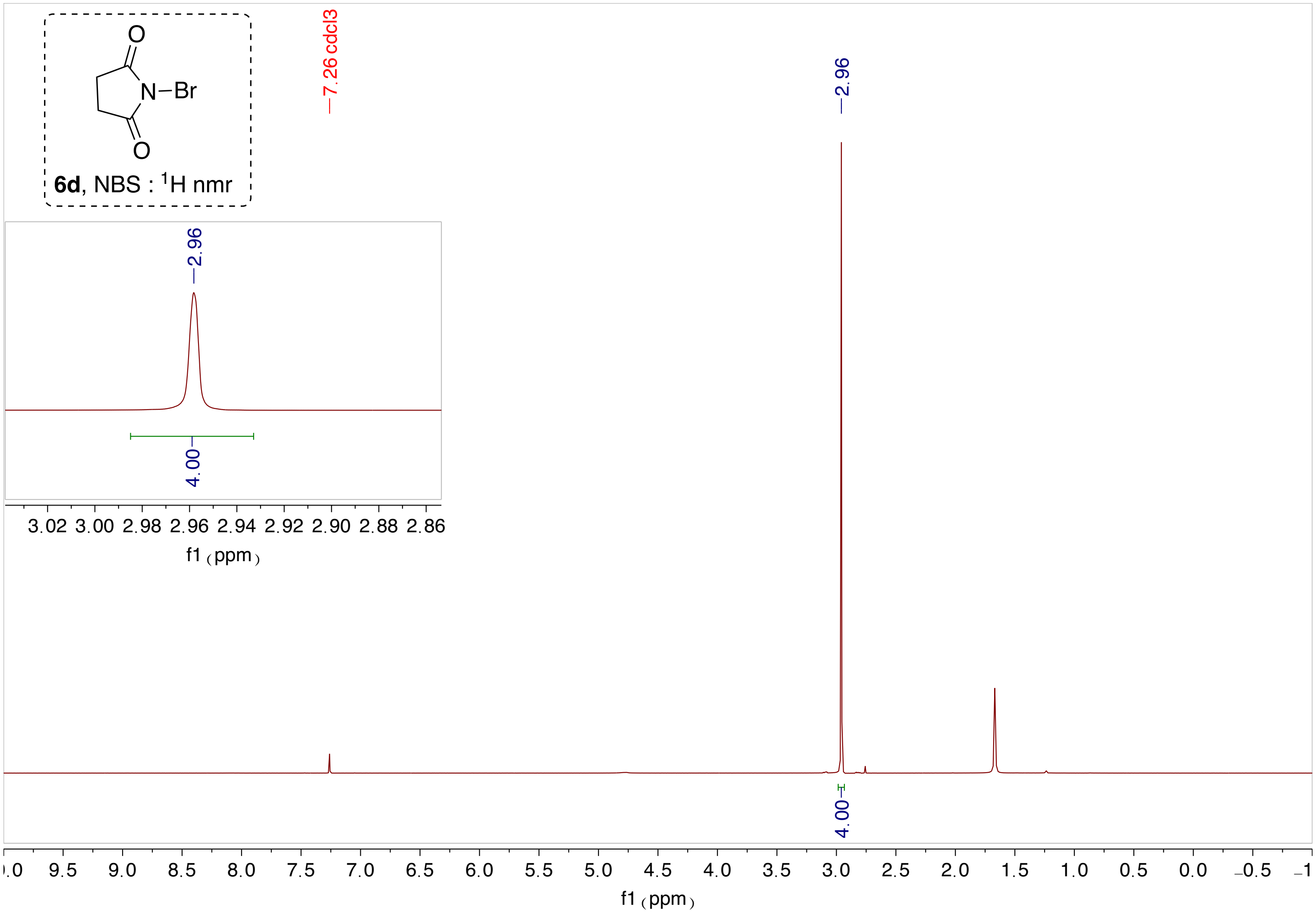

Figure S27: ^{13}C NMR of Compound **6d**: 1-bromopyrrolidine-2,5-dione -^{13}C NMR (126 MHz, $CDCl_3$)

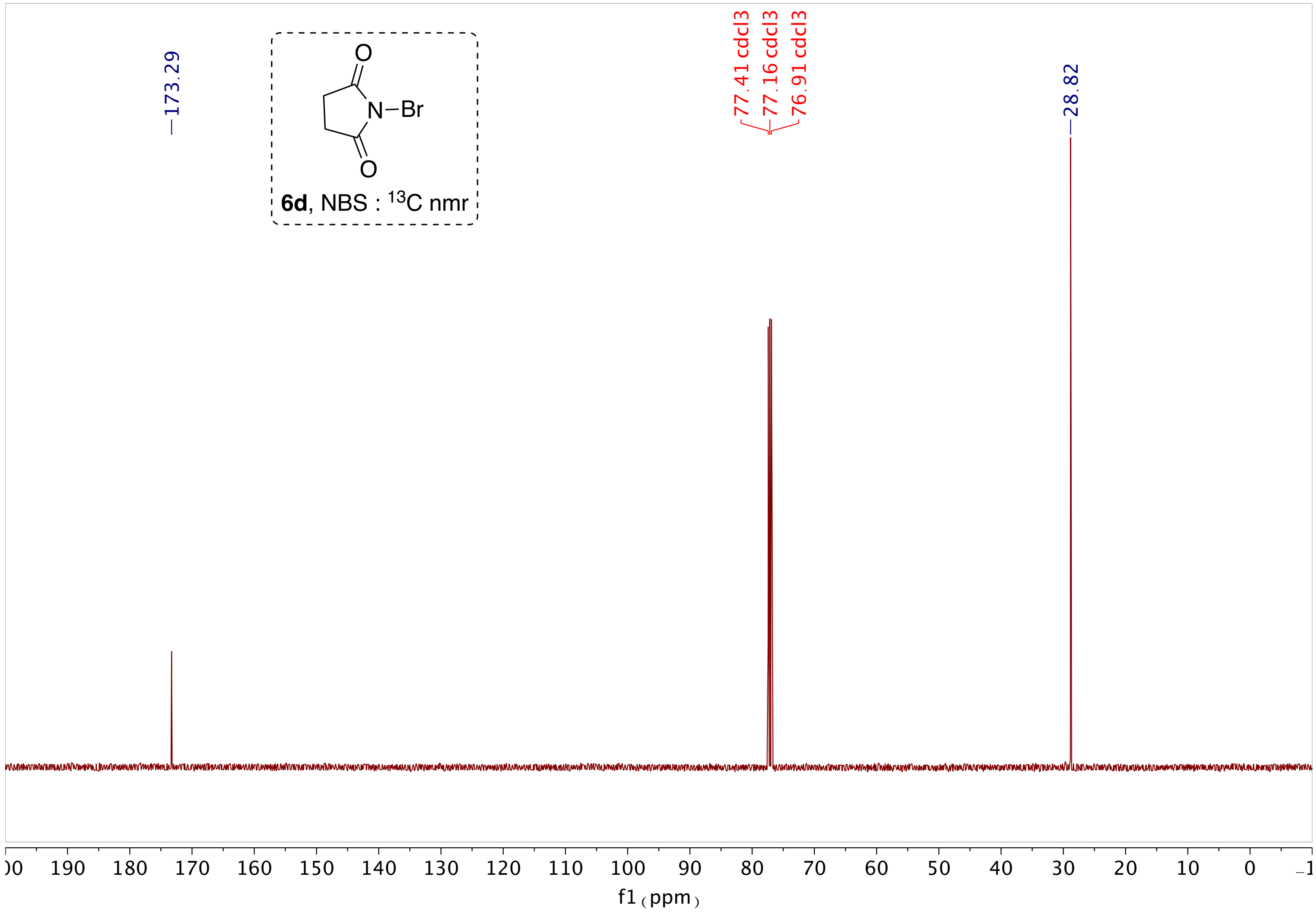

Figure S28: ^{1}H NMR of Compound **6e**: 2-bromoisoindoline-1,3-dione -^{1}H NMR (500 MHz, $CDCl_3$)

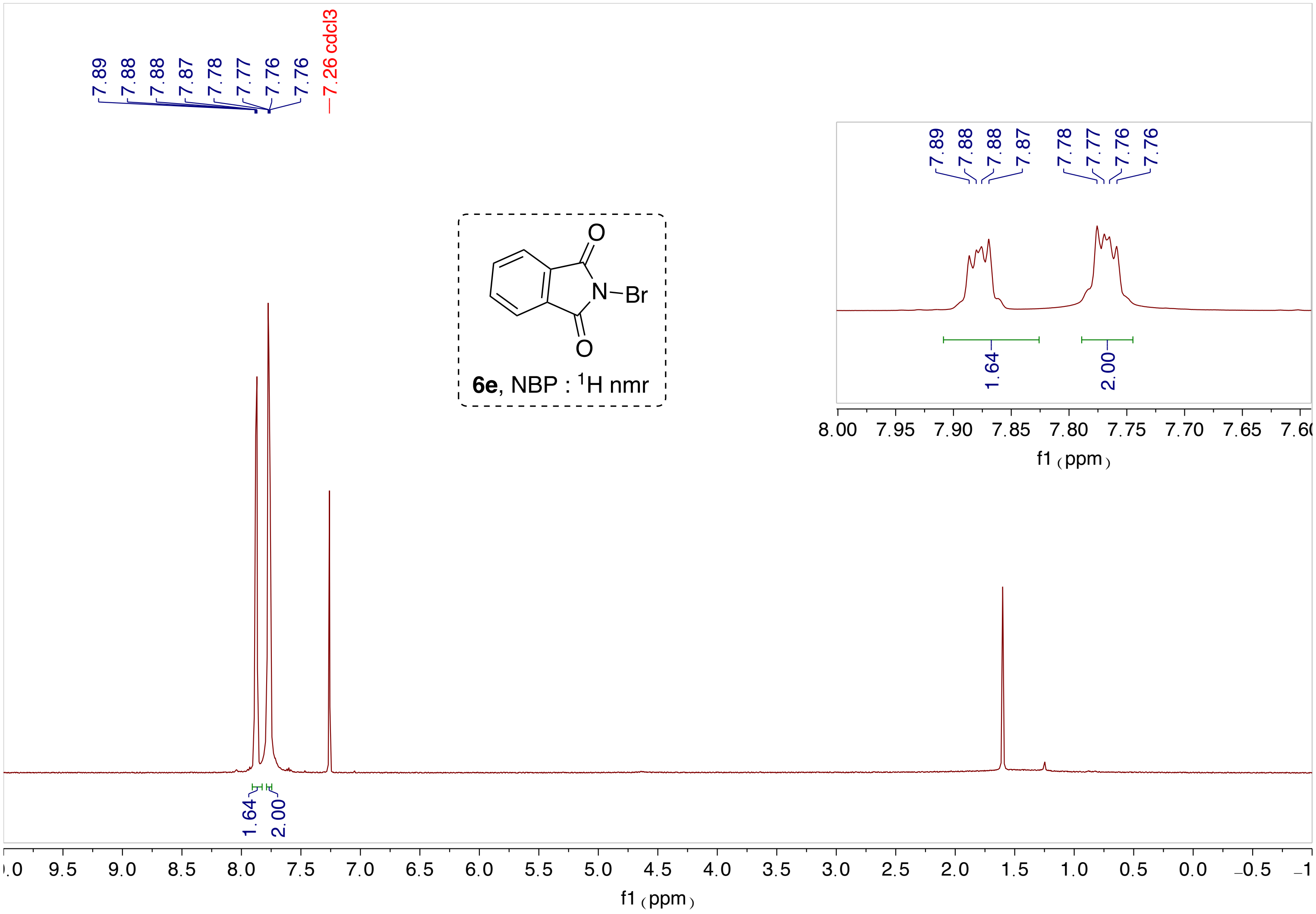

Figure S29: ^{13}C NMR of Compound **6e**: 2-bromoisoindoline-1,3-dione -^{13}C NMR (126 MHz, $CDCl_3$)

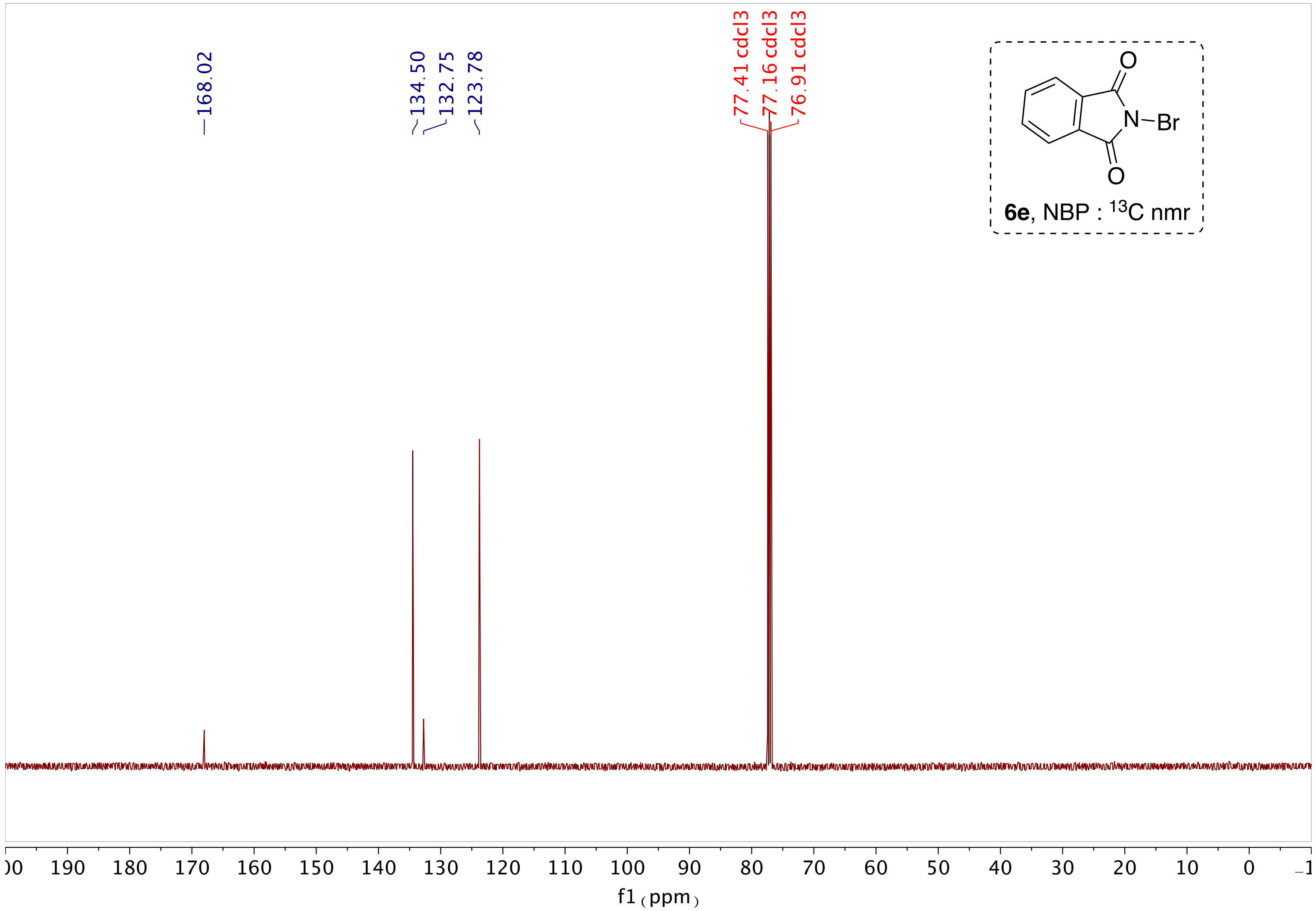

Figure S30: ^{1}H NMR of Compound **6f**: 1-bromopyrrolidin-2-one -^{1}H NMR (500 MHz, CD_3OD)

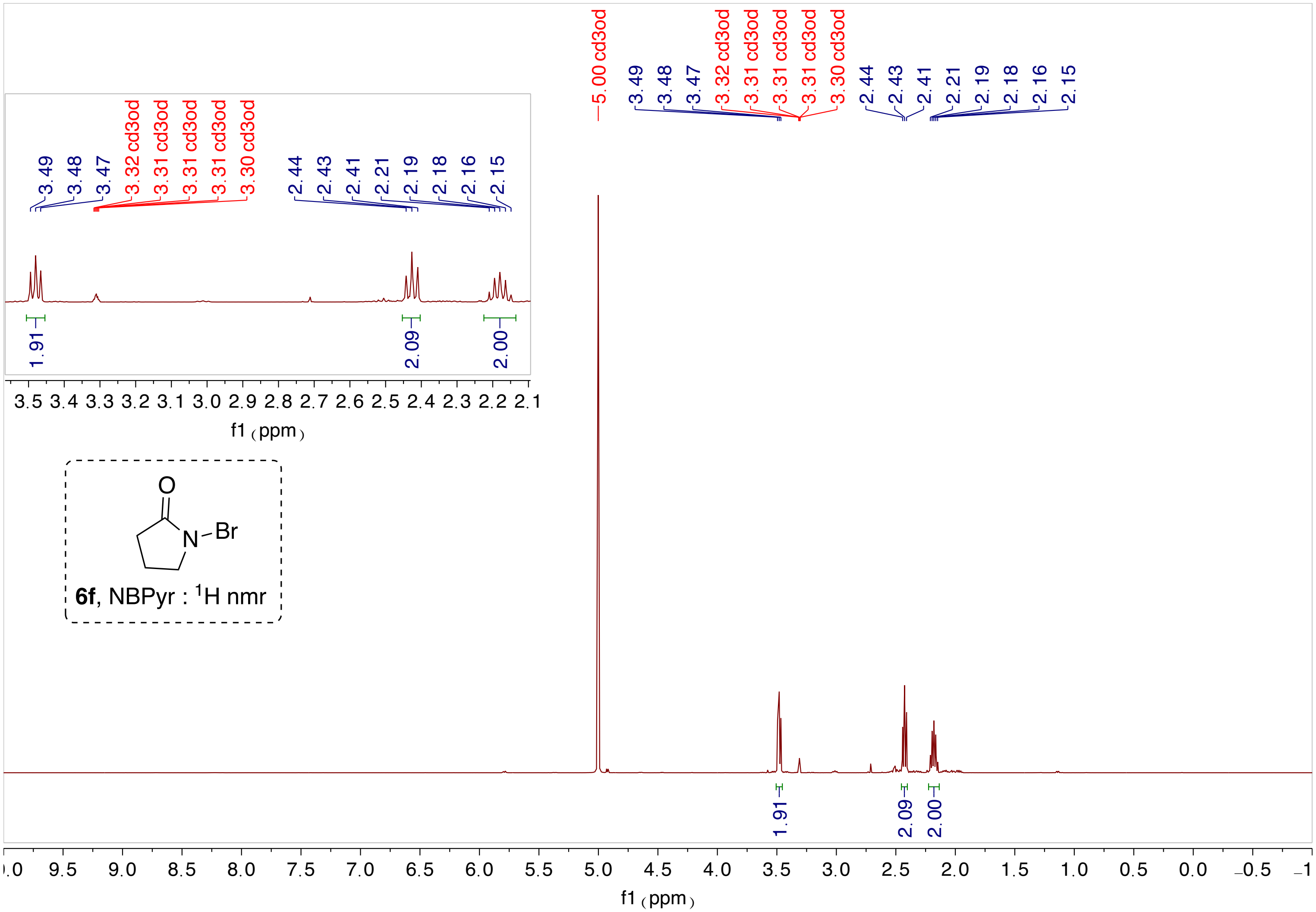

Figure S31: ^{13}C NMR of Compound **6f**: 1-bromopyrrolidin-2-one -^{13}C NMR (126 MHz, CD_3OD)

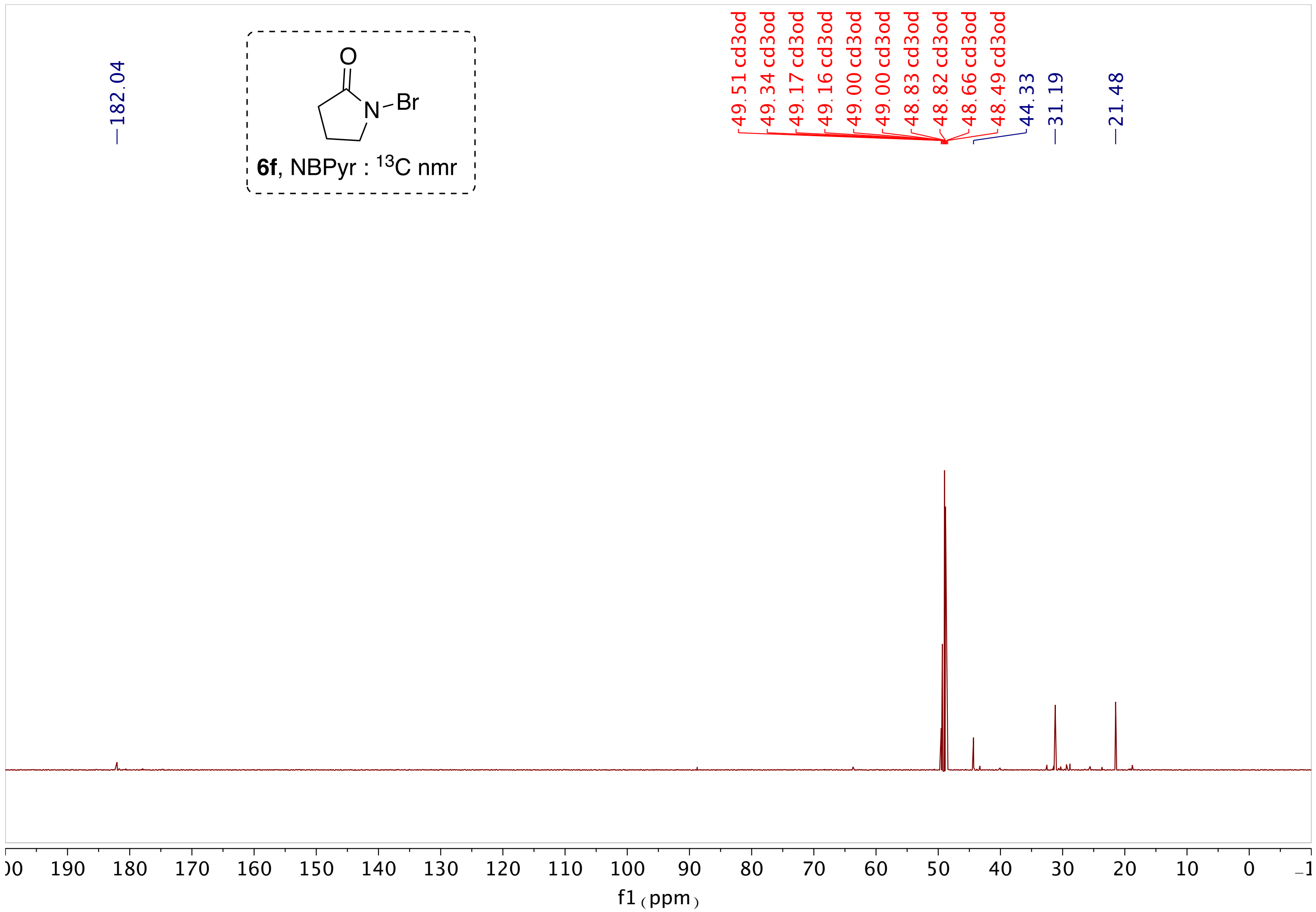

Figure S32: ^{1}H NMR of Compound **6g**: *N*-bromoacetamide -^{1}H NMR (500 MHz, $CDCl_3$)

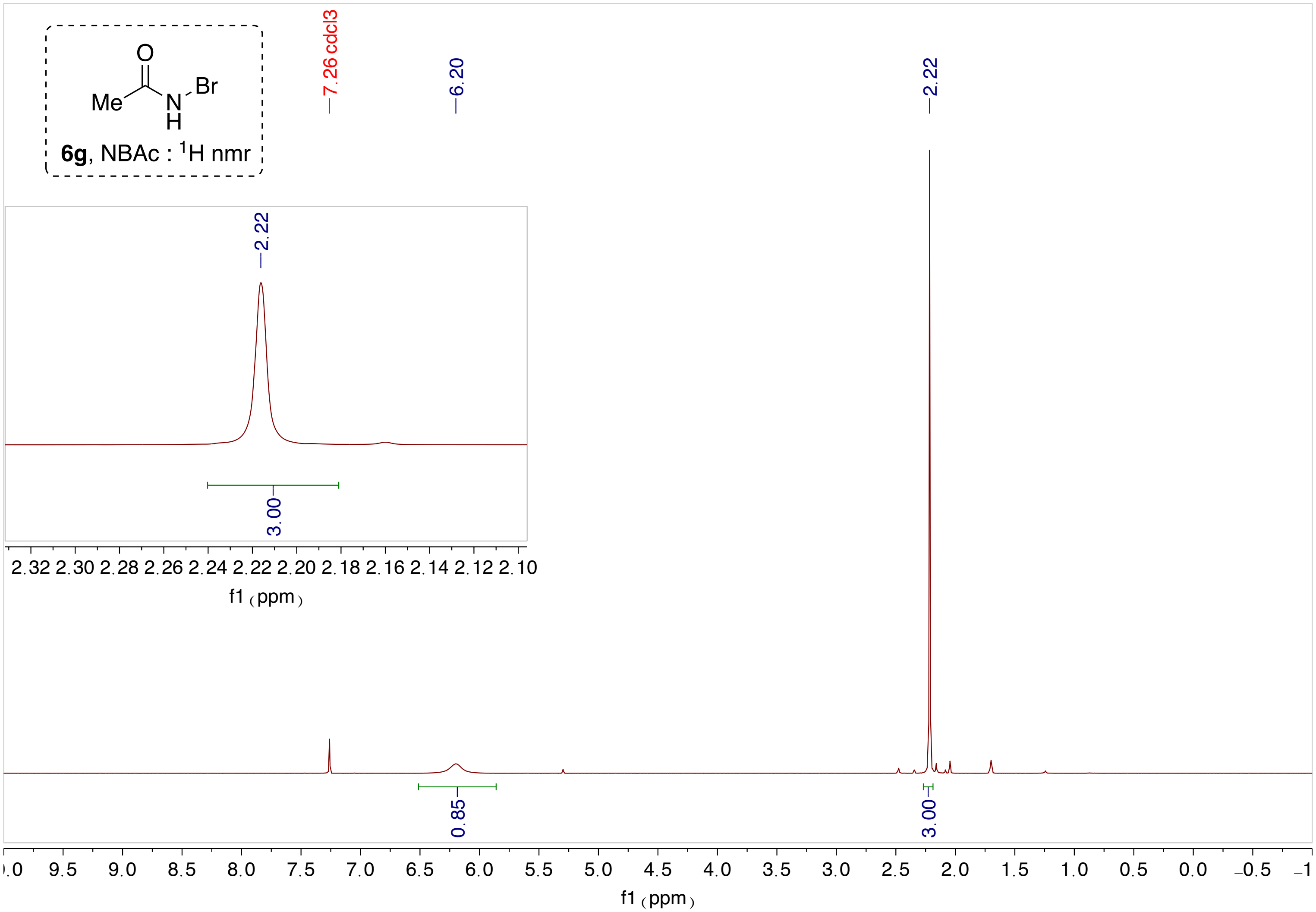

Figure S33: ^{13}C NMR of Compound **6g**: *N*-bromoacetamide -^{13}C NMR (126 MHz, CD_3CN)

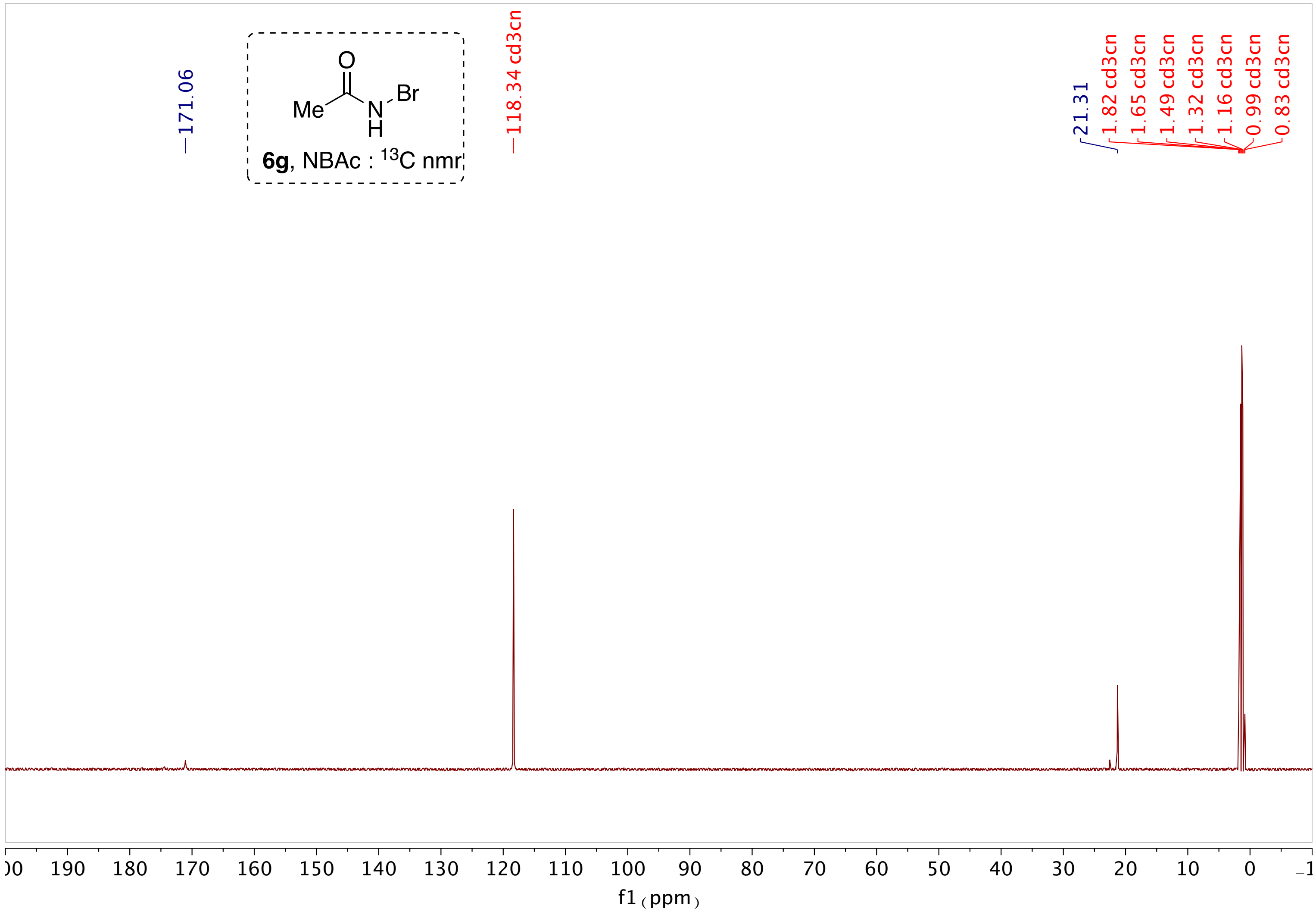

Figure S34: ^{13}C NMR of Compound **6h**: 1,3,5-tribromo-1,3,5-triazinane-2,4,6-trione -$^{13}C\{^{1}H\}$ (126 MHz, CD_3OD)

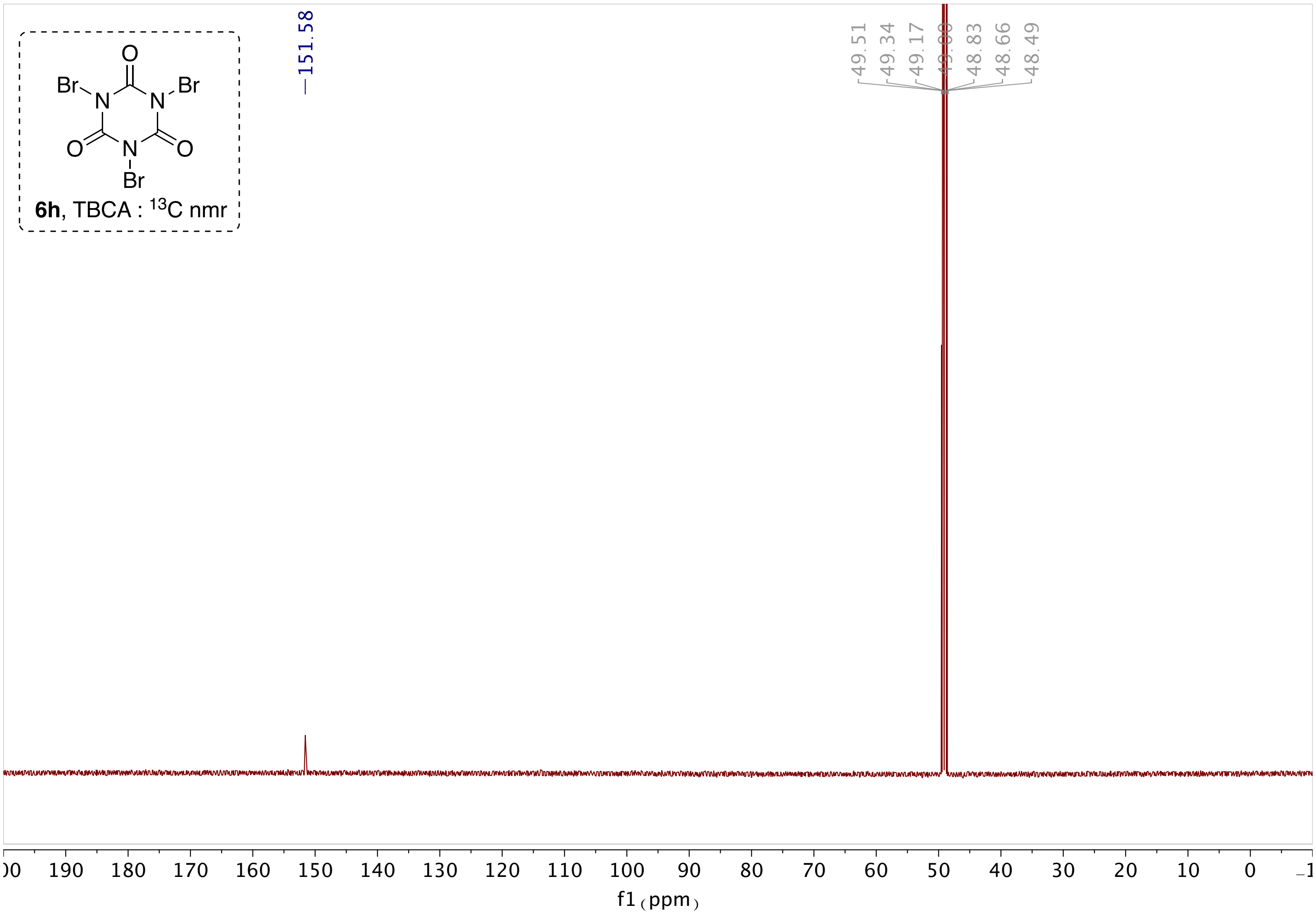

Figure S35: ^{1}H NMR of Compound **6i**: 2-bromobenzo[*d*]isothiazol-3(2*H*)-one 1,1-dioxide -^{1}H NMR (500 MHz, $CDCl_3$)

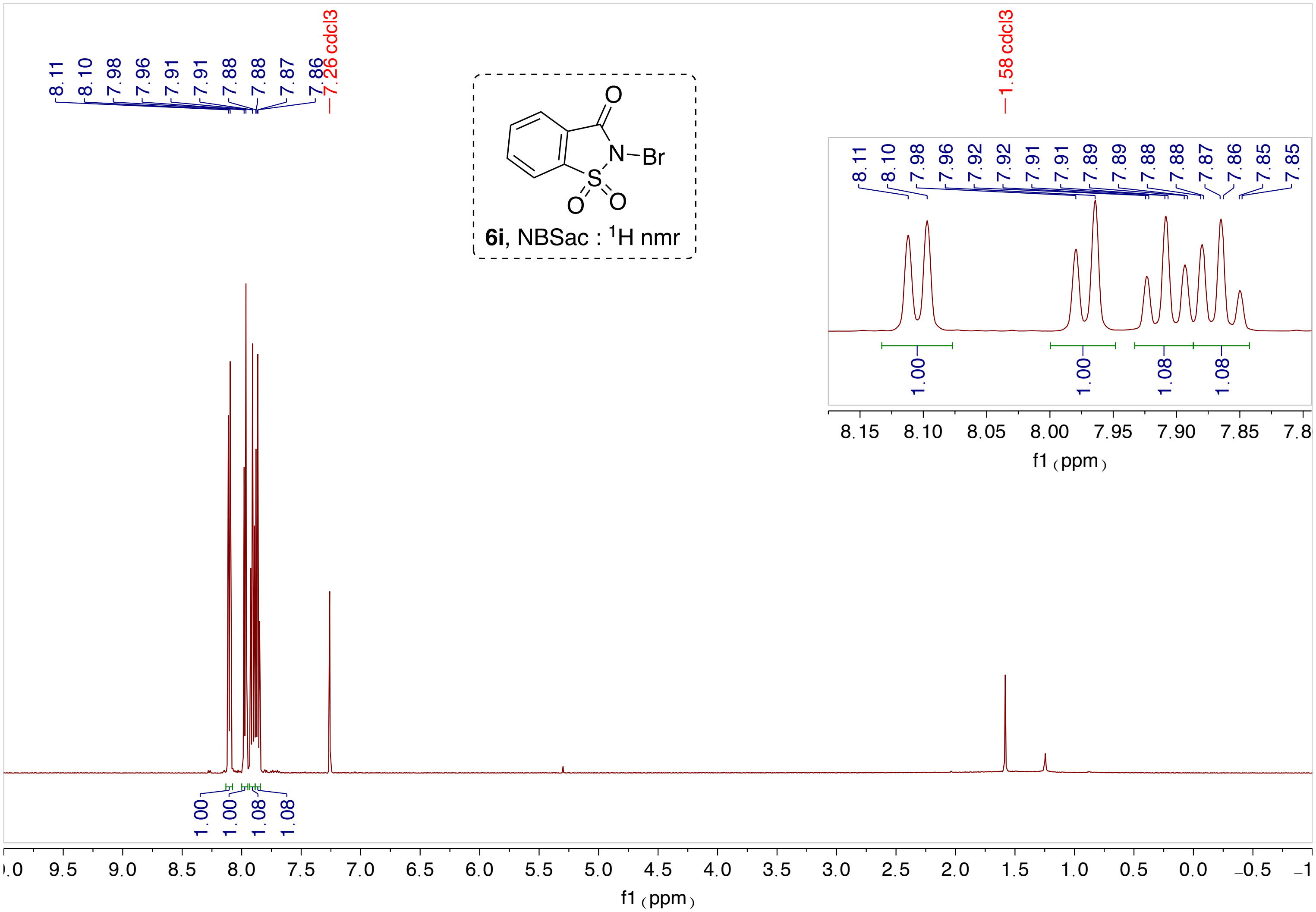

Figure S36: ^{13}C NMR of Compound **6i**: 2-bromobenzo[*d*]isothiazol-3(2*H*)-one 1,1-dioxide -^{13}C NMR (126 MHz, $CDCl_3$)

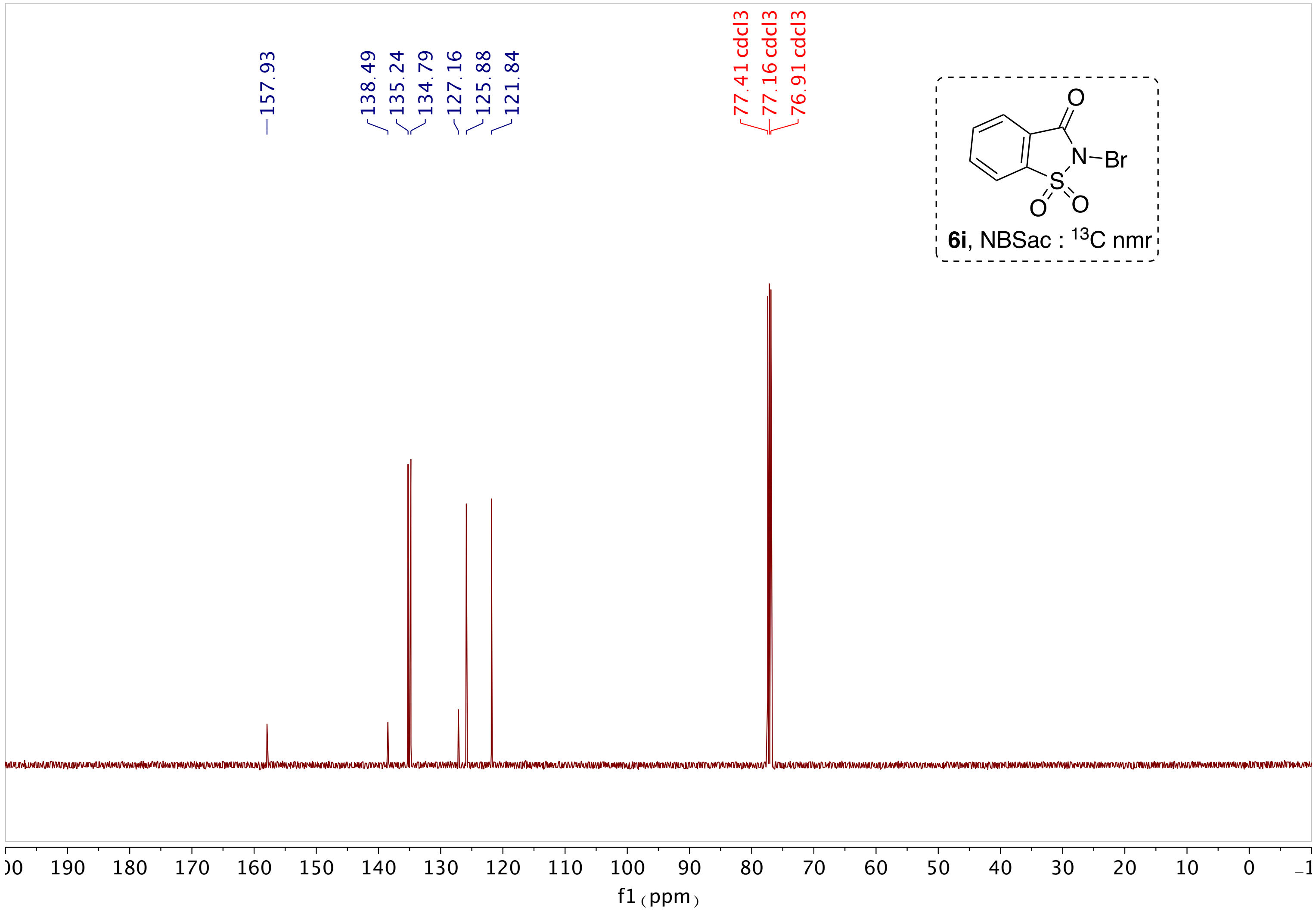

Figure S37: ^{1}H NMR of Compound **6j**: *N*,*N*-dibromo-4-methylbenzenesulfonamide-^{1}H NMR (500 MHz, $CDCl_3$)

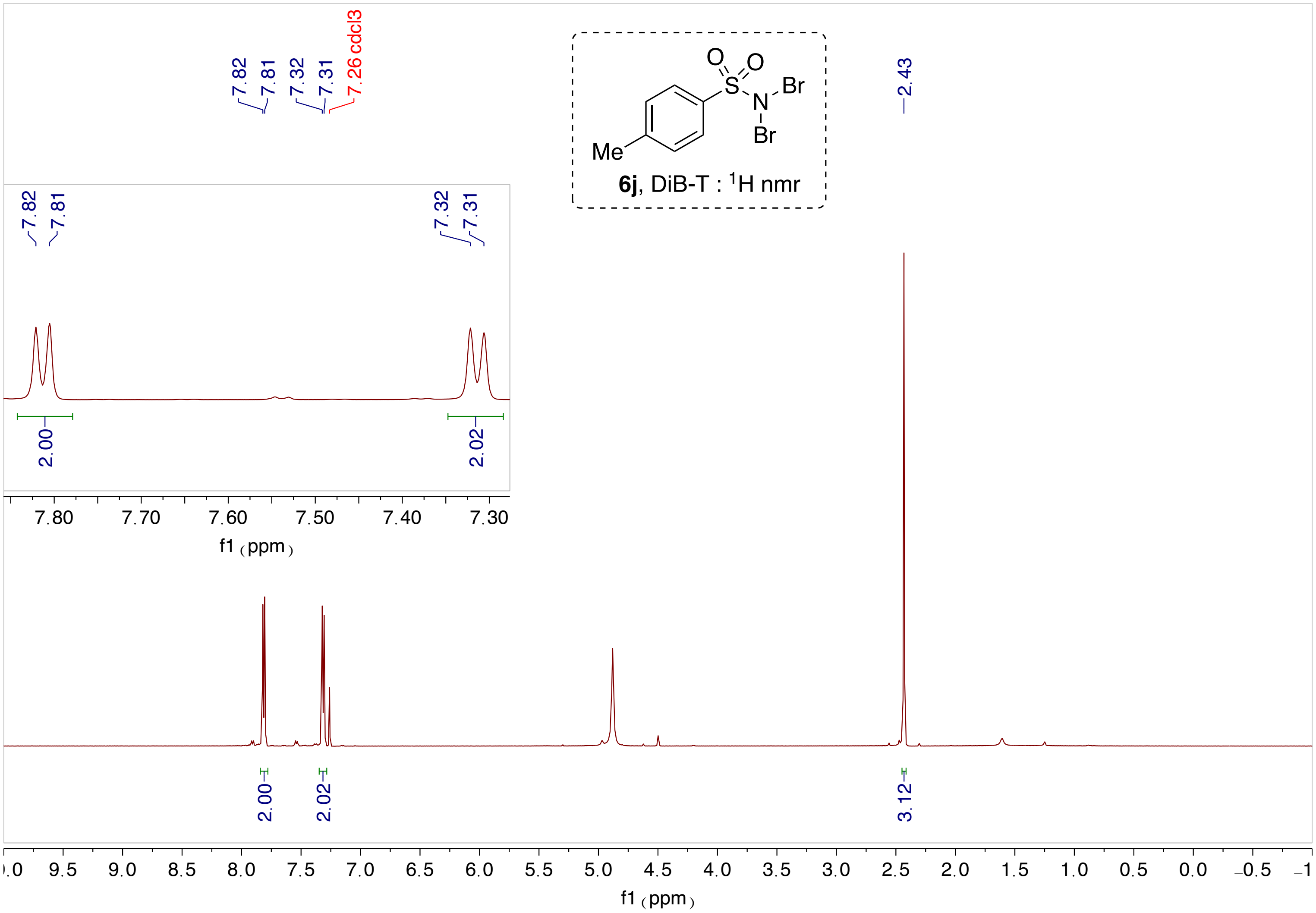

Figure S38: ^{13}C NMR of Compound **6j**: *N*,*N*-dibromo-4-methylbenzenesulfonamide-^{13}C NMR (126 MHz, $CDCl_3$)

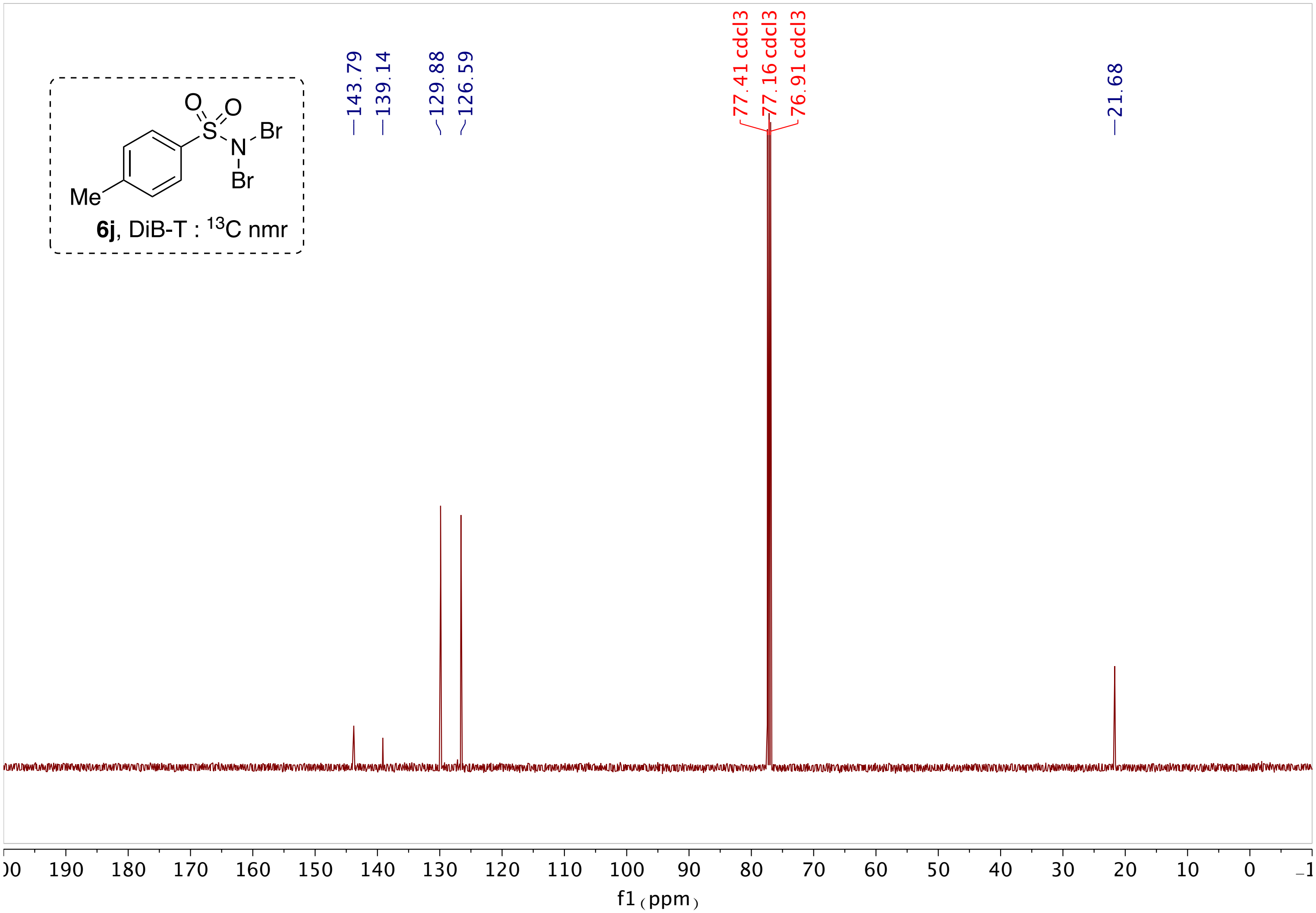

Figure S39: ^{1}H NMR of Compound **7a**: 1,3-diiodo-5,5-dimethylimidazolidine-2,4-dione-^{1}H NMR (500 MHz, CD_3OD)

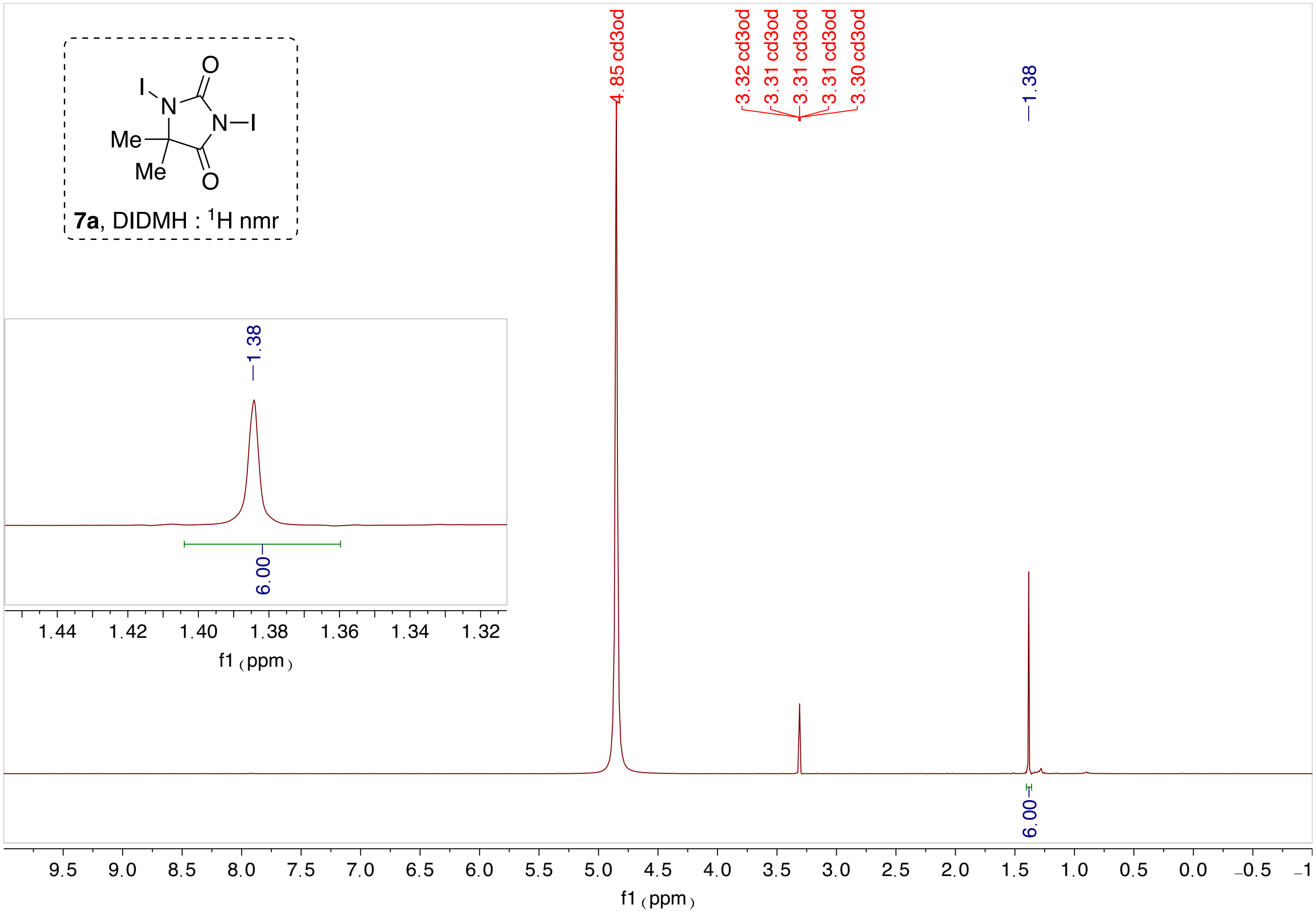

Figure S40: ^{13}C NMR of Compound **7a**: 1,3-diiodo-5,5-dimethylimidazolidine-2,4-dione-^{13}C NMR (126 MHz, CD_3OD)

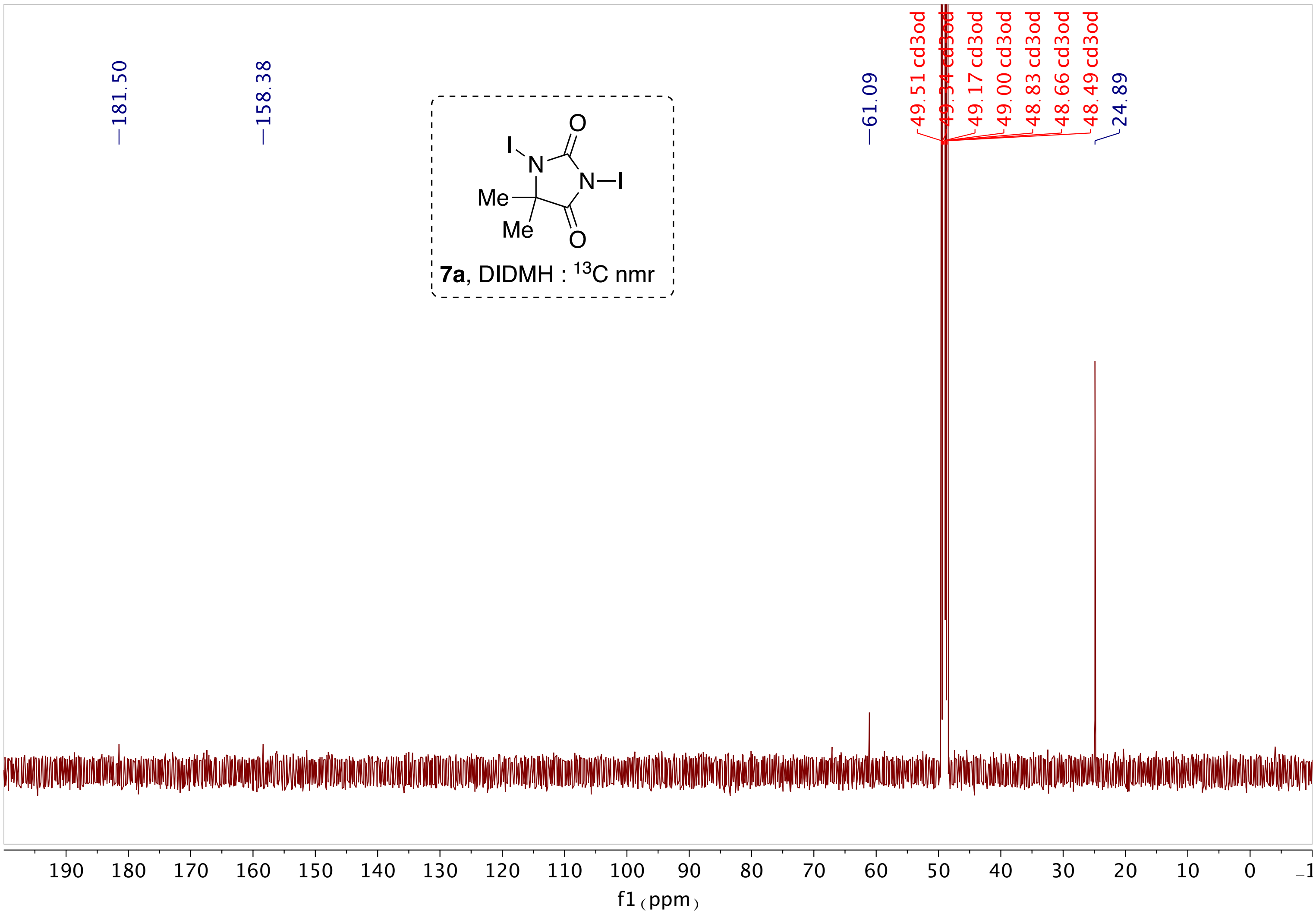

Figure S41: ^{1}H NMR of Compound **7b**: 1,3-diiodo-5,5-diphenylimidazolidine-2,4-dione-^{1}H NMR (500 MHz, CD_3OD)

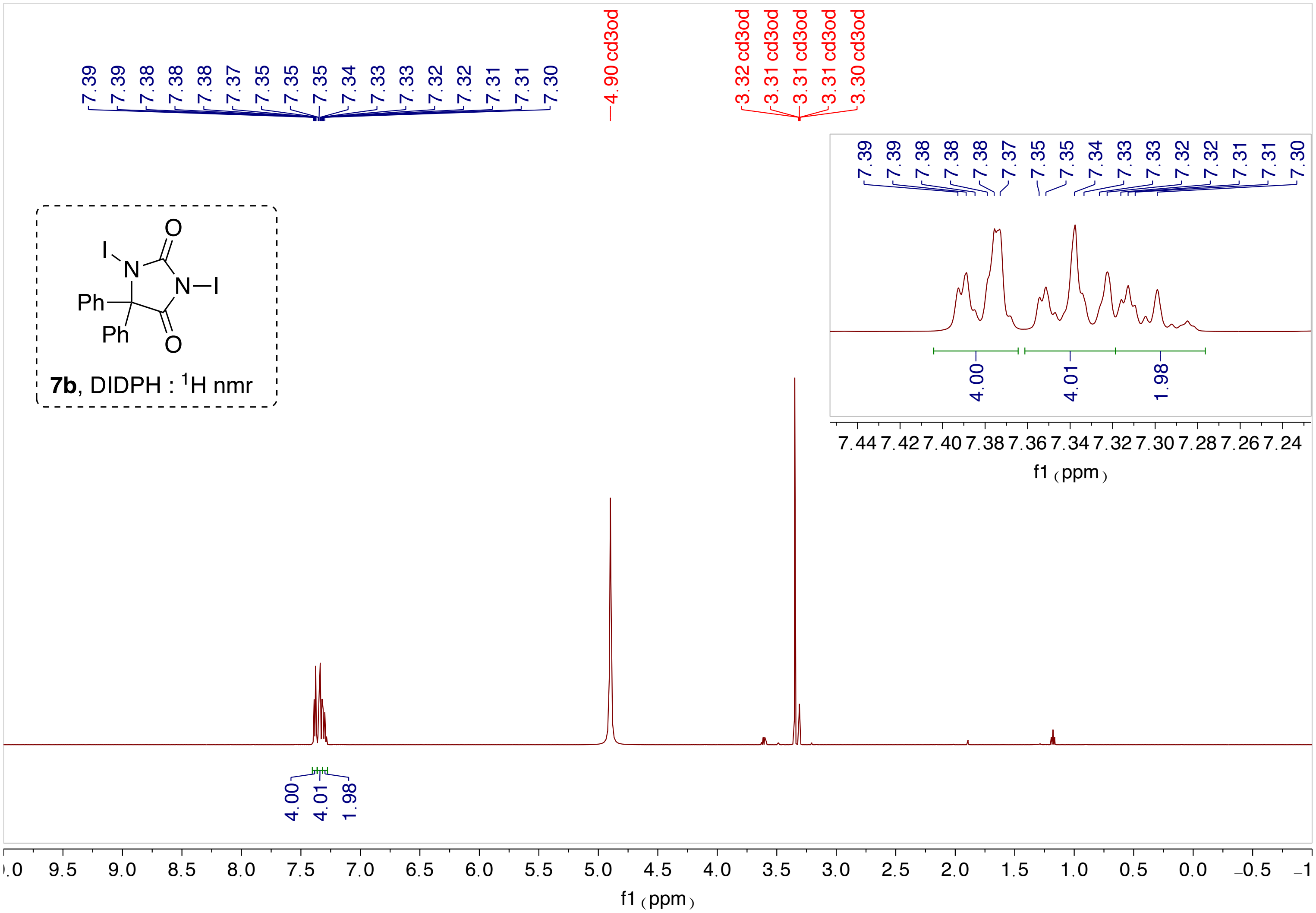

Figure S42: ^{13}C NMR of Compound **7b**: 1,3-diiodo-5,5-diphenylimidazolidine-2,4-dione-^{13}C NMR (126 MHz, CD_3OD)

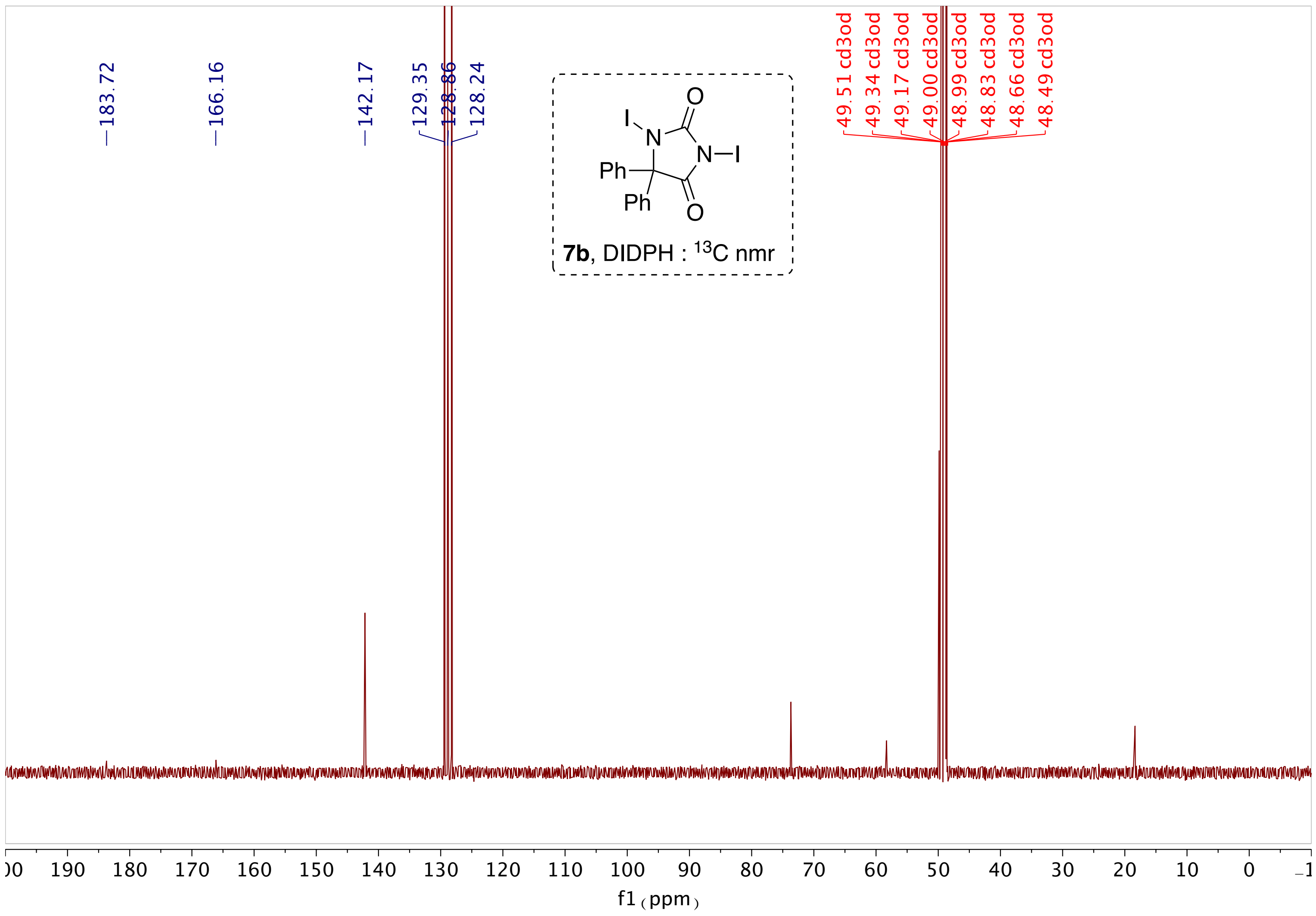

Figure S43: ^{1}H NMR of Compound **7c**: 1,3-diiodoimidazolidine-2,4-dione -^{1}H NMR (500 MHz, CD_3OD)

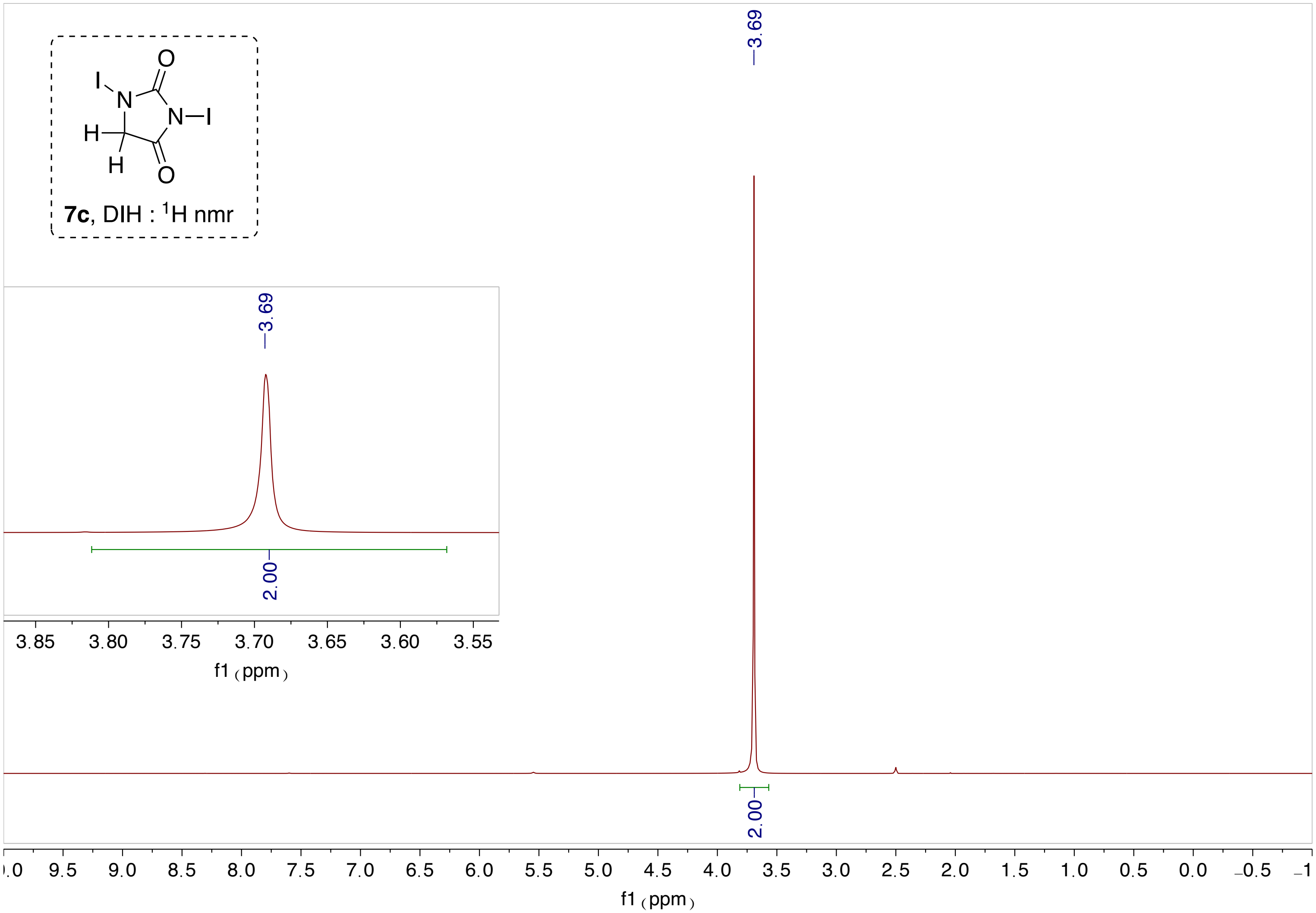

Figure S44: ^{13}C NMR of Compound **7c**: 1,3-diiodoimidazolidine-2,4-dione -^{13}C NMR (126 MHz, CD_3OD)

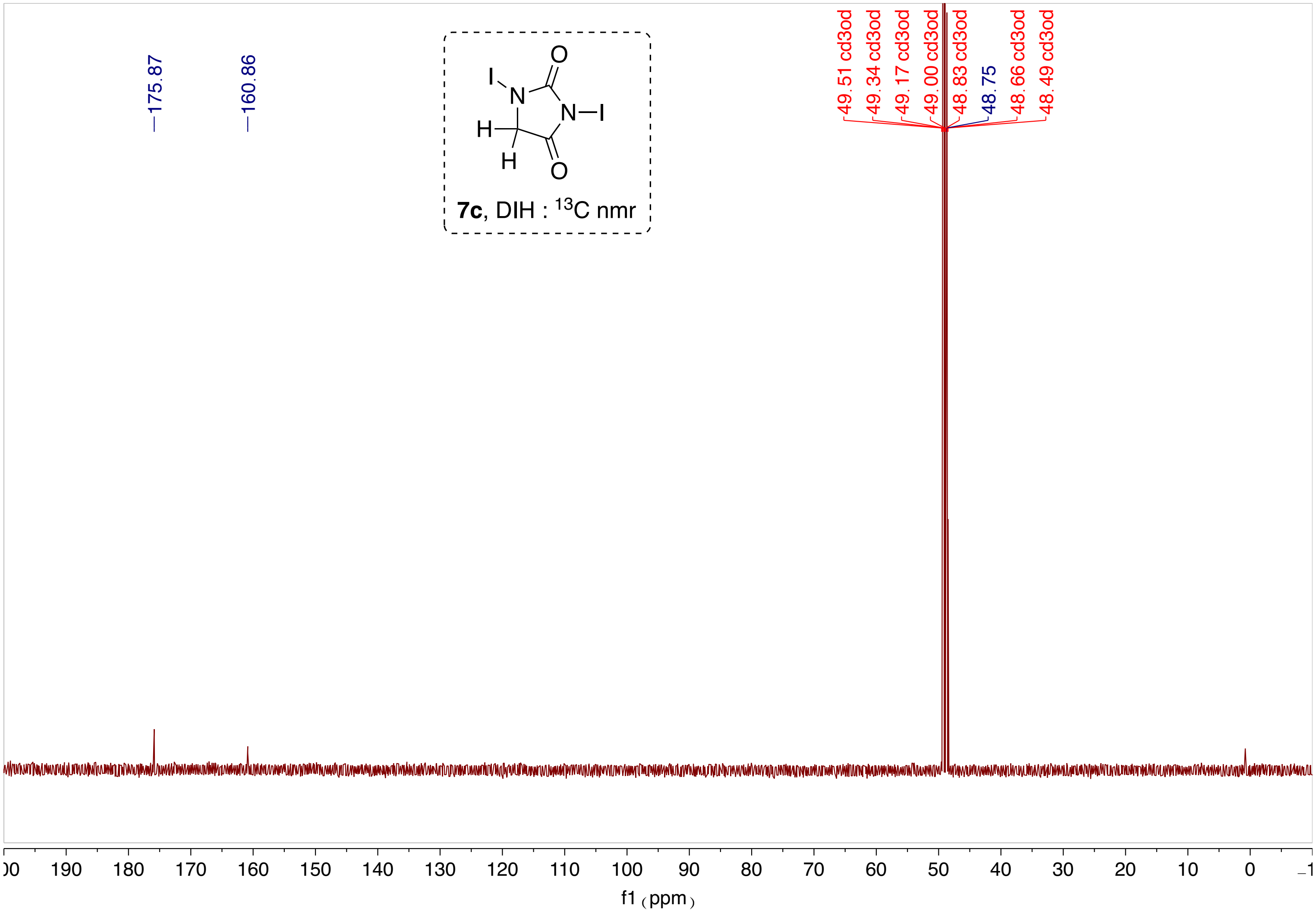

Figure S45: ^{1}H NMR of Compound **7d**: 1-iodopyrrolidine-2,5-dione -^{1}H NMR (500 MHz, $CDCl_3$)

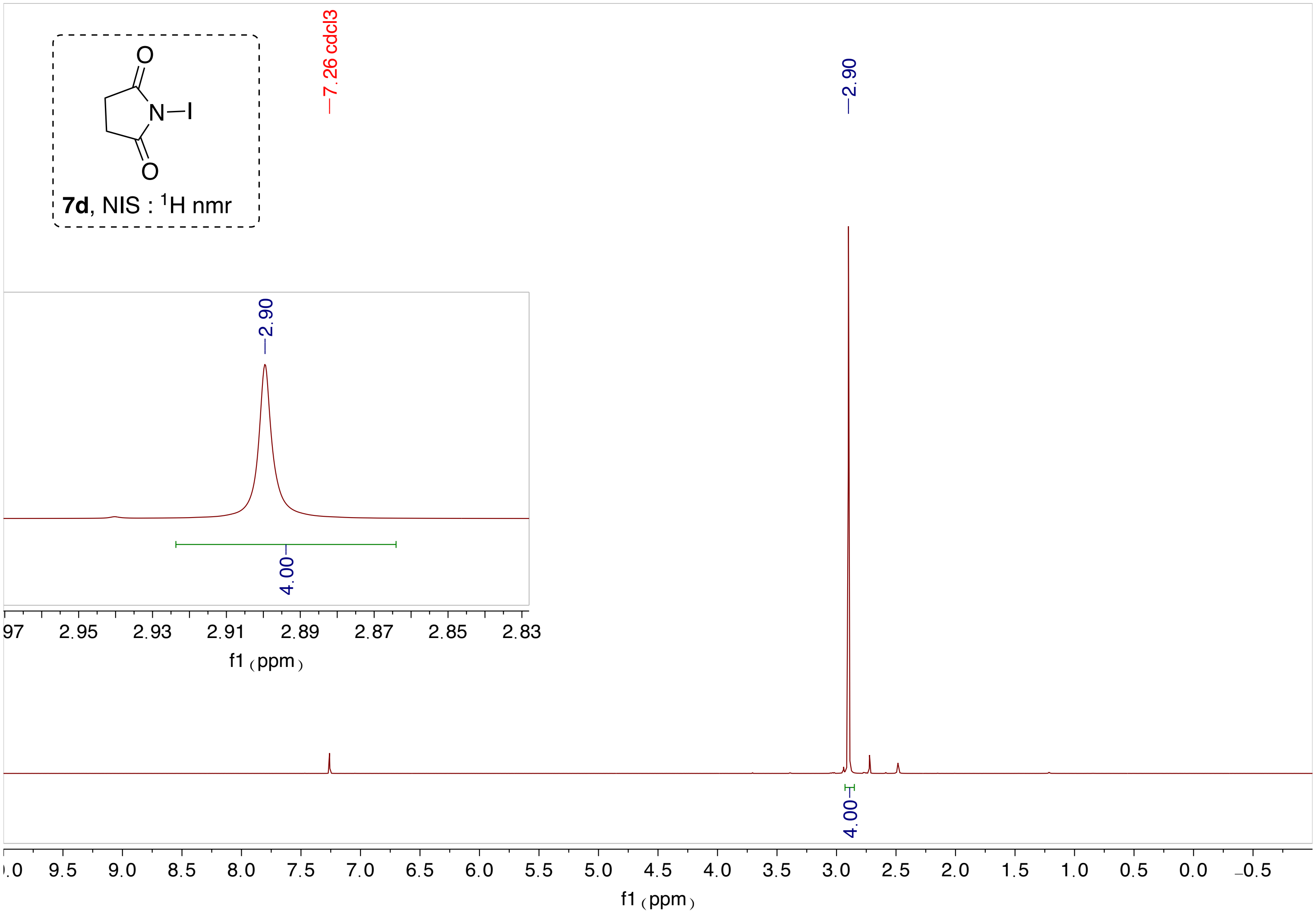

Figure S46: ^{13}C NMR of Compound **7d**: 1-iodopyrrolidine-2,5-dione -^{13}C NMR (126 MHz, CDCl$_3$)

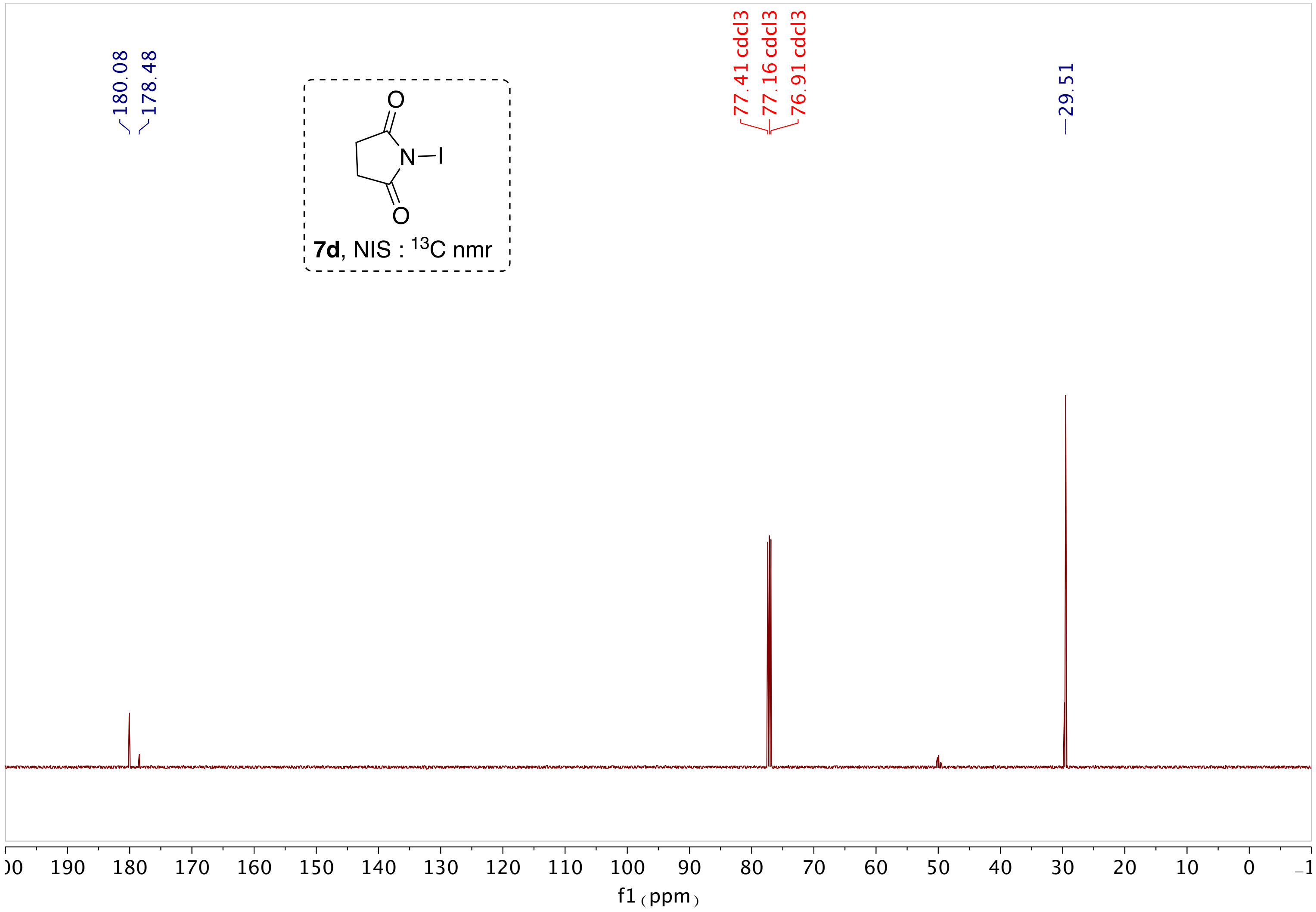

Figure S47: ^{1}H NMR of Compound **7e**: 2-iodoisoindoline-1,3-dione -^{1}H NMR (500 MHz, $CDCl_3$)

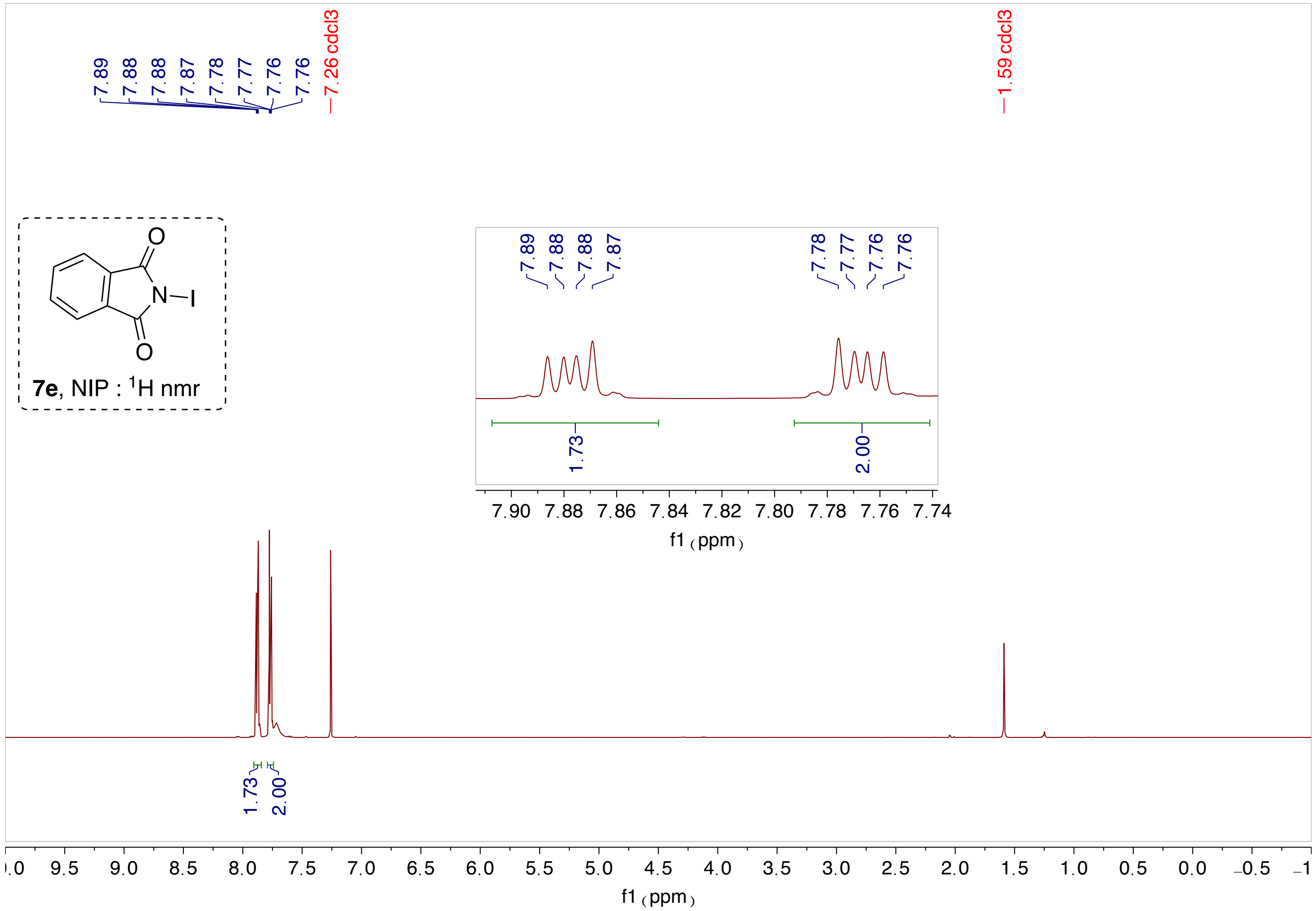

Figure S48: ^{13}C NMR of Compound **7e**: 2-iodoisoindoline-1,3-dione -^{13}C NMR (126 MHz, $CDCl_3$)

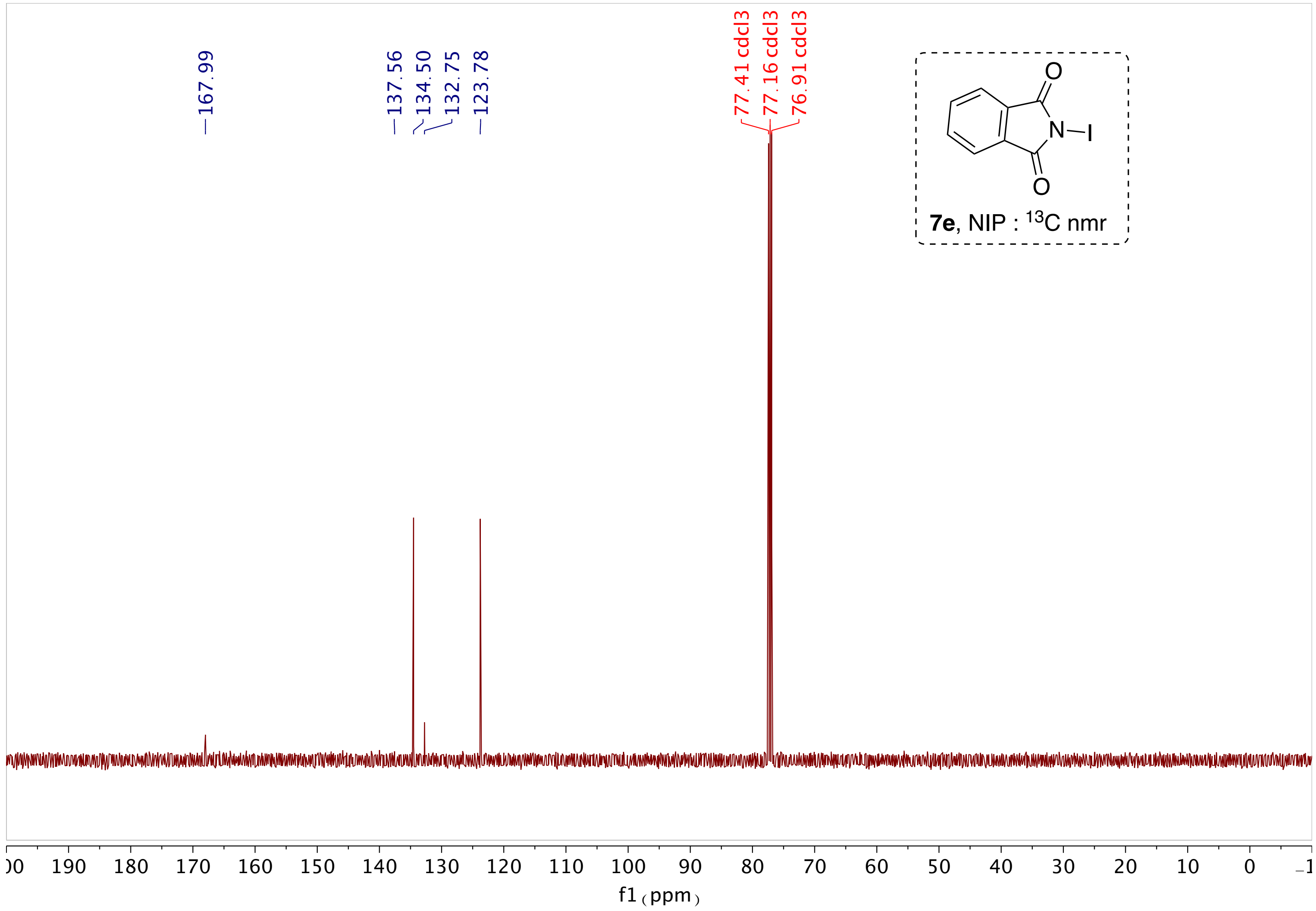

Figure S49: ^{1}H NMR of Compound **7f**: 1-iodopyrrolidin-2-one -^{1}H NMR (500 MHz, CD_3OD)

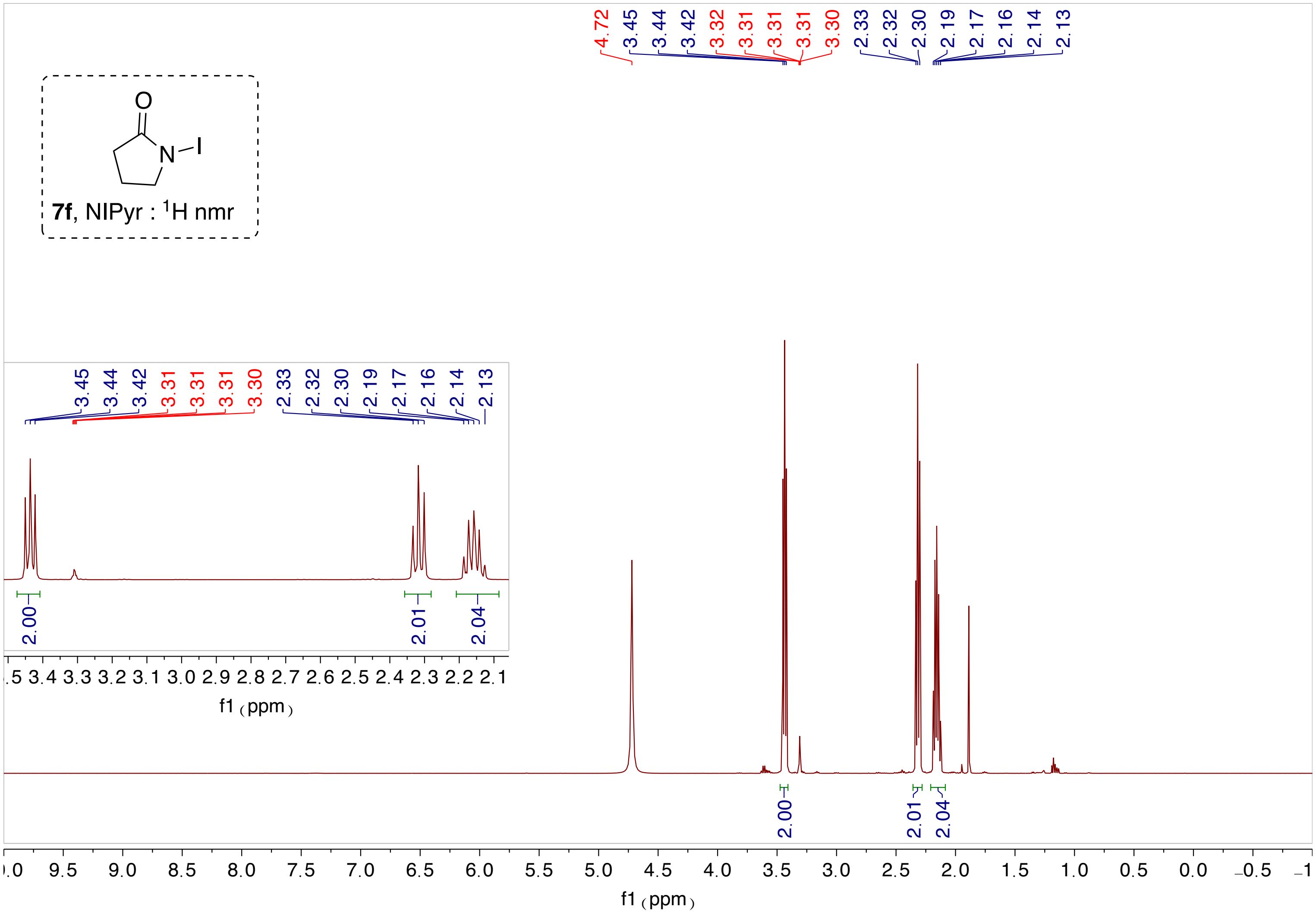

Figure S50: ^{13}C NMR of Compound **7f**: 1-iodopyrrolidin-2-one -^{13}C NMR (126 MHz, CD_3OD)

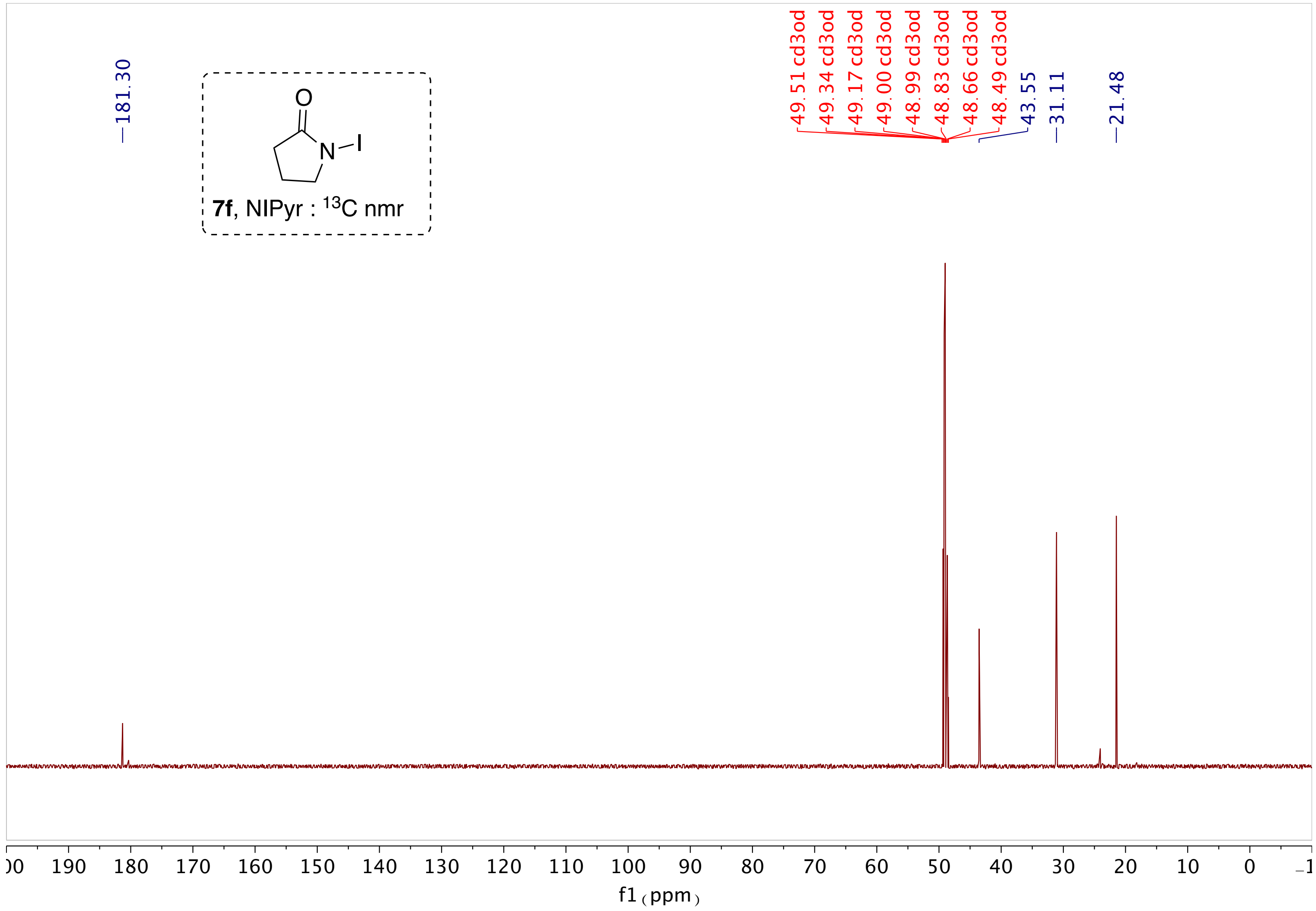

Figure S51: ^{1}H NMR of Compound **7g**: *N*-iodoacetamide -^{1}H NMR (500 MHz, CD_3OD)

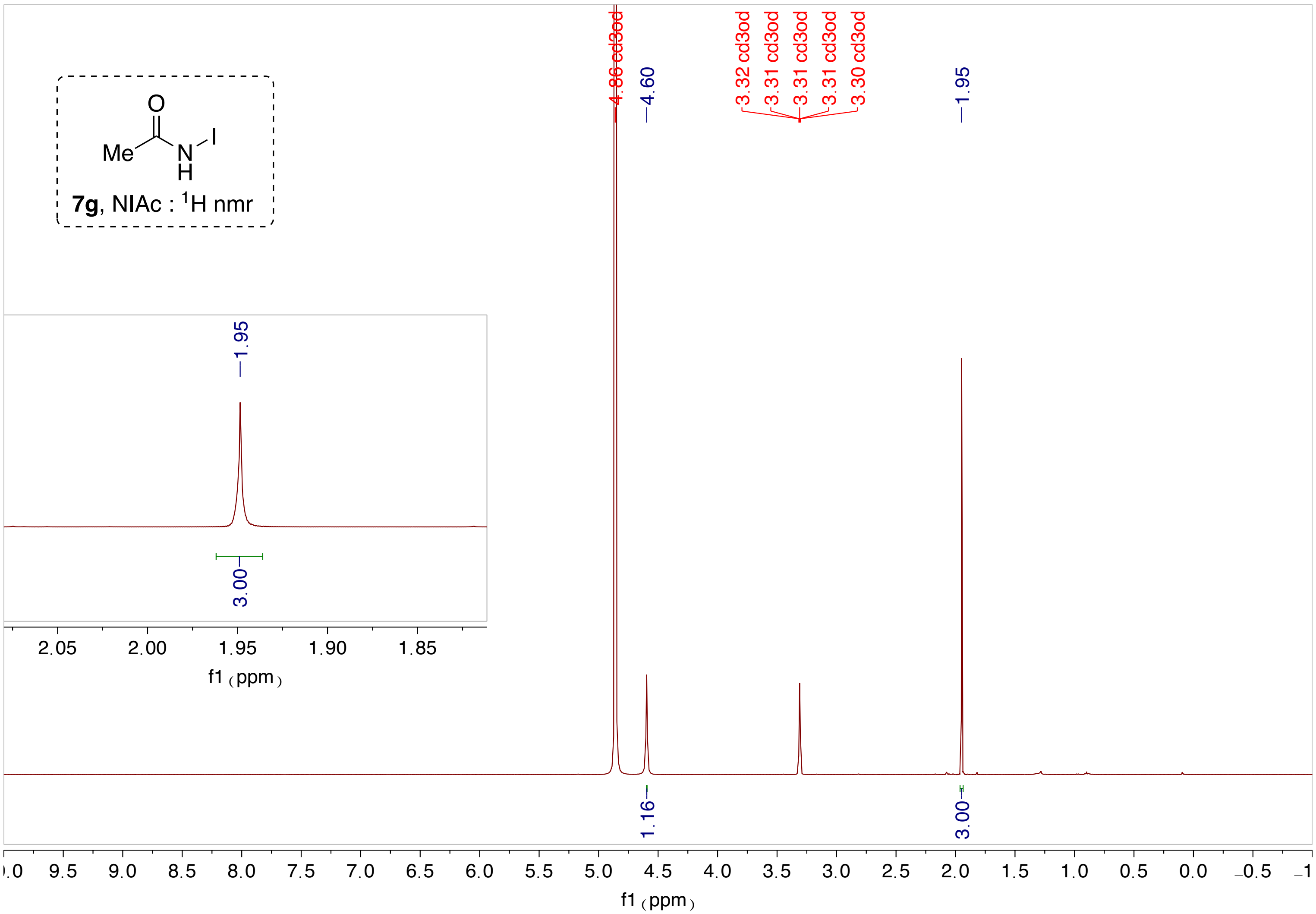

Figure S52: ^{13}C NMR of Compound **7g**: *N*-iodoacetamide -^{13}C NMR (126 MHz, CD_3OD)

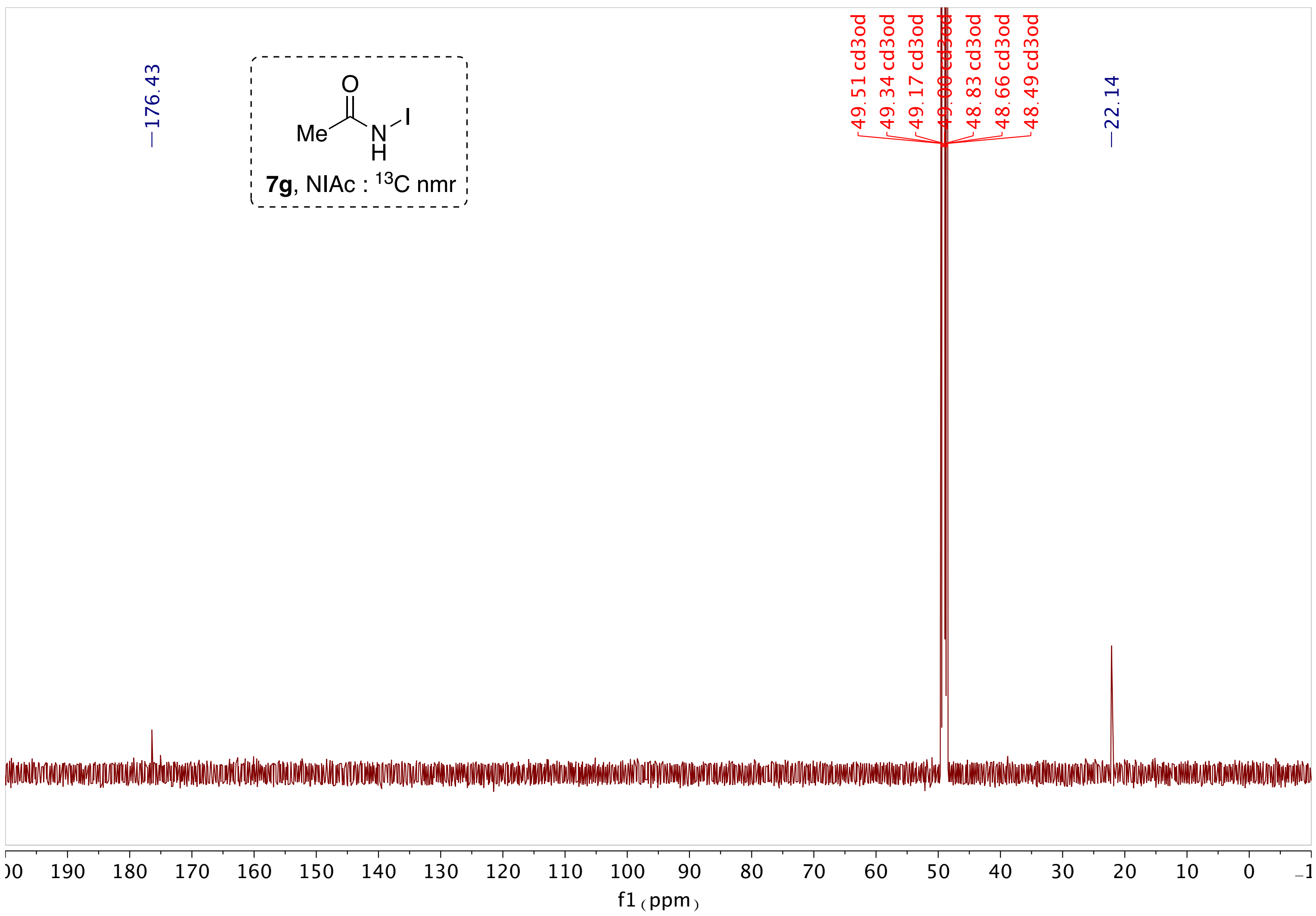

Figure S53: ^{13}C NMR of Compound **7h**: 1,3,5-triiodo-1,3,5-triazinane-2,4,6-trione -^{13}C{^{1}H} (126 MHz, D_6-DMSO)

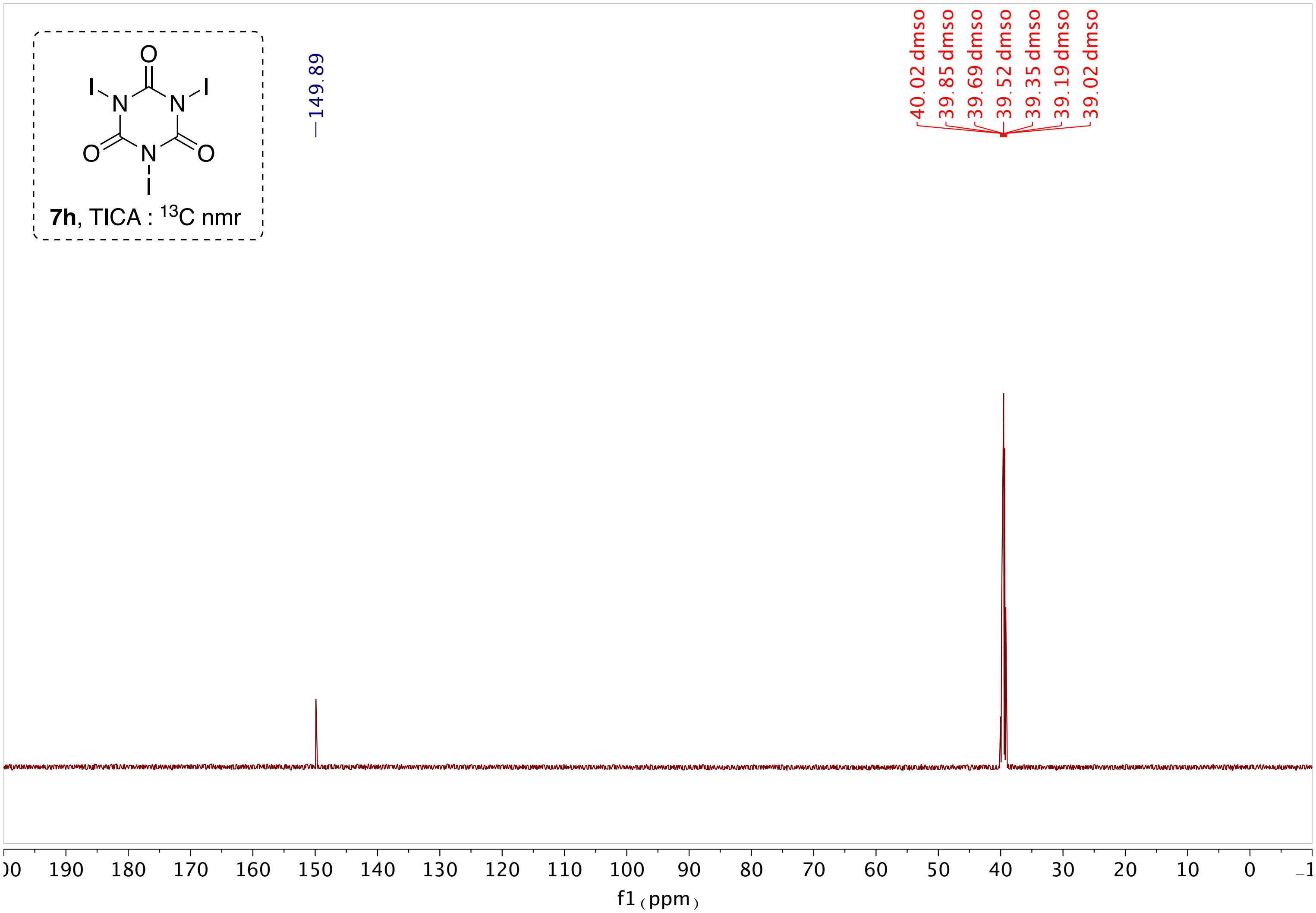

Figure S54: ^{1}H NMR of Compound **7i**: 2-iodobenzo[*d*]isothiazol-3(2*H*)-one 1,1-dioxide -^{1}H NMR (500 MHz, CD_3OD)

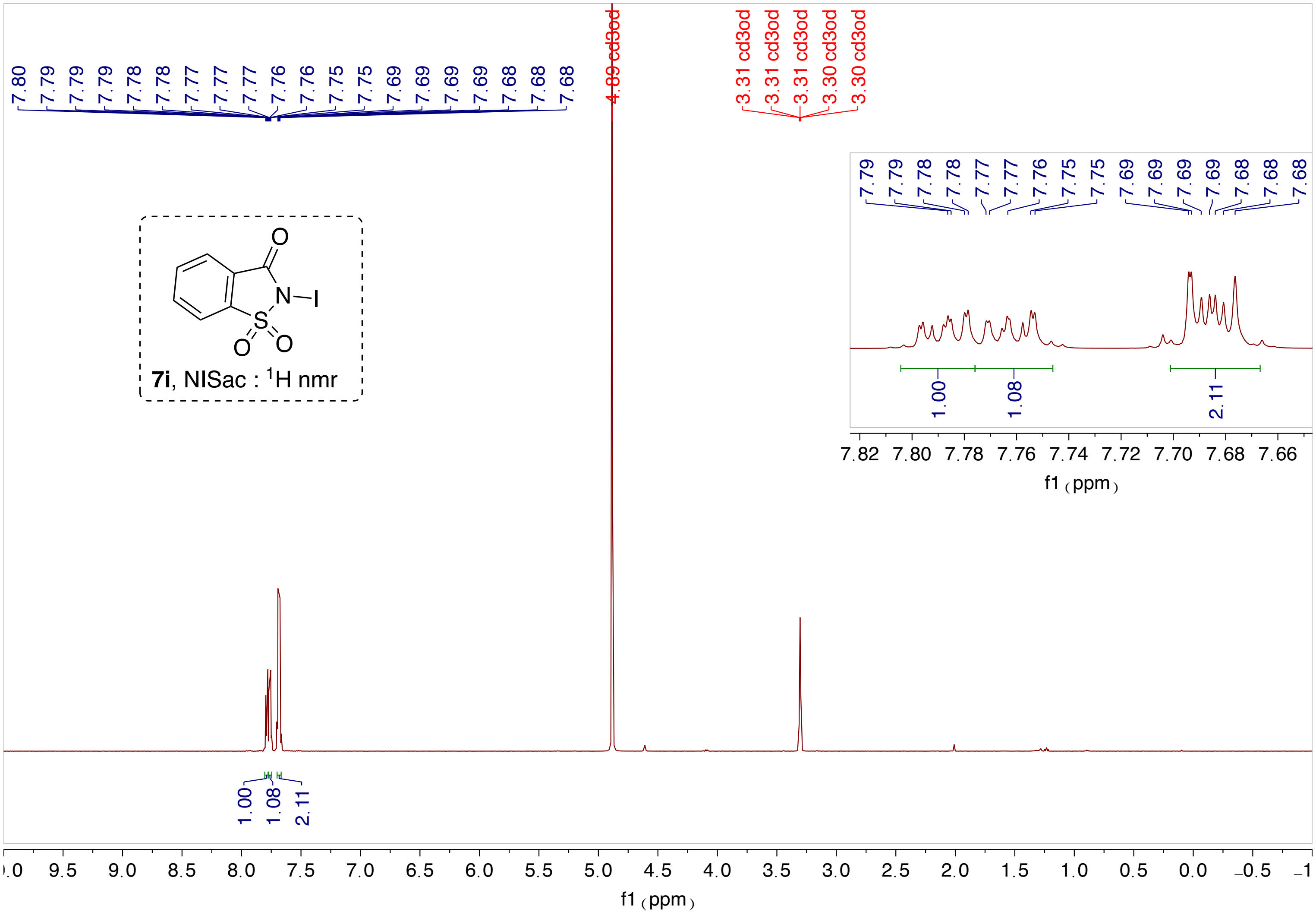

Figure S55: ^{13}C NMR of Compound **7i**: 2-iodobenzo[*d*]isothiazol-3(2*H*)-one 1,1-dioxide -^{13}C NMR (126 MHz, CD_3OD)

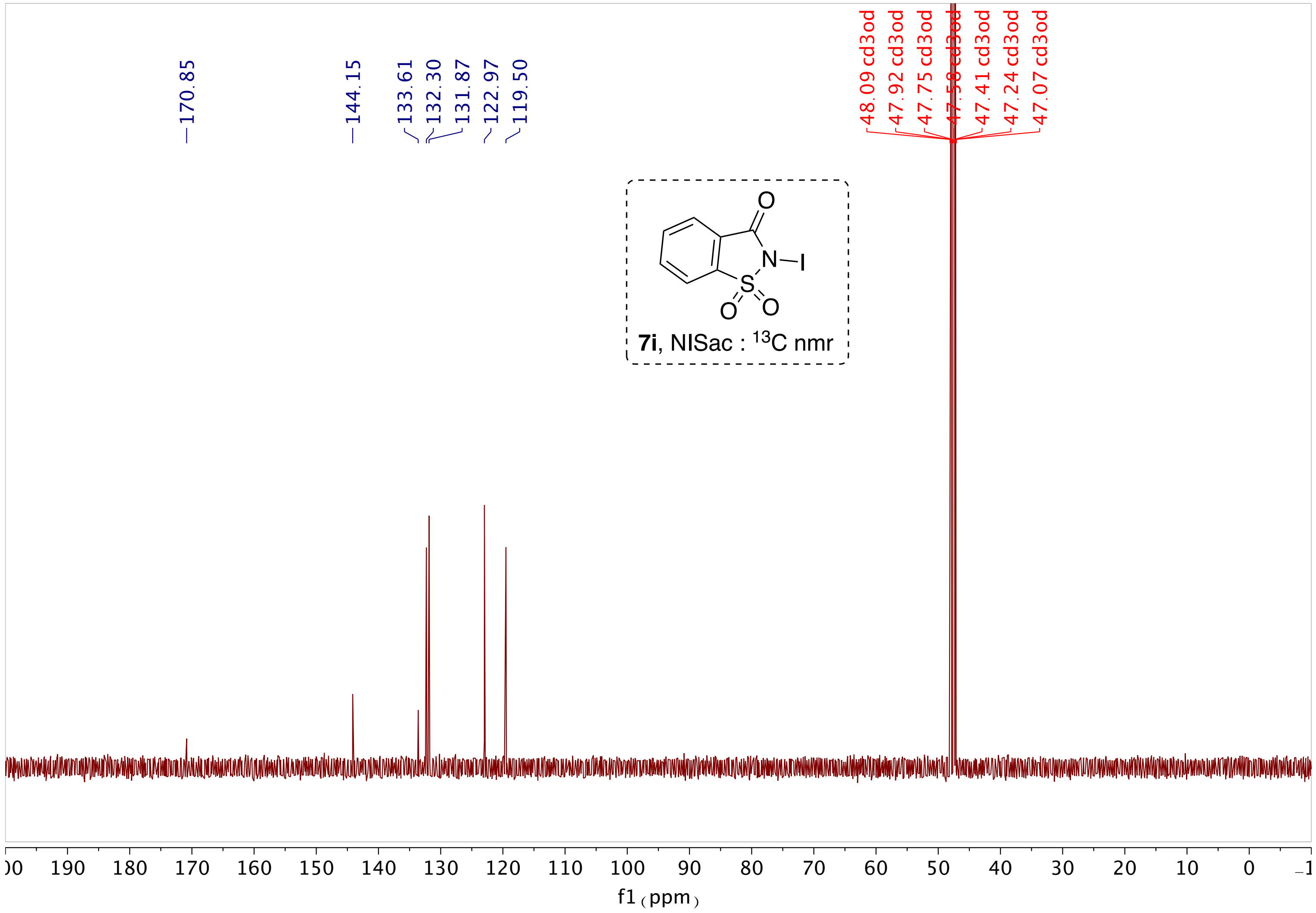

Figure S56: ^{1}H NMR of Compound **7j**: *N*,*N*-diiodo-4-methylbenzenesulfonamide-^{1}H NMR (500 MHz, CD_3OD)

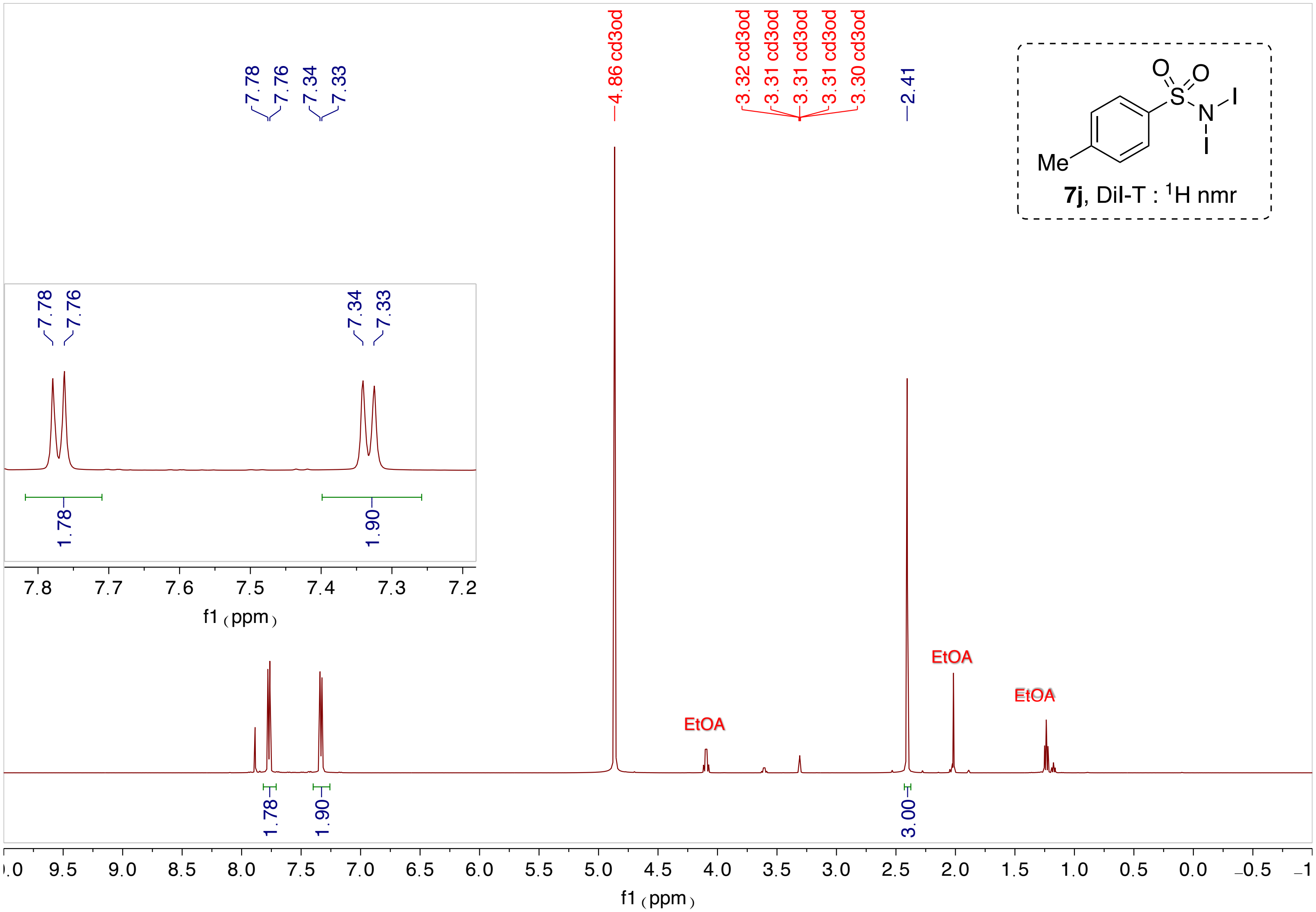

Figure S57: ^{13}C NMR of Compound **7j**: *N*,*N*-diiodo-4-methylbenzenesulfonamide-^{13}C NMR (126 MHz, CD_3OD)

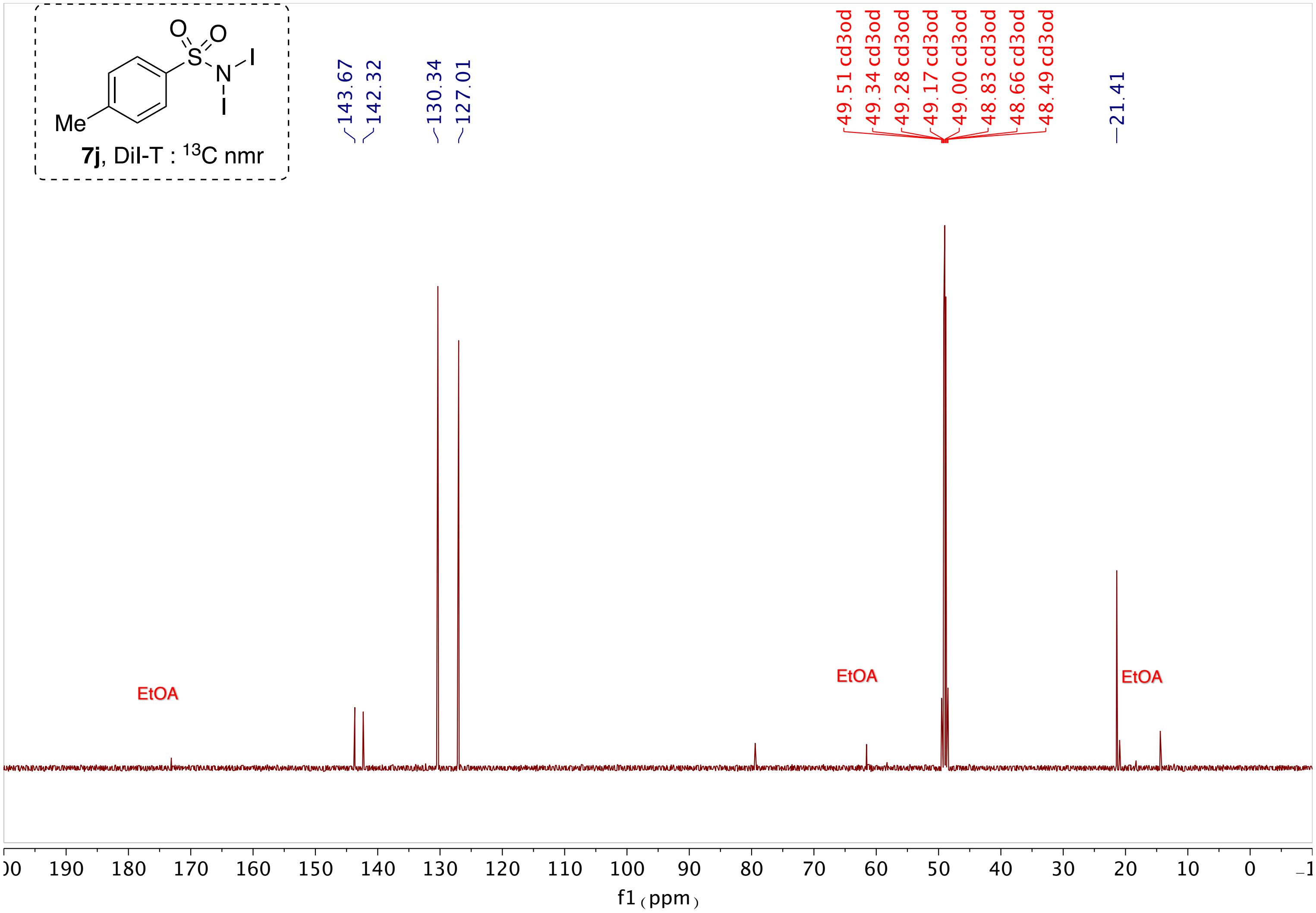

Figure S58: ^{1}H NMR of Compound **8**: 1-(2-bromo-1-chloroethyl)-4-methoxybenzene -^{1}H NMR (500 MHz, $CDCl_3$)

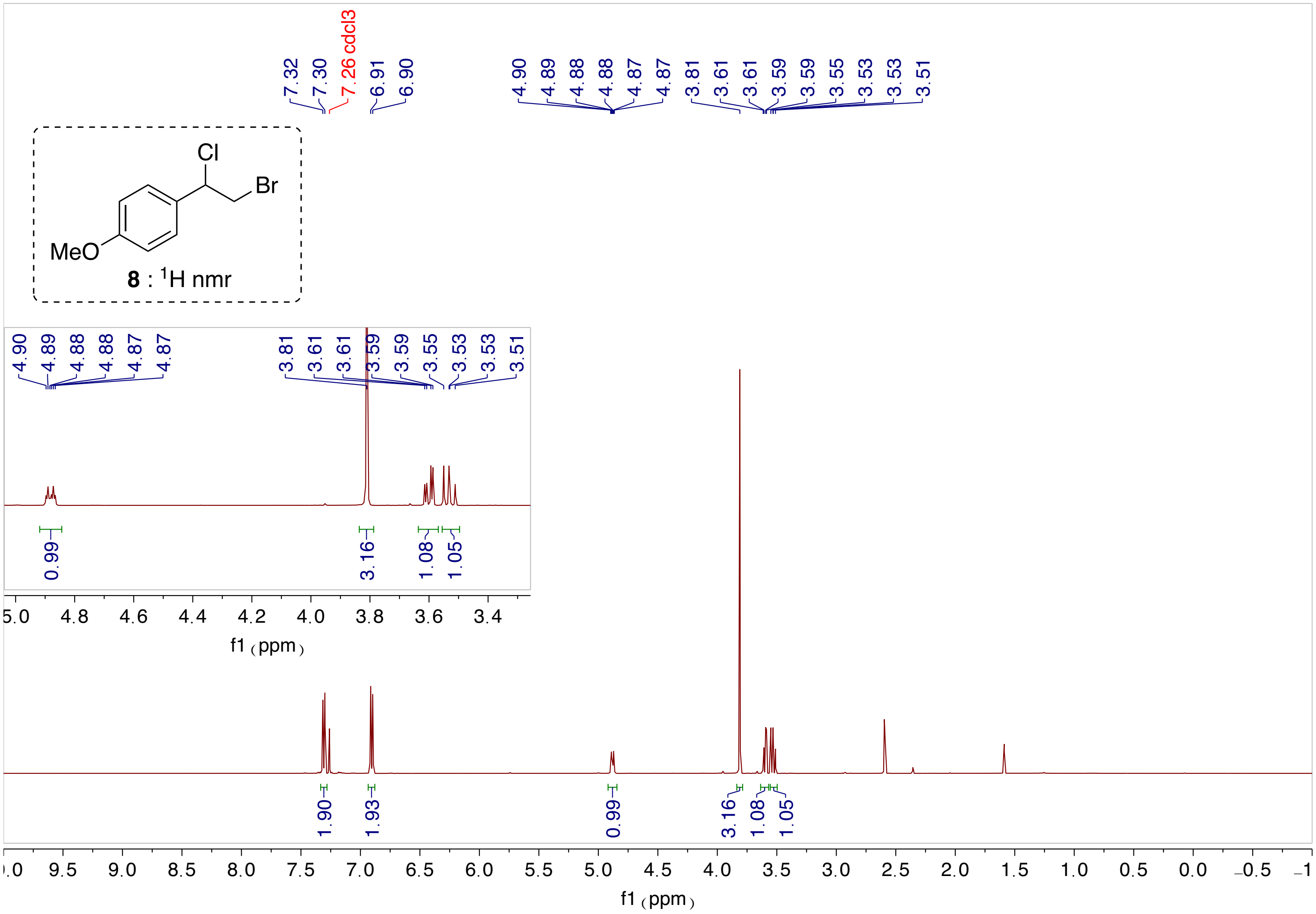

Figure S59: ^{13}C NMR of Compound **8**: 1-(2-bromo-1-chloroethyl)-4-methoxybenzene -^{13}C NMR (126 MHz, $CDCl_3$)

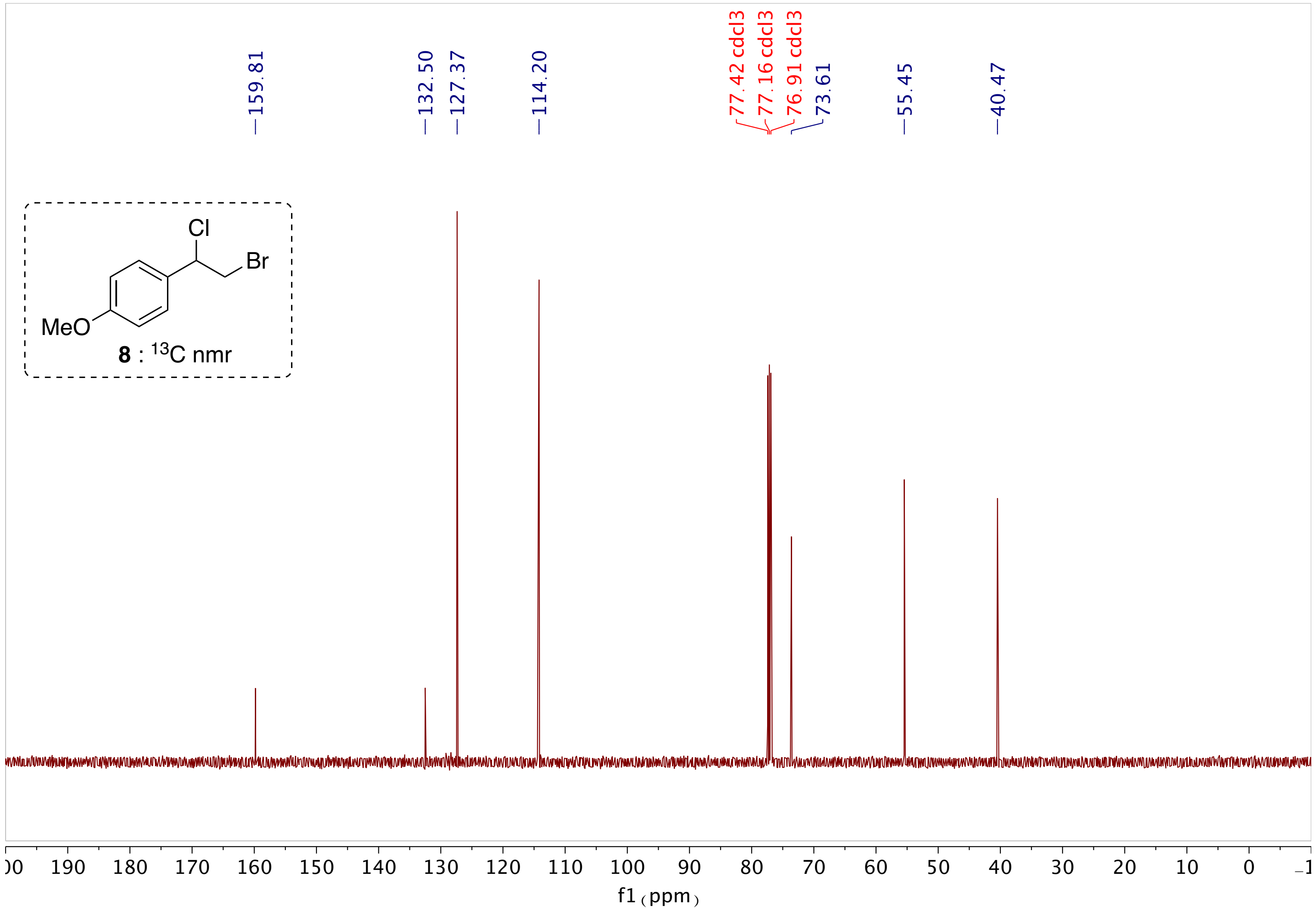

Figure S60: ^{1}H NMR of Compound **9**: 1-(1-chloro-2-iodoethyl)-4-methoxybenzene -^{1}H NMR (500 MHz, $CDCl_3$)

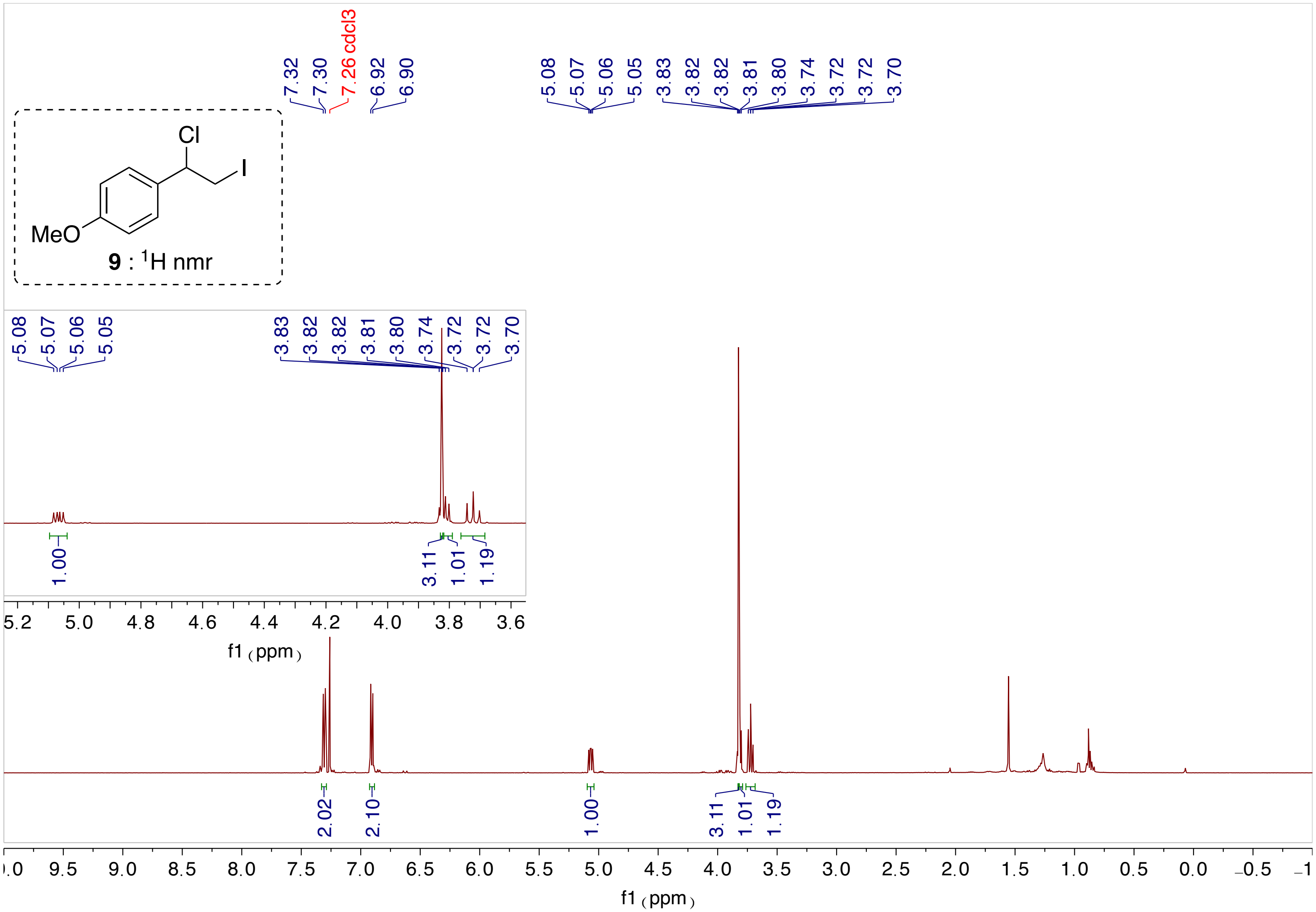

Figure S61: ^{13}C NMR of Compound **9**: 1-(1-chloro-2-iodoethyl)-4-methoxybenzene -^{13}C NMR (126 MHz, $CDCl_3$)

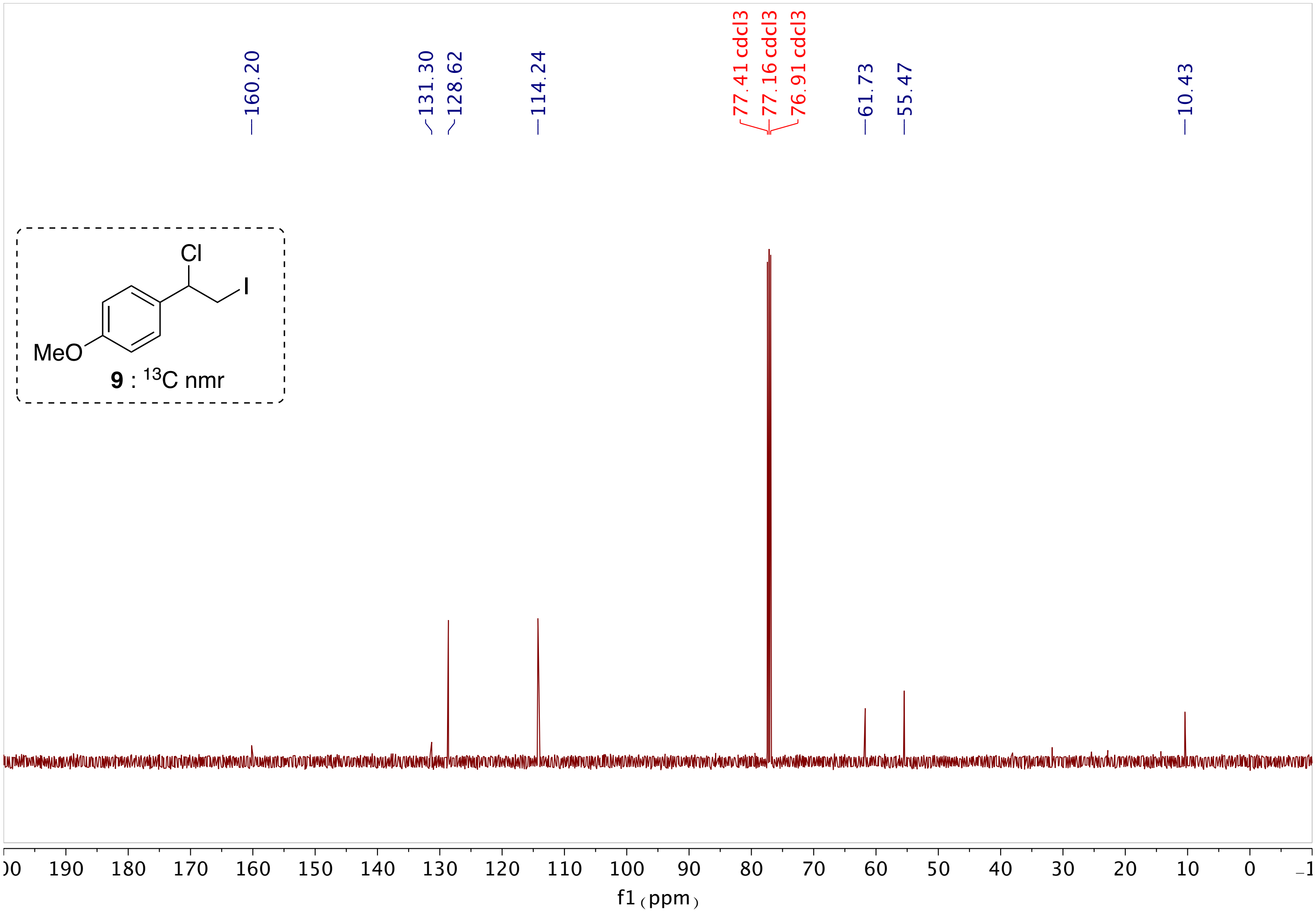

Figure S62: ^{1}H NMR of Compound **10**: 1-(2-bromo-1-chloroethyl)-benzene -^{1}H NMR (500 MHz, $CDCl_3$)

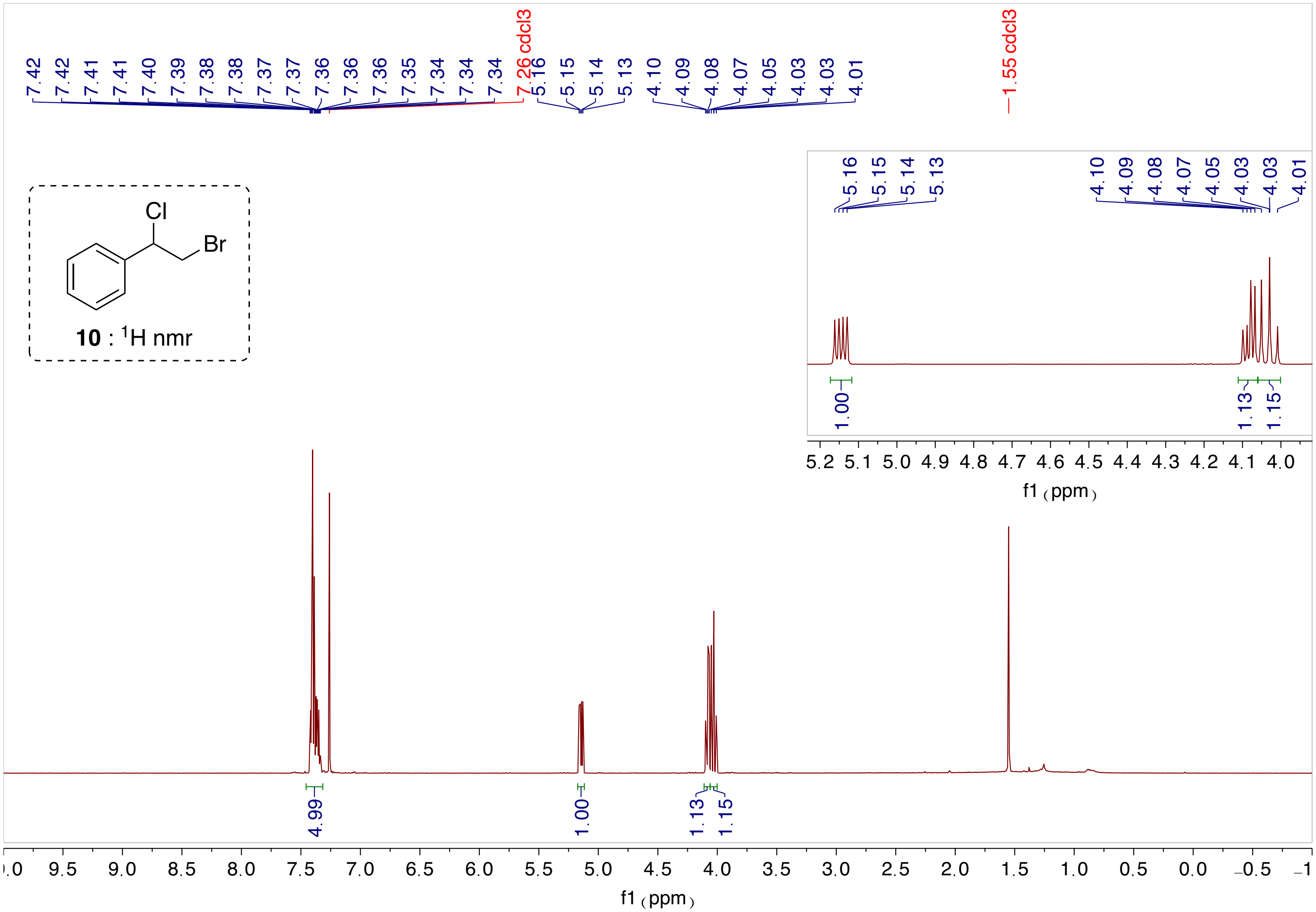

Figure S63: ^{13}C NMR of Compound **10**: 1-(2-bromo-1-chloroethyl)-benzene -^{13}C NMR (126 MHz, $CDCl_3$)

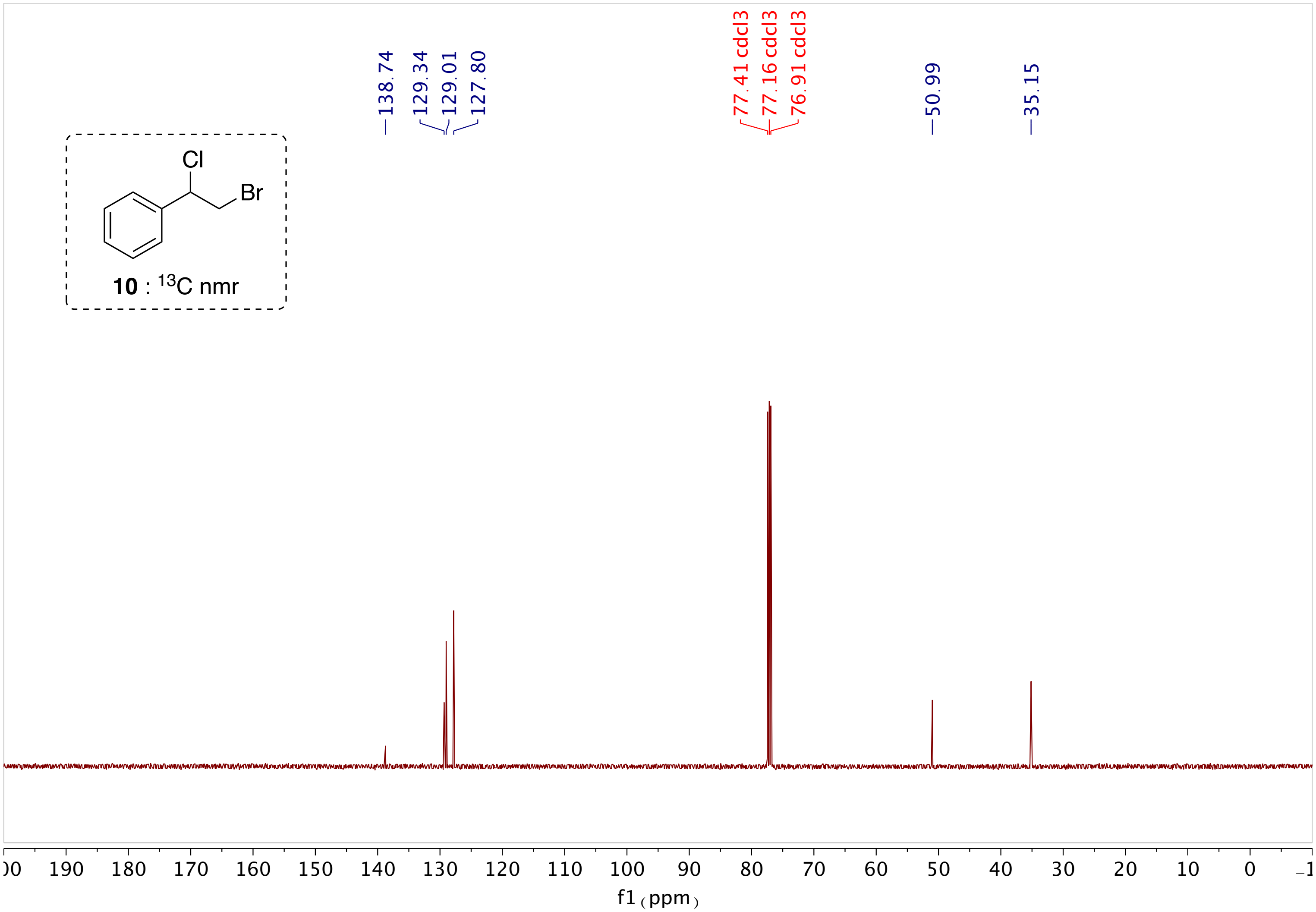

Figure S64: ^{1}H NMR of compound **11**: 1-(1-chloro-2-iodoethyl)-benzene -^{1}H NMR (500 MHz, $CDCl_3$)

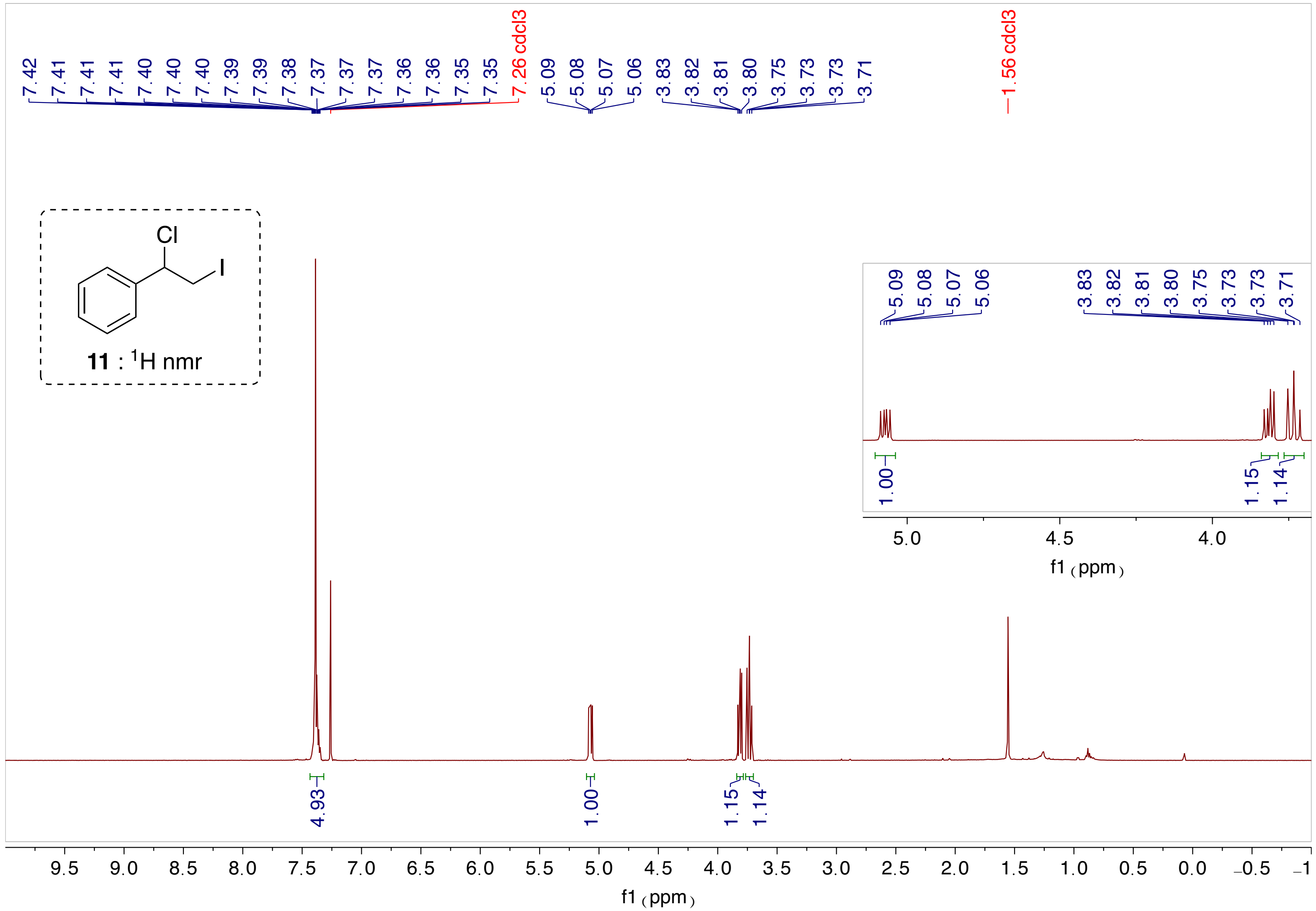

Figure S65: ^{13}C NMR of compound **11**: 1-(1-chloro-2-iodoethyl)-benzene -^{13}C NMR (126 MHz, $CDCl_3$)

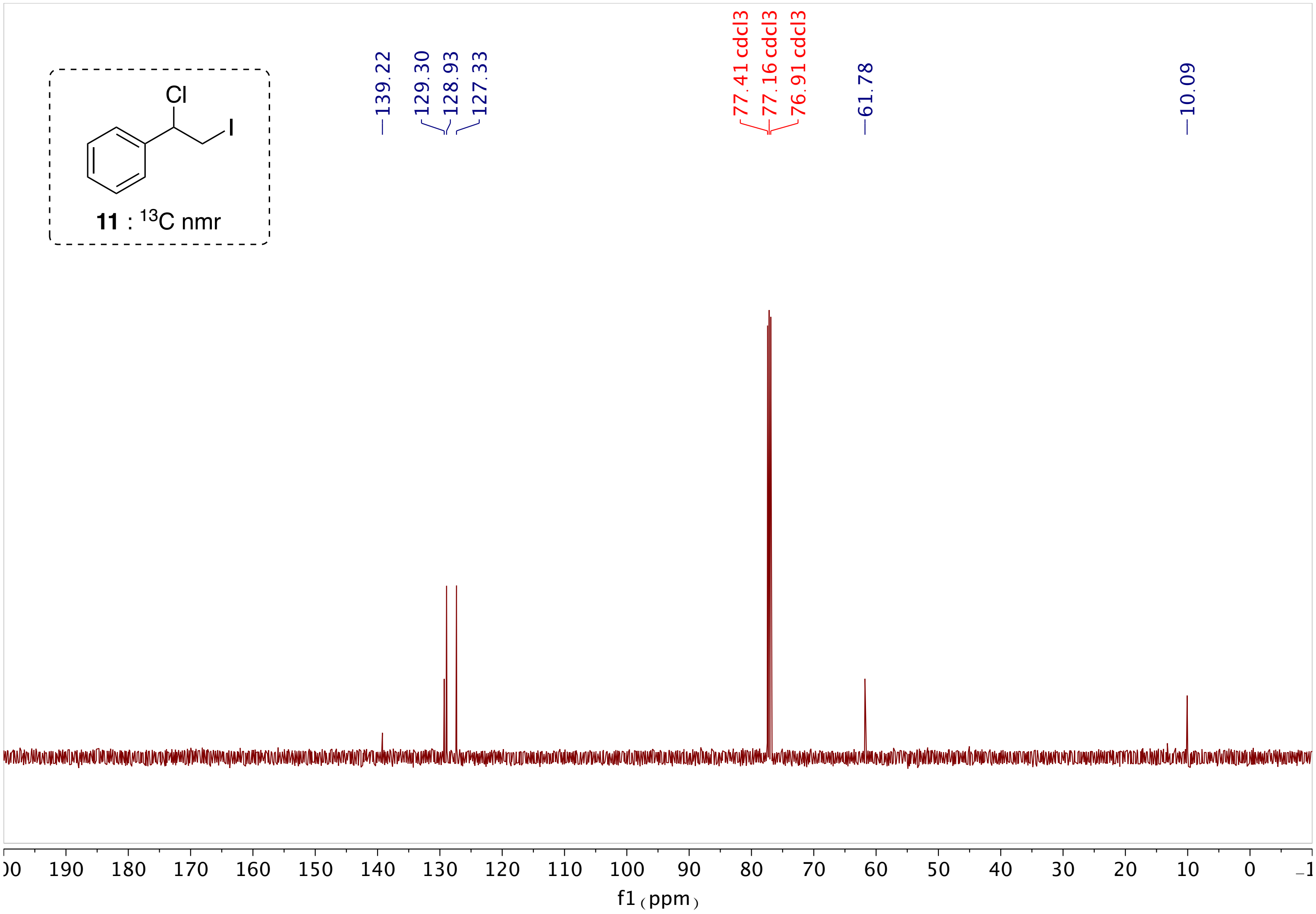

Supplement: Supplementary file 1 — op4c00194_si_001.pdf [file op4c00194_si_001.pdf]
